# Masakari: visualization supported statistical analysis of genome segmentations

**DOI:** 10.1186/s12859-020-03761-6

**Published:** 2020-10-07

**Authors:** Dirk Zeckzer, Alrik Hausdorf, Nicole Hinzmann, Lydia Müller, Daniel Wiegreffe

**Affiliations:** 1 Natural Language Processing Group, Department of Computer Science, University of Leipzig, Augustusplatz 10, 04109 Leipzig, Germany; 2 Bioinformatics Group, Department of Computer Science, University of Leipzig, Härtelstraße 16-18, 04107 Leipzig, Germany; 3 Image and Signal Processing Group, Department of Computer Science, University of Leipzig, Augustusplatz 10, 04109 Leipzig, Germany

**Keywords:** ChIP-seq, Chromatin state, Histone modifications, Cell development

## Abstract

**Background:**

In epigenetics, the change of the combination of histone modifications at the same genomic location during cell differentiation is of great interest for understanding the function of these modifications and their combinations. Besides analyzing them locally for individual genomic locations or globally using correlations between different cells types, intermediate level analyses of these changes are of interest. More specifically, the different distributions of these combinations for different cell types, respectively, are compared to gain new insights.

**Results and discussion:**

We propose a new tool called ‘Masakari’ that allows segmenting genomes based on lists of ranges having a certain property, e.g., peaks describing histone modifications. It provides a graphical user interface allowing to select all data sets and setting all parameters needed for the segmentation process. Moreover, the graphical user interface provides statistical graphics allowing to assess the quality and suitability of the segmentation and the selected data.

**Conclusion:**

Masakari provides statistics based visualizations and thus fosters insights into the combination of histone modification marks on genome ranges, and the differences of the distribution of these combinations between different cell types.

## Background

Every cell of an organism carries the same genome. However, each cell type shows a specific expression pattern. Epigenetic modifications were found to regulate the gene expression. Prominent examples are the trimethylations of the lysines of histone H3 at position 4 (H3K4me3) and at position 27 (H3K27me3) located near the transcription start sites of genes. While H3K4me3 correlates positively with the expression of genes and euchromatin formation, H3K27me3 induces heterochromatin formation and repression of transcription. Promoters associated with both marks are in the so-called poised chromatin state, i.e., transcription is repressed but can easily be switched on when needed [1–3].

Early research showed, that changes in the combination of marks occur frequently during development. Thus, they shape the cell type identity. Often, changes at the promoters are tracked and correlated with cell expression. However, some modifications do not show an immediate effect or are not primarily localized at promotors. For example, trimethylation at lysine 36 on histone H3 is associated with splicing [4]. Consequently, it is usually found at exon-intron boundaries. Changes in the methylation patterns during development would thus be missed with a promoter-centric approach. The exploration of genome-wide chromatin patterns and their changes should therefore be based on the genomic position rather than centered on promoters, genes, or other genomic entities.

We propose the tool “Masakari” for a data-driven segmentation of the genome: the segments are calculated based on the peaks describing chromatin modifications of genomic regions obtained using a peak caller like Sierra Platinum [5, 6]. Masakari implements the segmentation proposed by Steiner et al. [7]. They furthermore describe the transformation of raw data into the data used for segmentation and demonstrate the visual exploration of the chromatin changes using self-organizing maps. Further visualizations using 2D and 3D tiled binned scatterplots assuming the same segmentation data is presented by Zeckzer et al. [8–10], respectively.

However, one problem remained unsolved so far: the appropriate selection of reference and additional data sets. Appropriate selection of these data sets by the analyst ought to be supported by additional information about the resulting segmentation. At the same time, the analyst should be enabled judging whether or not to change the selected data sets. This leads to the additional requirements, (1) that the information can be computed without any additional knowledge besides the data provided, (2) that (re-)computing the information should be fast enough enabling a rapid exploration of the resulting segmentations, and (3) that the analysis of the additional information is supported by interactive visualizations.

Thus, besides providing an implementation of the aforementioned segmentation process, Masakari provides (1) several simple and fast analysis methods of the segmentation obtained and of the additional data sets together with (2) interactive visualizations allowing to judge whether the selected combination of data sets exhibits patterns by means of systematic changes in the chromatin modifications. Masakari fosters the analysis of the modification state. Furthermore, plots of the distribution of peak lengths and of segment lengths support the analysis of these distributions. Additional data, such as further modifications, can be compared to the modifications used as reference by means of coverage. Moreover, analyzing exact sequence patterns and motifs in the segments are supported by our tool. While designed for being used for chromatin modifications, i.e., peaks from a peak caller, the data format requires genomic position in BED format, only. Thus, Masakari allows for segmenting other data types, too.

As Masakari aims at an early stage of analysis, we refrained from providing more complex analysis methods. Those are available in the form of more informative visualizations [8–10] supporting testing sets of histone marks in different cell types for correlations within a cell type and coherent switching into other combination of marks in other cell types. Furthermore, we do not restrict analysis to specific regions with known function, while annotation of functional regions can be provided as additional data allowing testing their association with combinations of marks.

Instead of using visualization for *interactively* analyzing the resulting segmented data, machine learning techniques could be employed for *automatic* analysis. Moreover, machine learning techniques such as hidden Markov Models (see Ernst et al. [11] for example), could be used for generating the segmentation. As those require prior knowledge about functional regions and the potential combination of marks associated with them and both—functional regions and the set of histone marks—have to be carefully chosen before applying such an automated analysis, our approach requires less knowledge and is more general than the former. In fact, the analyst may choose examining the chromatin states of a subset of the data set as well as freely choose the machine learning technique (e.g., hidden Markov models) to be applied.

## Implementation

Here, we describe the architecture of the software and the main elements of the methodology proposed. In the subsequent section ‘Results’, we describe a use case showing how to apply the methodology to obtain insights about chromatin changes during embryonic development.

### Architecture

Masakari is completely written in JAVA 11 and uses JavaFX as API for the GUI. It is deployed as runtime image for Windows, Linux, and MacOS. Masakari is implemented as a server client model to support a variety of usage scenarios. While the server holds the data and performs the calculations, the client uses the calculated data to generate the corresponding visualizations and handles the user interactions. It is possible to run server and client on the same machine. Even more, a server on the same machine can be started using the graphical user interface of the client. In this case, the client automatically connects to the server instance. Likewise, it is possible to start server and client on different machines and to connect them using TCP/IP connections. Furthermore, it is possible to execute Masakari in common batch environments using a job configuration file. This job configuration file can be created using the client by first setting all parameters and then exporting the selected parameters to a configuration file.

### Methodology overview

The complete methodology—including visualizations and interaction facilities for quality assessment and preliminary analyses, the interface of the tool, as well as technical details—is described in the Additional file 1.

Here, we focus on describing the algorithms for computing the segmentation and additional information, and the visualizations provided for analyzing this information as well as how to interpret these visualizations. Please note, that any of the statistics computed is purely descriptive. Neither counts nor logarithmic counts allow to judge whether any of the computed statistics is significant or not. Consequently, it can not be assessed whether any noticeable effect occurs due to an underlying mechanism or purely due to chance. This especially holds for overlaps found with Masakari that can be tested for statistical significance using an appropriate method such as GINOM [12]. Due to GINOM’s large runtime, we suggest to test only combinations showing a strong overlap in Masakari using such a method.

### Segmentation

The segmentation in general has been described before [7–9]. The input for the segmentation consists of a reference genome and the peaks obtained for the marks H3K4me3, H3K9me3, and H3K27me3 of the H1 embryonic stem cells. Each peak of each mark represents a range of nucleotides on the reference genome. Computing the intersection of the ranges of different marks results in maximal ranges where either no mark is present, only one mark is present, two marks are present, or all three marks are present (Additional file 1: Figure 1.2). These maximal ranges are the *segments* generated. Two adjacent segments thus always differ in the combination of marks overlapping these segments or the complete absence of any mark overlapping a segment. The information associated with each segment is its combination of marks and its length. Each combination of marks defines a category and is mapped to its code (Additional file 1: Section 1.3).

**Fig. 1:**
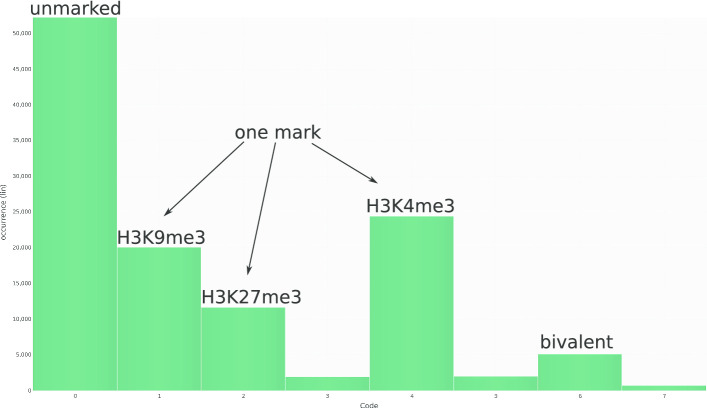
Code frequency: each code is mapped to a bar, while each code’s frequency is mapped to the height of the bar. Most segments are not modified (code 0; leftmost bar), or are only modified by one mark (codes 1, 2, 4), while only few segments carry two (codes 3, 5, 6) or three (code 7) marks

The analysis of the segments computed is supported by several visualizations. In the code frequency plot, a bar chart is used mapping each code to a bar and each code’s frequency to the height of the bar (see Fig. 1).

**Fig. 2:**
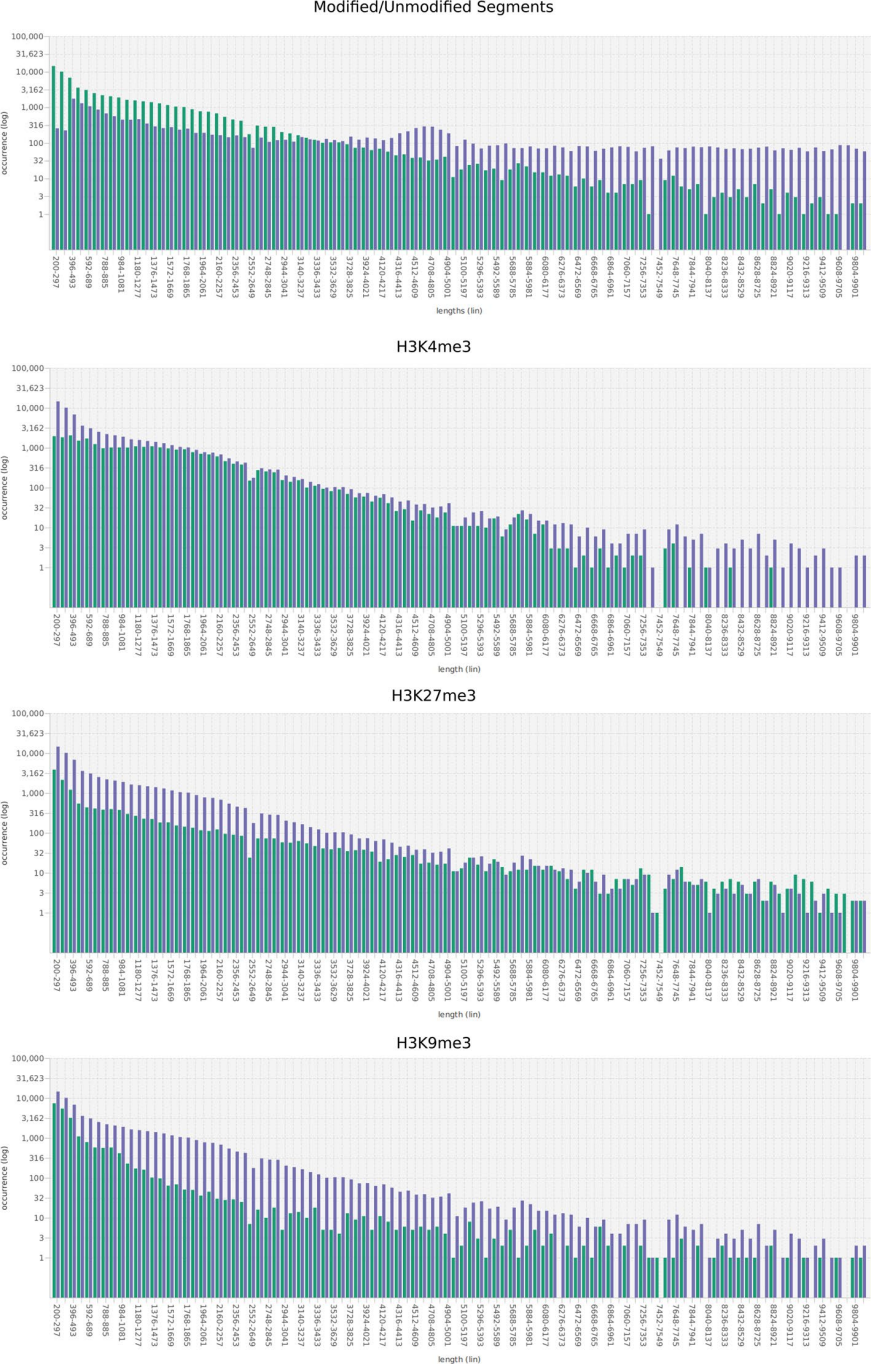
Distribution of segment lengths. Top panel: Distribution of the lengths of *modified segments* (violet) and of *unmodified segments* (green). Three bottom panels: Distribution of the lengths of *peaks* having the respective mark (violet) and of *segments* having the respective mark (green), only. All frequency axes are scaled logarithmically

The distribution of the segments lengths is also shown using bar charts (Fig. 2). In this case, a bar shows how often a specific length or a range of lengths occurs. Drawing two bars for each length or range of lengths enables to compare two distributions. Each distribution is drawn using its own color.

**Fig. 3:**
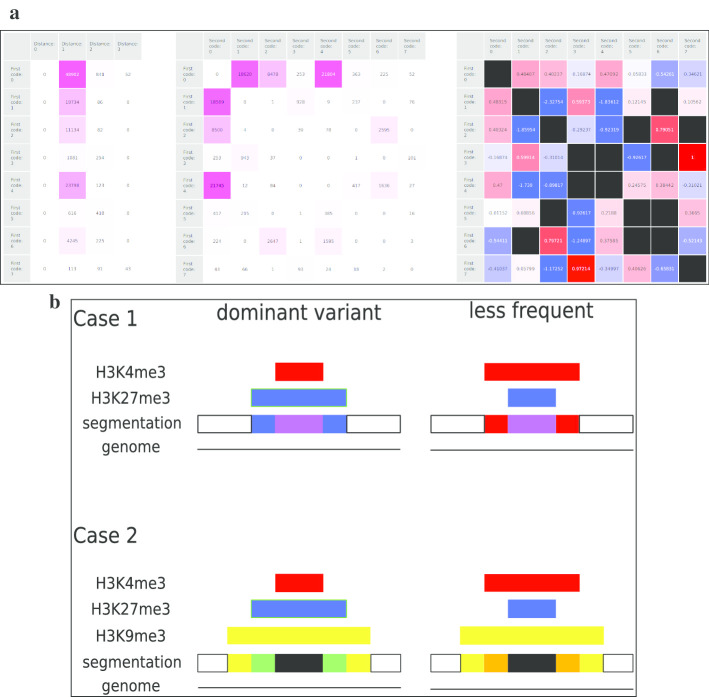
Adjacent segment analysis. **a** Adjacent segment pairs. Row: code of the 5′-segment, one row per code. Left: Distance in number of modification differences of the epigenetic code on H1. Columns represent the distances (0–3) between the code of the 3′-segment (column) and the code of the 5′-segment (row). Middle: Frequency of adjacent segments with specific code combinations. Columns represent the code of the 3′-segment. Right: Observed-vs-expected frequencies of mark combinations of adjacent segments. Expected frequencies are calculated based on the code frequencies in the data sets. Columns represent the code of the 3′-segment. Blue colors indicate combinations that are observed less often than expected while red colors indicate combinations that are observed more often than expected. Stronger saturation implies larger distance from equality. Black indicates combinations not observed at all. **b** Over-represented peak arrangements: (Case 1) More often than expected by chance, H3K4me3 peaks are located within an H3K27me3 domain. Less frequent but still more often than expected by chance, H3K27me3 peaks are located within an H3K4me3 peak. (Case 2) We also observe Case 1 being located within a large H3K9me3 domain more often than we would expect by chance

To support the analysis of adjacent segment pairs, three heatmaps showing different information are used (Fig. 3a). The first heatmap (left) relates the code of the 5′-segment (row) to the distance of the 3′-segment’s code (column). The second heatmap (middle) relates the code of the 5′-segment (row) directly to the code of the 3′-segment (column). In both cases, a pink color is used for showing the amount of such relations qualitatively. The higher the amount of segments with the respective property, the more saturated (colorful) the cells are. The third heatmap (right) relates the code of the 5′-segment (row) directly to the code of the 3′-segment (column), too. However, the color mapping now describes quantitatively if the observed frequency of two adjacent marks is higher or lower than the expected frequency. If the observed frequency is higher than the expected frequency, then the cell is colored blue. If the observed frequency is lower than the expected frequency, then the cell is colored red. Again more colorful cells point to a large difference of the observed frequency to the expected frequency. White cells represent observed frequencies that match the expected frequency. Cells are colored black, if there are no pairs expected (diagonal) or observed. Thus, they are distinguishable from both colored or (near to) white cells.

### Segment–short segment–segment triplets

Masakari allows to analyze the modification changes of segment–short segment–segment triplets. If there would be many combinations of two segments, which are adjacent to the same short segment and which carry the same combination of marks, then this would indicate a problematic segmentation. This situation occurs only, if the two segments are separated by short peaks that partially overlap. Analyzing these triplets is supported by heatmaps similar to those used for the analysis of segment pairs. The only difference is, that each row represents a distinct combination of modifications of the two enclosing segments, while each column represents either a distinct distance of the combination of modifications of the short segments or a specific combination of modifications of the short segments. Moreover, the length distribution of the short segments of such triplets can be analyzed using bar charts similarly to the length analysis of segments (see Additional file 1: Figure 1.17).

### Fate-of-code computation

Peaks of the marks of other cell types can be mapped onto the segments generated using the peaks of the marks of the reference cell type (Additional file 1: Section 1.5 and Figure 1.2). These marks can be the same or different ones. Therefore, the files containing those peaks are selected and the coverage of the segments by the respective peaks (regions on the genome) are computed. This coverage value lies between 0 and 1.

For the fate-of-code computation, it is determined for each segment, if the mark is present for the reference and for the additional cell line. Therefore, the marks selected for the additional cell line have to be the same as those of the reference cell line. As the coverage value might differ from both 0 and 1, a threshold is selected by the analyst. This threshold determines whether the segment is supposed having the mark (coverage above the threshold) or not (coverage below the threshold). Then, a code for the additional cell line is computed in the same way as for the reference cell line.

**Fig. 4:**
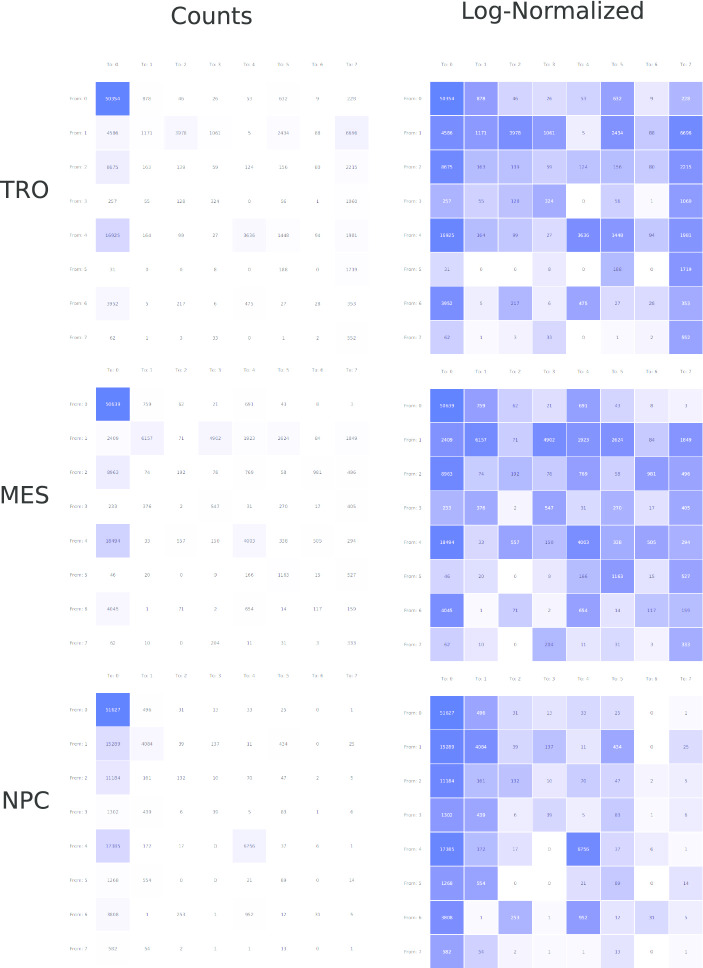
Fate-of-code: for each segment of the H1 segmentation, the corresponding code for the three cell types TRO, MES, and NPC with threshold 0.8 is calculated. Changes and conservation of the H1 code (0–7, rows) in these cell types are shown in the heatmaps for TRO (top panel), MES (middle panel), and NPC (bottom panel). The heatmaps are colored by the raw counts (left panel) and by the logarithm of the counts (right panel). Rows: code for H1, columns: code for TRO, MES, and NPC, respectively. High correlation: strong saturation; low correlation: low saturation

The analysis of fate-of-code is supported by heatmaps as well (Fig. 4). The encoding is similar to the heatmaps for the pairs analysis: one row per code of the reference cell type (here: H1) and one column per code of the additional cell type (here from top to bottom: TRO, MES, and NPC). A cell represents the number of segments (counts) having the code of the reference cell type (row) and having the code of the additional cell type (column). Small variations might be missed when using a linear scale for the counts (left column, raw counts). Therefore, a logarithmic transformation can be applied to the counts before mapping them to color (right column). As before, the (logarithmically transformed) values are mapped to the saturation of the color.

### Correlation

The correlation between all pairs of attributes available for a segment—code, segment marks (H1), additional marks (TRO, MDD, MES, NPC), CTCF motifs, CpG density, and experimental CTCF data—can be computed. The correlation coefficient computed is Spearman’s rank correlation coefficient adjusted for ties. The latter is very important, as the data might contain a huge amount of ties.

**Fig. 5:**
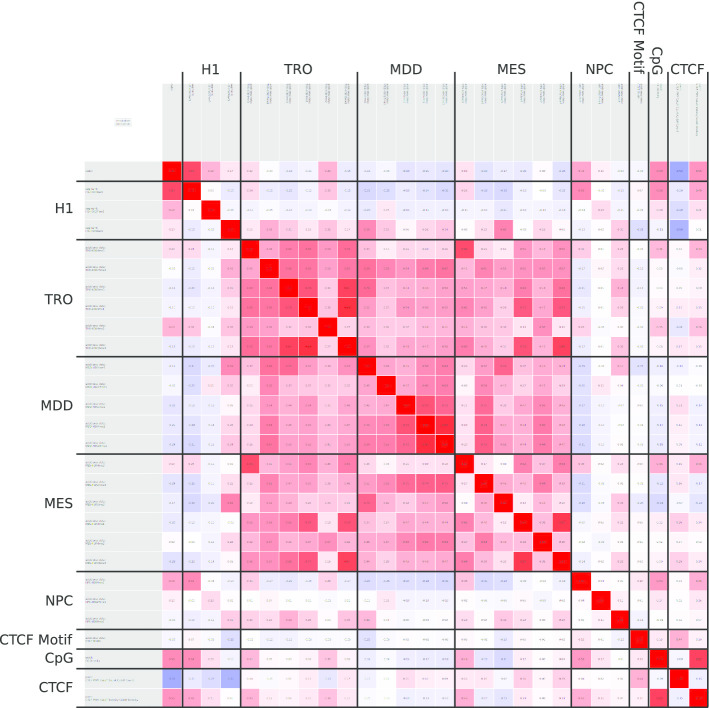
Correlation analysis: correlation between each pair of data sets as well as the code (first row and column) and the CpG-density (last row and column). Horizontal and vertical lines indicate blocks of modifications of the same cell types. Blocks of cell types are labeled before and above the blocks. Red: positive correlation, blue: negative correlation. High saturation: strong correlation; low saturation: weak correlation

A heatmap fosters analyzing these correlations qualitatively and quantitatively (Fig. 5 and Additional file 1: Section 1.8 including Figure 1.29 therein). Each attribute is assigned to one row and to one column in the heatmap. The heatmap cells show the correlation value between the attributes of the corresponding row and column and are colored accordingly. If the observed correlation is positive, then the cell is colored red. If the observed frequency is negative, then the cell is colored blue. Again more colorful cells represent larger correlation coefficients (absolute value). White cells represent correlation values that are 0; whitish cells correlation values that are close to 0.

Thus, the correlation analysis plot (Fig. 5) allows investigating the whole data set as one unit. Strongly correlating data sets indicate, that the data is very similar and differs only in a small fraction of the segments. Low correlations or even anti-correlations provide evidence for unrelated data sets (attributes). In such a case, one may rethink the choice of the cell types and features.

## Results

We show how using Masakari supports analyzing chromatin changes during embryonic development.

### Data

From the NIH Roadmap Epigenomics projects, we downloaded data for 5 cell types: H1 embryonic stem cells (H1), trophoblast cells (TRO), mesendodermal cells (MDD), mesenchymal stem cells (MES), and neuronal progenitor cells (NPC). We selected the modifications H3K4me3, H3K27me3, and H3K9me3 since they are reported to play an important role in the cell development. Only for MDD, there is no H3K9me3 data set available. For TRO, MDD, and MES we also downloaded H3K4me1 and H3K4me2 as well as H3K26me3. Details on the data preparation can be found in the Additional file 1.

We used Masakari to segment the human genome hg19 based on the peaks from all three H1 data sets. As additional data, we added the peaks for the modifications of TRO, MDD, and MES as well as peaks for CTCF binding sites in H1 from Encode [13]. Furthermore, we calculated the CpG-density. Finally, the position weight matrix for the CTCF binding sites from Kim et al. [14] was used to calculate count and density of potential binding sites of CTCF within the segments.

### Segmentation analysis

The segmentation results in 117,774 segments covering about 3.1Gbases. During segmentation 6297 short segments whose length is smaller than 200nt were discarded. This amounts to 567Mbases that were discarded. As a result 190 peaks were lost. Discarded segments are either 50, 100, or 150 nucleotides long showing the uncertainty of the true start and stop of modified peaks resulting from peak calling. Furthermore, mostly only single short segments can be found between segments that are at least 200nt long. There are less than 400 cases where several short segments build a chain and most chains are shorter than 300 nucleotides, the longest chain of discarded segments being 600 nucleotides long. In summary, a large part of the data is retained, discarded segments do not enrich at specific positions, and a further analysis of the segmentation is useful. The corresponding plots showing this result can be found in the Additional file 1: Figure 3.1 and Figure 3.2, respectively.

Most segments are unmodified in the embryonic stem cells [15] (see Fig. 1). Among the modified segments, modifications with only one mark are most frequent and the most frequent modification is H3K4me3 (see Fig. 1). Bivalent segments (marked with H3K4me3 and H3K27me3) are most frequent among the segments with multiple marks (see Fig. 1, bar labeled as bivalent and unlabeled bars). Thus, there are indeed different combinations of those three marks which can be investigated further using the segmentation.

As expected, short segments are more frequent than long segments. There is, however, a slight enrichment of segments with a length between 4500 and 5000 nucleotides. This enrichment is specific for unmodified segments (see Fig. 2 top). H3K27 and H3K9 trimethylation peaks are usually retained during segmentation (see Fig. 2). Peaks of H3K4me3 however are often broken apart into short sub-peaks (see Fig. 2).

Independently of the combination of marks, adjacent segments differ usually by only one modification (see Fig. 3a, left). Furthermore, specific patterns can be observed using the heatmap of the counts of adjacent segments (Fig. 3a, middle) and the log-odds of the observed-expected ratio (Fig. 3a, right). The current data set suggests two symmetric patterns (Fig. 3b): (Case 1) An unmodified segment is followed by a segment marked with H3K27me3 only which in turn is followed by a segment marked with H3K27me3 and H3K4me3. The latter is again followed by a segment marked with H3K27me3 only which in turn is followed by an unmodified segment. In other words, an H3K4me3 peak is embedded in an H2K27me3 domain. In other cases, this might be the opposite, i.e., an H3K27me3 peak being embedded in an H3K4me3 peak. (Case 2) Segments modified by all three marks are usually surrounded by segments with H3K9me3 and either H3K4me3 or H3K27me3. Next to those segments, segments marked with H3K9me3 only or H3K4me3 (H3K27me3) only are found. The left and the right end of this pattern are again unmodified segments. Here, too, peaks are supposedly embedded within each other. With respect to the observed/expected ratio, the most likely pattern is a H3K4me3 peak embedded in an H3K27me3 peak which is itself embedded in a H3K9me3 peak. Please note, that the second pattern is the first pattern embedded in an H3K9me3 peak. Both pattern consist of combinations of adjacent segments that are much more often observed than expected by chance. Thus, they may be based on molecular mechanisms such as recruitment of modifiers by sequence motifs.

### Fate-of-code analysis

For all cell types except mesendodermal cells (due to missing data for H3K9me3), we calculated how combinations of modifications change during development (see Fig. 4). It is noteworthy, that H3K9me3 marks are the strongest retained modifications in all three cell types. Segments carrying H3K9me3 in H1 acquire frequently H3K4me3, H3K27me3, or both marks in trophoblast cells and in mesenchymal stem cells. An interesting trend in trophoblast cells is furthermore an increase in segments with all three modifications. However, the specific biological function of this combination is currently unknown.

### Correlation analysis

Analyzing the correlation between all available attributes (Fig. 5) shows, that the data sets for segmentation correlate at least in some cases with the additional data. At least the same modifications in different cell types mostly correlate. Further analysis will therefore provide insights into the fate of such a modification during embryogenesis.

Moreover, correlations with CpG-density show interesting patterns: CpG-density correlates with H3K4me3 in all cell types except MDD and with H3K27me3 in H1. This provides evidence for a CpG-dependent recruitment. H3K9me3 in MES anti-correlates with the CpG-density which can result from a recruitment mechanism avoiding CpGs.

## Discussion

Overall, the collection of cell types and modifications as well as their segmentation allows investigating the changes of modifications during embryogenesis. The results obtained in the previous section show, that it is possible to analyze more than three modifications of the reference cell type and more than the 7 additional data sets (three modifications in two cell types each plus CpG) as well as the segment length used in the original publication of the segmentation method [7]. Here, segment length, four additional cell types with overall 20 modifications as well as CpG-density, CTCF motif, and experimental CTCF data were analyzed and compared to three modifications for the reference cell type H1.

In principle, even more modifications for more cell types could be analyzed. Here, the analytical effort grows linearly with the number of modifications or additional cell types; quadratically, if their product (#(modifications) × #(cell types)) is considered. Only the heatmap grows quadratically in the number of attributes (modifications, CTCF, CpG, and other data). As already outlined in the introduction, more sophisticated methods like visualizations targeting the analysis of histone modification data, automatic methods based on machine learning, and combinations of machine learning and visualization can and probably should be used, once an overview over the data is obtained and interesting attributes were selected based on Masakari. Moreover, effects observed using the more sophisticated methods should also be tested for statistical significance to reduce false discoveries due to data quality. The only parameter that results in an exponential growth is the number of modifications of the reference cell type. Here, a maximum of four or five modifications (16 and 32 combinations, respectively) appears to be feasible for analysis. However, Masakari would allow identifying frequently and less frequently occurring combinations of marks (including no and individual marks), which in turn would allow selecting those individual marks and combinations of marks of interest to the analyst for further examination.

## Conclusion

We presented Masakari, a tool for combining histone modification information by generating a segmented version of a reference genome, and the results obtained by segmenting the human reference genome hg19 based on the H1 embryonic stem cell modifications H3K4me3, H3K27me3, and H3K9me3. The method allows to segment the genome, to map additional data onto the segments—like modifications from other cell types—, compute motif and position weight matrix coverage of the segments, as well as compute the correlation among all pairs of data. Analyzing the results of the segmentation process and the coverage of the segments by modifications of other cell types gave new insights into the changes of histone modifications and their combinations during embryogenesis.

## Availability and requirements

- Project name: Masakari.
- Project home page: https://github.com/sierraplatinum/masakari for the source code and releases or https://zenodo.org/badge/latestdoi/99812760 (10.5281/zenodo.840853) for releases only.
- Operating system(s): Platform independent.
- Programming language: Java.
- Other requirements: Java runtime version 8 for Masakari version 1.0 and none for Masakari version 1.0b.
- License: Apache License 2.0.
- Any restrictions to use by non-academics: None.

## Supplementary information

**Additional file 1**. The supplementary information (pdf file) contains a detailed description of the method implemented in Masakari, especially of the input data selection and of the computation, Further, the technical details about the system and its graphical user interface are provided. There, the format of the resulting data is described, too (Section 2.3.4, ‘Export’). Finally, additional figures for the results described here are provided in the supplemental. These comprise the figures about the short segment chains (Section 3.1).

## Data Availability

The processed bed files used for the analysis are available at https://github.com/sierraplatinum/masakari-data.

## Abbreviations

H1: H1 embryonic stem cells
MDD: Mesendodermal cells
MES: Mesenchymal stem cells
TRO: Trophoblast cells
NPC: Neuronal progenitor cells
ChIP-seq: Chromatin immunoprecipitation sequencing
H3: Histone H3
K4: Lysine (one letter code K) at position 4
me1: Monomethylation
me2: Dimethylation
me3: Trimethylation
H3K4me1: Monomethylation at histone H3 at the lysine at position 4
H3K4me2: Dimethylation at histone H3 at the lysine at position 4
H3K4me3: Trimethylation at histone H3 at the lysine at position 4
H3K9me3: Trimethylation at histone H3 at the lysine at position 9
H3K27me3: Trimethylation at histone H3 at the lysine at position 27
H3K36me3: Trimethylation at histone H3 at the lysine at position 36
PCR: Polymerase chain reaction
