# Supplementary material for: Masakari: visualization supported statistical analysis of genome segmentations
Source: BMC Bioinformatics. 2020 Oct 7;21:437. doi: 10.1186/s12859-020-03761-6 (PMC7542120; doi:10.1186/s12859-020-03761-6)
Supplement: Supplementary file 1 — Additional file 1. The supplementary information (pdf file) contains a detailed description of the method implemented in Masakari, especially of the input data selection and of the computation, Further, the technical details about the system and its graphical user interface are provided. There, the format of the resulting data is described, too (Section 2.3.4, ‘Export’). Finally, additional figures for the results described here are provided in the supplemental. These comprise the figures about the short segment chains (Section 3.1). [file 12859_2020_3761_MOESM1_ESM.pdf]

Masakari:

Dirk Zeckzer[*⋈♣],
Alrik Hausdorf[*⋈♠△], Nicole Hinzmann[*⋈★△],
Lydia Müller[∘⋈♢], and Daniel Wiegreffe[∘*⋈♡]
[*] Image and Signal Processing Group
[∘] Bioinformatics Group
[⋈] Department of Computer Science, Leipzig University, Germany
[♠] Email: hausdorf@informatik.uni-leipzig.de
[★] Email: hinzmann@informatik.uni-leipzig.de
[♡] Email: daniel@bioinf.uni-leipzig.de
[♢] Email: lydia@bioinf.uni-leipzig.de
[♣] Email: zeckzer@informatik.uni-leipzig.de
[△]These authors contributed equally to this work.

Contents

1 Method **3**
1.1 Goal . . . 3
1.2 Selecting and Preprocessing Genome . . . 3
1.2.1 Method . . . 3
1.2.2 Selection . . . 3
1.2.3 Visualization . . . 4
1.3 Segmentation . . . 4
1.3.1 Method . . . 4
1.3.1.1 Basic statistics . . . 4
1.3.1.2 Length analysis . . . 4
1.3.1.3 Pairs analysis . . . 5
1.3.1.4 Analysis of Segment – Short Segment – Segment Triplets . . . 5
1.3.2 Selection . . . 6
1.3.3 Visualization . . . 6
1.3.3.1 Overview . . . 6
1.3.3.2 Basic statistics . . . 6
1.3.3.3 Length analysis . . . 6
1.3.3.4 Pairs analysis . . . 6
1.3.3.5 Analysis of Segment – Short Segment – Segment Triplets . . . 7
1.4 Additional Measurements . . . 21
1.4.1 Method . . . 21
1.4.2 Selection . . . 21
1.4.3 Visualization . . . 21
1.4.3.1 Overview . . . 21
1.4.3.2 Additional Measurements . . . 21
1.5 Fate-of-Code Analysis . . . 23
1.5.1 Method . . . 23
1.5.2 Selection . . . 23
1.5.3 Visualization . . . 23
1.6 Motif Coverage Analysis . . . 26
1.6.1 Method . . . 26
1.6.2 Selection . . . 26
1.6.3 Visualization . . . 26
1.7 Position Weight Matrix Coverage Analysis . . . 29
1.7.1 Method . . . 29
1.7.2 Selection . . . 29
1.7.3 Visualization . . . 29
1.8 Correlation Analysis . . . 34
1.8.1 Method . . . 34
1.8.2 Selection . . . 34
1.8.3 Visualization . . . 34

2 Technical Details and Graphical User Interface **36**
2.1 System . . . 36
2.2 Masakari Server . . . 36
2.3 Masakari Client . . . 36
2.3.1 Communication with Server . . . 36
2.3.2 Parameters and Starting Computation . . . 37
2.3.3 Loading and saving the configuration . . . 37

2.3.4 Export . . . 37

3 Results: Data, Data Preparation, and Additional Results and Figures **39**
3.1 Cell Line, Cell Types, and Modifications . . . 39
3.2 Data Preparation . . . 39
3.3 Short Segments Chains . . . 42
3.4 Additional Data . . . 45
3.4.1 Modifications . . . 45
3.4.2 CTCF . . . 45
3.4.3 Fate-Of-Code . . . 45

Chapter 1

Method

1.1 Goal

The goal of Masakari is providing an interactive tool for:

Segmentation: Build a segmentation of a genome based on a set of reference measurements (Section 1.3)

Overlap of Additional Measurements: Compute the overlap of additional measurements with the segmentation (Section 1.4)

Compute the "fate of code": Compute a code for a set of additional measurements that relates to the code obtained from the set of reference measurements; the new code is compared to the code of the segment (Section 1.5)

Motif Coverage: Compute the coverage of segments by motifs (Section 1.6)

Position Weight Matrix (PWM) Coverage: Apply position weight matrices to the individual segments to determine their correlation with the different segment types (Section 1.7)

Statistical Correlations: Compute dependencies and correlations between segments, overlap of additional measurements, motifs, and position weight matrices (Section 1.8)

Besides computing these attributes, Masakari provides a graphical user interface that allows to select all relevant data sets—reference genome, reference measurements, additional measurements—as well as the motifs and position weight matrices for which additional information should be computed. Moreover, dashboard visualizations are provided allowing a first analysis of the results of all computations.

The input to Masakari are measurements on the genome in the form of nucleotide ranges for which these measurements are significant. An example would be peak calls resulting from, e.g., Sierra Platinum [15] or other peak callers, that give the ranges of nucleotides (peaks) for specific histone modifications like H3K4me3, H3K27me3, and H3K9me3. We will use the latter example during the subsequent exposition.

1.2 Selecting and Preprocessing Genome

1.2.1 Method

The complete segmentation is computed with respect to a reference genome. This reference genome has to be the same as the one used for obtaining the reference measurements and the additional measurements. Otherwise, the results of the segmentation process may be wrong.

An index is created for the reference genome containing for each chromosome the following information: the number of sequences (chromosomes), the start position of the chromosome in the fasta file, the start position of the sequence (ignoring leading 'N'), the length of the sequence (ignoring tailing 'N'), and the line length. This information is used later to speed up computing the segmentation (Section 1.3), the motif coverage (Section 1.6), and the PWM coverage (Section 1.7). Moreover, this information is saved to an index file for later usage.

If the index exists, it can also be loaded instead of creating it.

1.2.2 Selection

Masakari provides a view that allows selecting a file containing the reference genome (Figure 1.1). Therefore, the user presses the "Select genome" button and uses the filechooser to select the reference genome file. The selected file is then shown in the view.

Next, the user selects an index for the reference genome or decides that an index should be created. For selecting an existing index, the user presses the "Load index" button and selects the respective file in the filechooser. The selected file is then loaded and shown in the view. Otherwise, for creating the index, the user presses the "Create index" button, selects the path, and enters a new filename to hold the index. On closing the selection dialog, the index is created and saved to file, and the filename is shown in the view.

The results of loading the reference genome, and of loading or creating the index are shown in a table. If the reference genome and the index are available, the user can proceed with the next step: creating the segmentation (Section 1.3). Otherwise, the error messages can be analyzed and new files can be chosen or created.

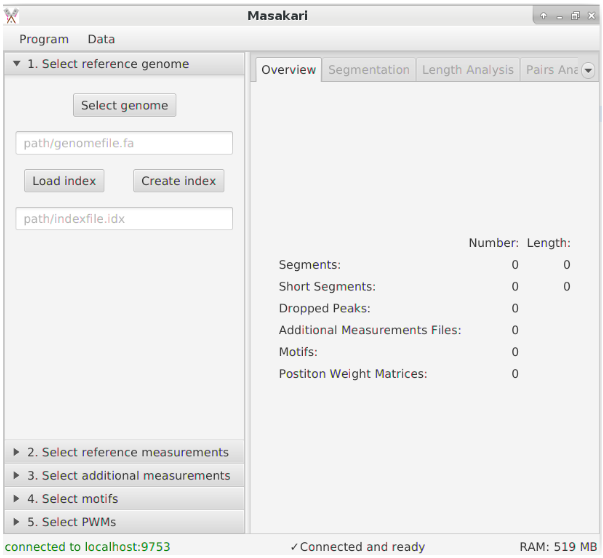

Figure 1.1: Select reference genome: To select the reference genome, the user presses the "Select genome" button and uses the filechooser to select the reference genome file. The selected file is then shown in the view. Further, the user either selects an index for the reference genome or decides that an index should be created. For selecting an existing index, the user presses the "Load index" button and selects the respective file in the filechooser. The selected file is then loaded and shown in the view. Otherwise, for creating the index, the user presses the "Create index" button, selects the path, and enters a new filename to hold the index. On closing the selection dialog, the index is created and saved to file, and the filename is shown in the view. The results of loading the reference genome, and loading or creating the index are shown in a table.

1.2.3 Visualization

The results of this operation do not need a separate visualization. The only important information is whether or not the reference genome and its index were correctly loaded. Both are provided by the selection view (Figure 1.1, left).

1.3 Segmentation

1.3.1 Method

The segmentation is created from at least one reference measurement. Each of the reference measurements is given by the name of the measurement and a set of nucleotide ranges where the signal is significant. We will use the example of histone modifications H3K4me3, H3K27me3, and H3K9me3. The measurements are the peaks obtained by a peak caller. Each nucleotide range is given by such a peak.

Segmenting the genome is then equivalent to building maximal nucleotide ranges where the same combination of zero, one, two, or all three modifications are present. Each segment is represented by its *chromosome*, and its *start* and *end nucleotides* on this chromosome. From these, the *length* of each segment is derived.

As for each segment the coverage of a modification is zero or one, a so-called *code* can be computed for the segment by interpreting the combination of zero's and one's as the binary representation of a number (Figure 1.2, top).

Based on the length, the segments are divided into two groups: ordinary segments (hereafter called *segments*) are segments that exceed a certain minimal length (in our case: 200nt) and *short segments* that are shorter than the minimal length. The amounts of segments and short segments are computed as well as the number of so-called *dropped peaks*. Dropped peaks are those that contribute only to short segments.

After segmentation, a segmentation data table is available containing as many ranges as there are segments (rows). The available information for each segment is its chromosome and range building its identifiers, its code, and its length (columns). Due to the construction of the segmentation data table, the length will always be in the last column. This segmentation data table will be extended with additional information from subsequent steps, if applicable and can be saved for further analyses after all computation steps are completed (Section 2.3.4).

1.3.1.1 Basic statistics

Based on the segmentation, several statistics are computed. First of all, the distribution of codes is computed: for each of the codes, how many segments have this code. Second, the distributions of the lengths of the segments and of the lengths of the short segments are computed. That is, for each (short) segment length it is counted how many (short) segments have this length.

For the short segments two more distributions are computed. The "short segment chain counts" distribution is based on the number of consecutive short segments that is the number of short segments between two segments. For each number of consecutive short segments, its number of occurrence in the segmentation is computed. The "short segment chain length" distribution is based of the number of nucleotides of all short segments between two segments. Again, for each length, its number of occurrence in the segmentation is computed.

1.3.1.2 Length analysis

To enable comparing the length distributions of the reference measurements (e.g., the distribution of width of peaks), to the length distribution of the modified segments, these distributions are computed, too. Moreover, not only the length distribution of the segments but also the length distributions of the modified ($code > 0$) and the unmodified ($code = 0$) segments are computed.

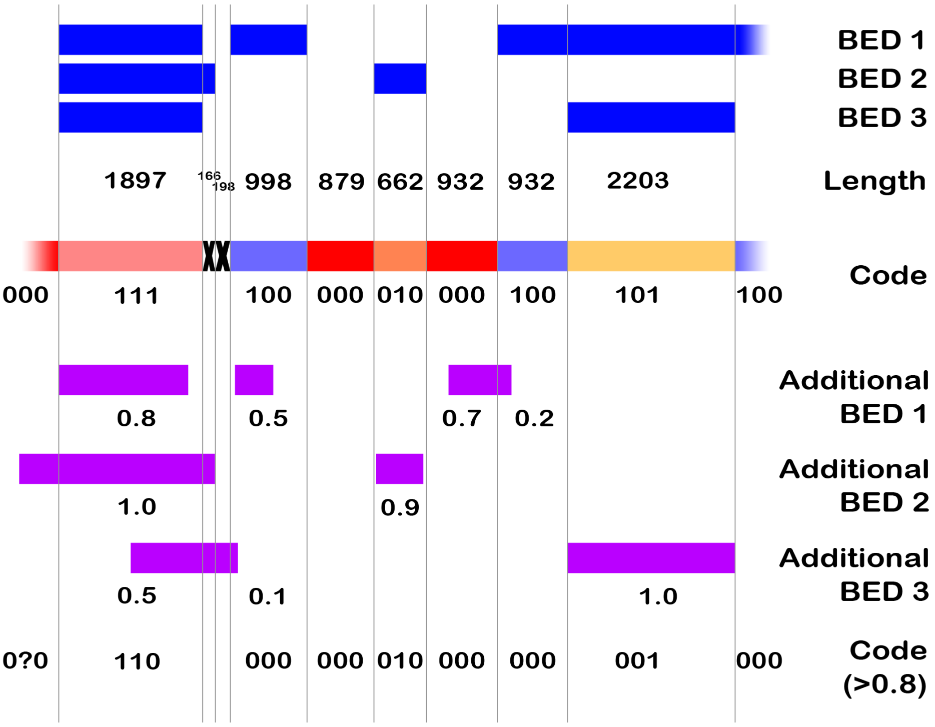

Figure 1.2: Segmentation as well as Additional Measurements Coverage and Code. Top (Segmentation): The peaks are shown in the first three rows (labeled BED 1–BED 3). If a range of nucleotides is not covered by any peak, the resulting code is 000 (middle, two red segments). If a range of nucleotides is covered by one peak, then the code depends on the measurement the peak represents (code 100: blue segment covered by measurement in BED 1, code 010: orange segment covered by measurement in BED 2). The same holds for two measurements covering a range of nucleotides (code 101: yellowish segment covered by measurements of BED 1 and BED 3). Finally, if a range of nucleotides could be covered by all measurements (code 111: pinkish segment). Always the longest sequence of nucleotides is considered for forming a segment. Therefore, two adjacent segments always differ in code. Bottom (Additional Measurements Coverage): For each additional measurement (named Additional BED 1–Additional BED 3, here), the relative amount of modifications is computed resulting in values between 0.0 and 1.0. To compute a code from these relative values, they are compared to a threshold value (here: 0.8). All relative coverages above the threshold value contribute a 1 to the segment code, all others a 0.

1.3.1.3 Pairs analysis

To obtain information about the sequence of nucleotide ranges having different codes, segment-segment, segment-short segment, and short segment-segment pairs are analyzed. The question to be answered by this analysis is: which code follows on which other code how often. For each of these three combinations, two measurements are computed. First of all, a table of first code – second code combinations is computed. The entries in the table denote how often a (short) segment with the first code is followed by a (short) segment with the second code. For example, a code is never followed by itself, as a segment is the longest sequence of nucleotides on a chromosome having the same code.

Second, the Hamming distance between the first and the second code is computed based on their binary representation. From this, a table is created showing how often a (short) segment having a specific code is followed by a (short) segment with a certain Hamming distance. The Hamming distance represents the number of modifications that are present in one but not in the other segment. Again, no second (short) segment will have the same code as the first (short) segment.

Finally, the relative *observed* occurrence of each segment pair (Equation 1.1) is compared to the relative *expected* occurrence of each segment pair (Equation 1.2), where *total* is the total number of segment pairs. Therefore, the relative observed occurrence is divided by the relative expected occurrence (Equation 1.3) yielding the relative occurrence of each pair.

1.3.1.4 Analysis of Segment – Short Segment – Segment Triplets

An analysis of segment – short segment – segment triplets is provided. First of all, all those triples are selected where both segments have the same code. Then, a table is created listing for each segment – short segment code combination, how many of these combinations occur in the data. As for the pairs analysis, the Hamming distance between the binary representations of the segment and the short segment codes is computed. From this, a table is created showing how often

$$occurrence_{relative\_observed} = \frac{occurrence_{observed}}{total} \tag{1.1}$$

$$occurrence_{relative\_expected} = \frac{occurrence_{first\_code} \cdot occurrence_{second\_code}}{2 \cdot total \cdot total} \tag{1.2}$$

$$occurrence_{relative} = \frac{occurrence_{relative\_observed}}{occurrence_{relative\_expected}} \tag{1.3}$$

two segments having a specific code are interrupted by a short segment with a certain Hamming distance. Additionally, the length distribution of the short segments is computed.

1.3.2 Selection

For the segmentation, the files containing the reference measurements and the minimal segment length can be selected (Figure 1.3). For each reference measurement, the file containing it is added by pressing the "add file" button. After choosing the file from the filechooser, it is added to a table.

After adding files, the table holds all measurements chosen. The first column of the table gives the measurement name which is initially set to the file name. The second column provides two action buttons. The first button allows to edit the measurement name. The second button allows to remove the measurement from the selection. Moreover, the order of the measurements can be changed by selecting a measurement and moving it up or down in the table.

Pressing the "start" button starts the computation of the segmentation and its related information.

1.3.3 Visualization

1.3.3.1 Overview

The overview provides the number of segments and the sum of the lengths of all segments as well as the number of short segments and the sum of their lengths (Figure 1.3). Moreover, the number of dropped peaks is provided.

1.3.3.2 Basic statistics

The basic statistics provided are:

- The distribution of codes showing how many segments have a certain code (Figure 1.4).
- The distribution of segment lengths showing how many (short) segments have a certain length (Figure 1.5).
- The distribution of short segment chains counts showing how often consecutive short segments occur between two segments (Figure 1.6).
- The distribution of short segment chains lengths shows how many short segment chains have a certain length (Figure 1.7).

1.3.3.3 Length analysis

For length analysis, we provide two types of charts:

- A histogram for comparing the length distribution of a reference measurement to the length distribution of the segmentation (without unmodified segments, i.e, segments with code 0):
  - Both length bins and number of occurrence linearly scaled (Figure 1.8).
  - Length bins linearly scaled and number of occurrence logarithmically scaled (Figure 1.9).
  - Length bins logarithmically scaled and number of occurrence linearly scaled (Figure 1.10).
  - Both length bins and number of occurrence logarithmically scaled (Figure 1.11).
- A histogram for comparing the length distribution of the modified segments ($code > 0$) to the length distribution of the unmodified segments ($code = 0$) (Figure 1.12).

1.3.3.4 Pairs analysis

The pairs analysis provides three charts for each of the combinations segment – segment, segment – short segment, and short segment – segment:

- A heatmap showing the relation between the code of a (short) segment and the code of the next (short) segment (Figure 1.13).
- A heatmap showing the relation between the code of a (short) segment and the distance to the code of the subsequent (short) segment (Figure 1.14).
- A histogram showing the distribution of the second (short) segment distances for all codes or for a specific code of the first (short) segment (Figure 1.15).

Additionally, a heatmap showing the relative occurrence of each segment pair (log-transformed, scaled by maximum) is provided. Red signifies a higher occurrence than expected, blue a lower one. Saturation shows, how much higher or lower the occurrence is compared to its expected value (Figure 1.16).

1.3.3.5 Analysis of Segment – Short Segment – Segment Triplets

The first three charts provided are the same as for the pairs analysis. Figure 1.17 shows the fourth visualization. Here, the length distribution of all short segments interrupting two segments with the same code is shown.

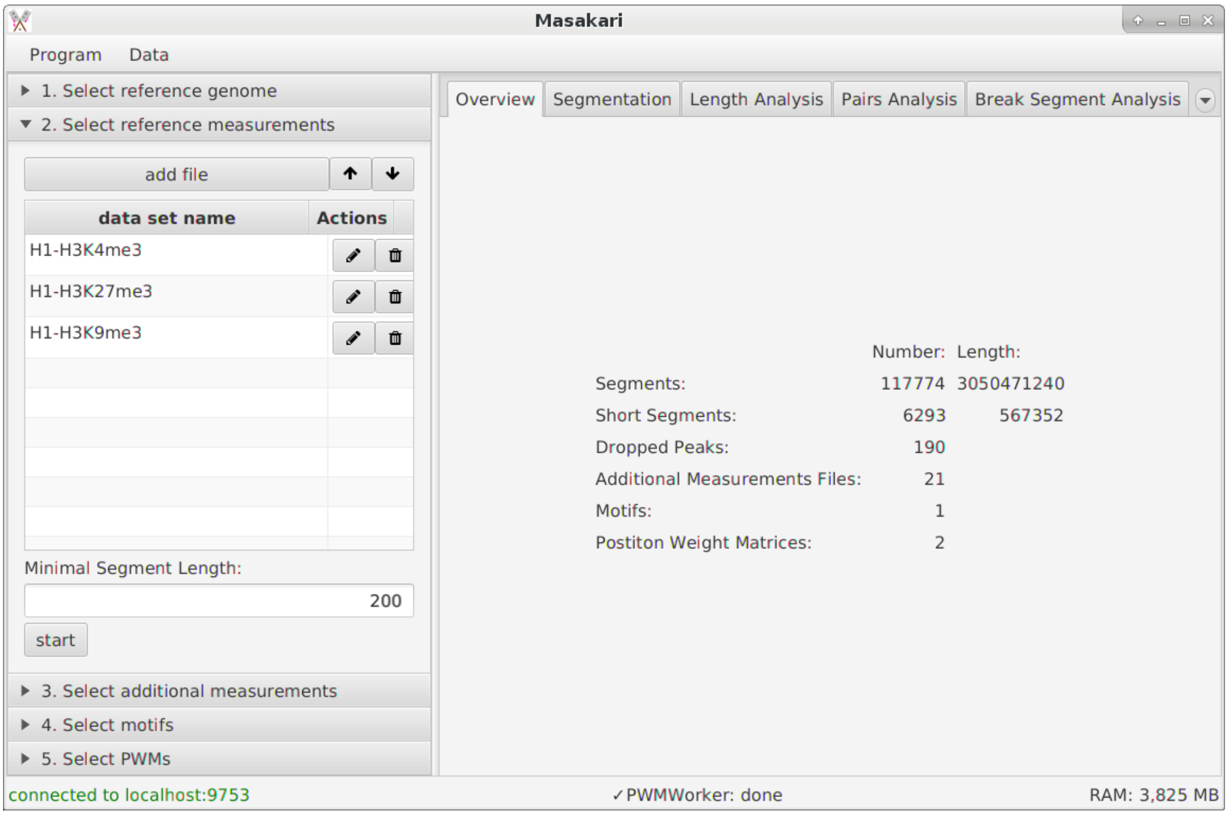

Figure 1.3: Overview: The number of segments and the sum of the lengths of all segments as well as the number of short segments and the sum of their lengths are provided. Moreover, the number of dropped peaks is shown. Finally, the amount of selected additional measurements, motifs, and position weight matrices is reported.

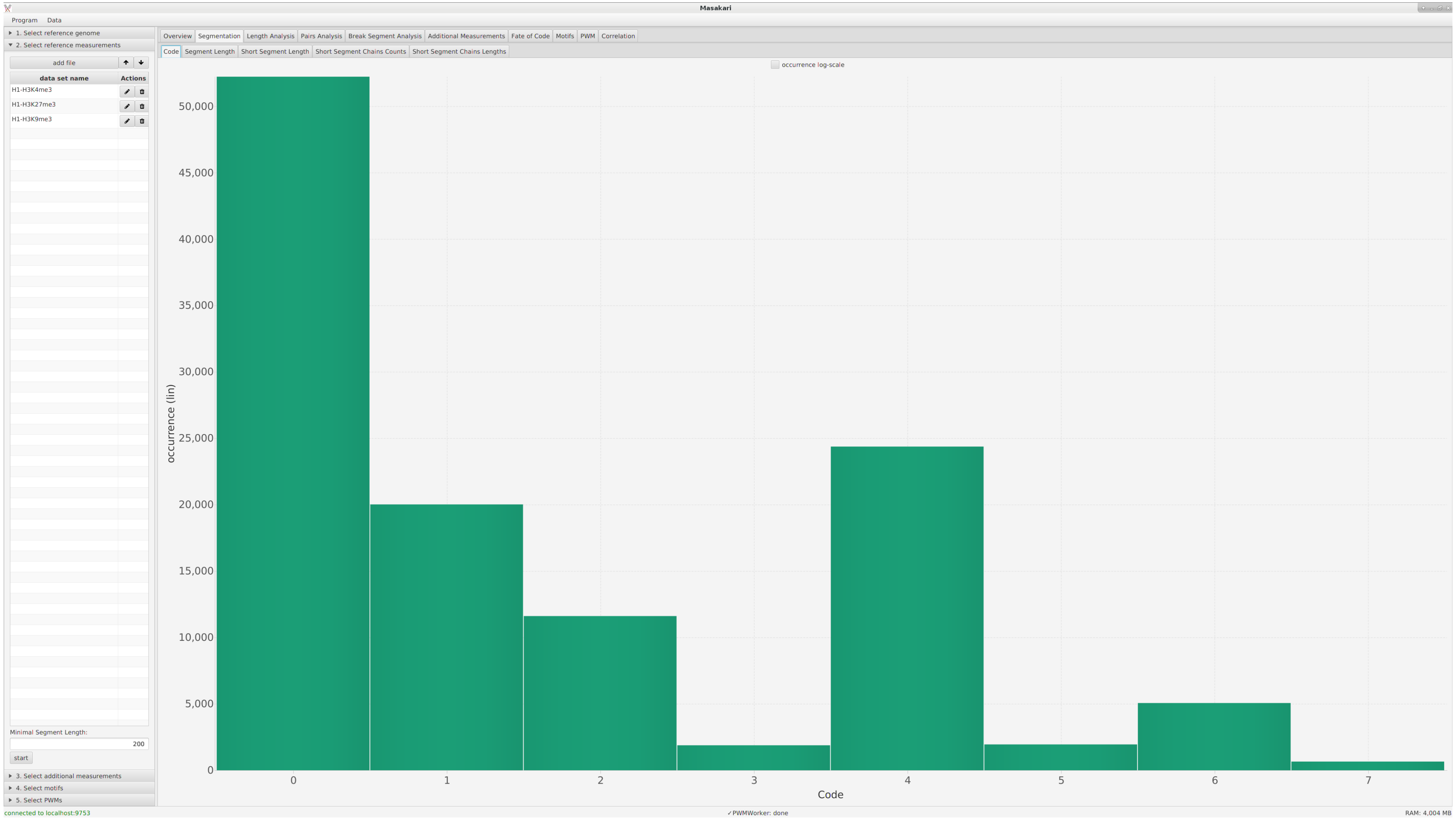

Figure 1.4: Segmentation – Code: The distribution of codes shows how many segments have a certain code. As the differences can be large, a logarithmic scale for the occurrence can be chosen ("occurrence log-scale"). Here, more than 50,000 segments have code 0, while only few segments have code 3, 5, or 7. Choosing the logarithmic scale for the y-axis shows that less than 1,000 segments have code 7 and thus overlapping peaks for all histone modifications.

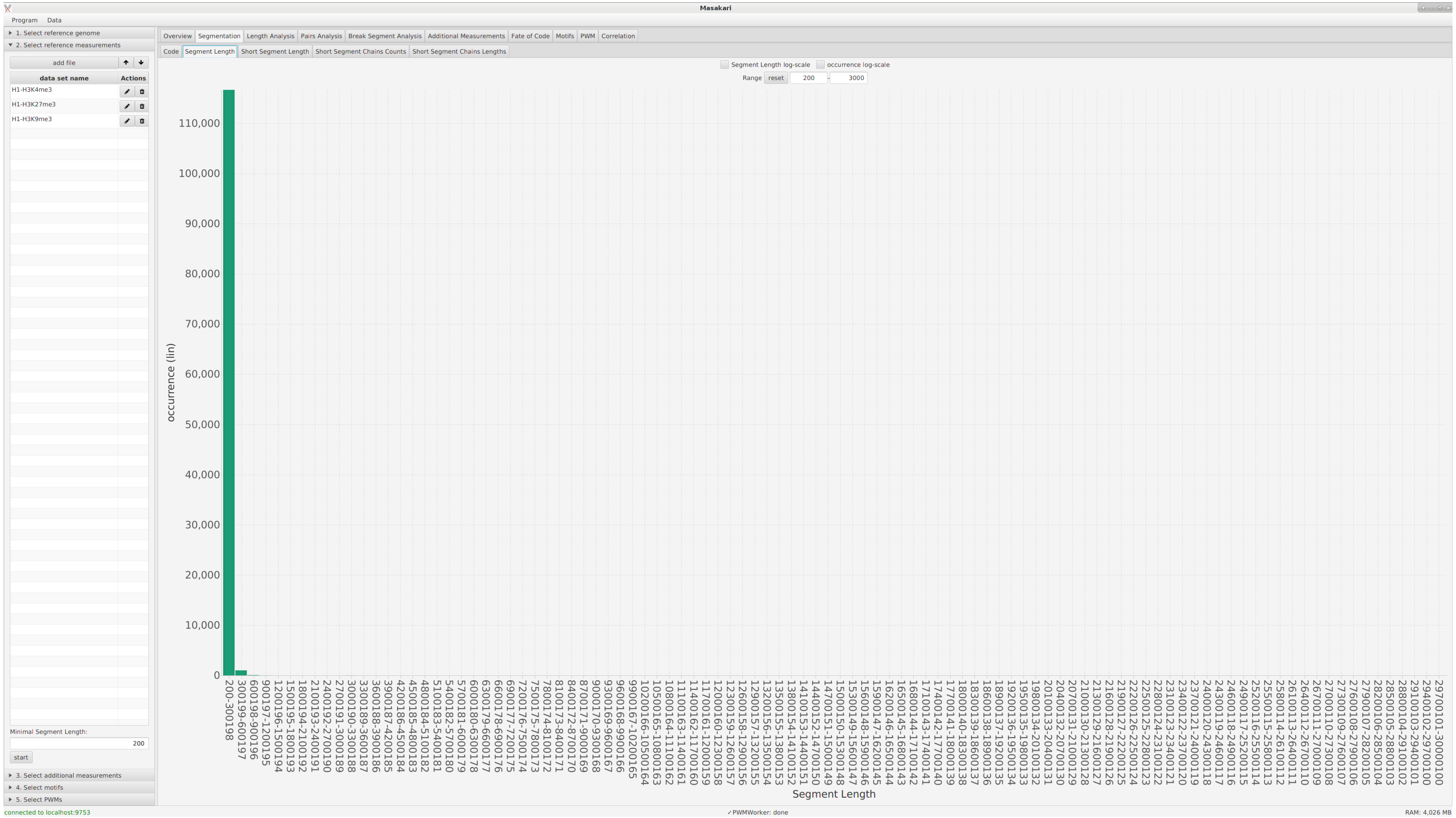

Figure 1.5: Segmentation – Segment Length & Segmentation – Short Segment Length: The distribution of segment lengths shows how many segments have a certain length. As the number of different lengths can be large, binning is applied. Moreover, for both length (x-axis) and occurrence (y-axis), using a logarithmic scale can be chosen. ("Segment Length log-scale" and "occurrence log-scale", respectively). In case of logarithmic scaling of lengths (x-axis), the binning is adapted accordingly. To get more details, a subrange of the lengths can be selected. The "reset" button selects the complete range. Double clicking a bar selects the range of this bar.

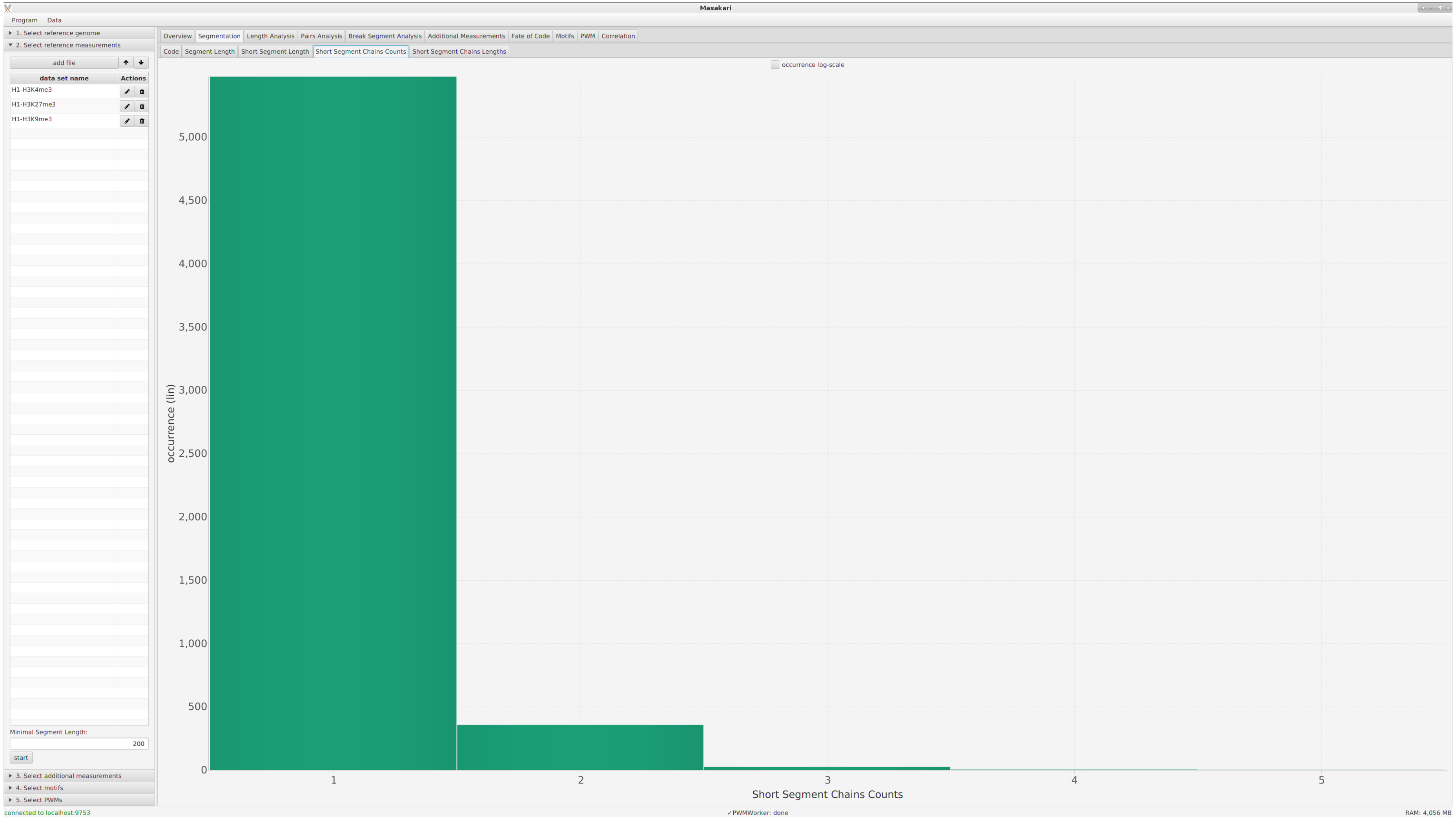

Figure 1.6: Segmentation – Short Segment Chains Counts: The distribution of short segment chains counts shows how often consecutive short segments occur between two segments. The x-axis gives the number of consecutive short segments while the y-axis gives the number of occurrence of each such count. As the number of count occurrences can differ by order of magnitudes, it can be scaled logarithmically ("occurrence log-scale").

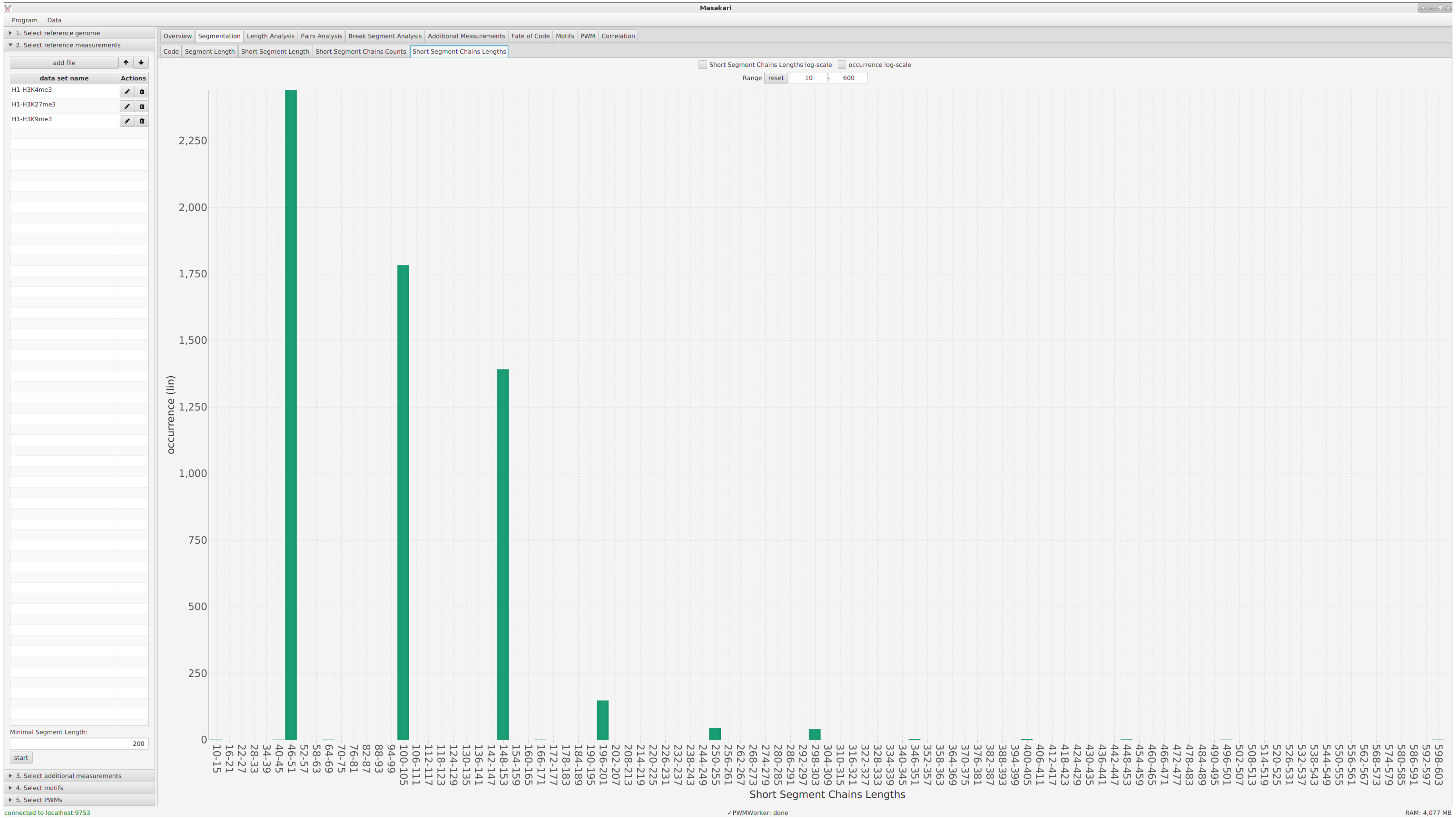

Figure 1.7: Segmentation – Short Segment Chains Lengths: The distribution of short segment chain lengths shows how many short segment chains have a certain length. Binning is applied if the number of possible lengths is above a certain threshold (currently, 100nt). Moreover, for both lengths (x-axis) and occurrence (y-axis), using a logarithmic scale can be chosen ("Short Segment Chains Lengths log-scale" and "occurrence log-scale", respectively). In case of logarithmic scaling of lengths (x-axis), the binning is adapted accordingly. To get more details, a subrange of the lengths can be selected. Double clicking a bar selects the range of this bar. The "reset" button selects the complete range. Here, most short segment lengths are multiples of 50 which stems from the offset during peak-calling.

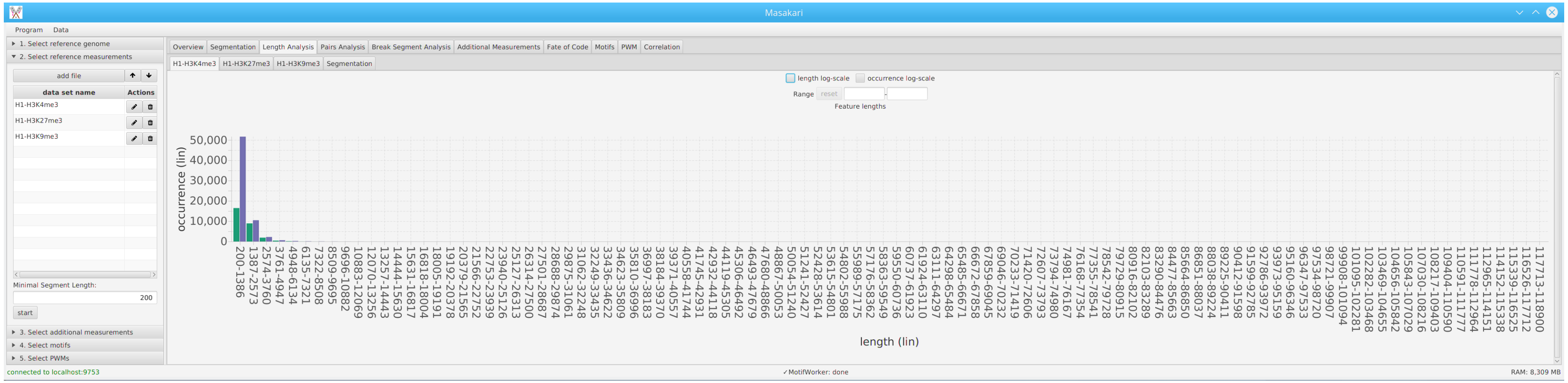

Figure 1.8: Length Analysis – Reference Measurement: The histogram supports comparing a reference measurement (here: H3K4me3, purple bars) to the segmentation (green bars) with respect to their length distributions. Binning is applied for the lengths. Moreover, for both length (x-axis) and occurrence (y-axis), using a logarithmic scale can be chosen ("length log-scale" and "occurrence log-scale", respectively). In this figure, both length bins and number of occurrence are linearly scaled. To get more details, a subrange of the lengths can be selected. Double clicking a bar selects the range of this bar. The "reset" button selects the complete range.

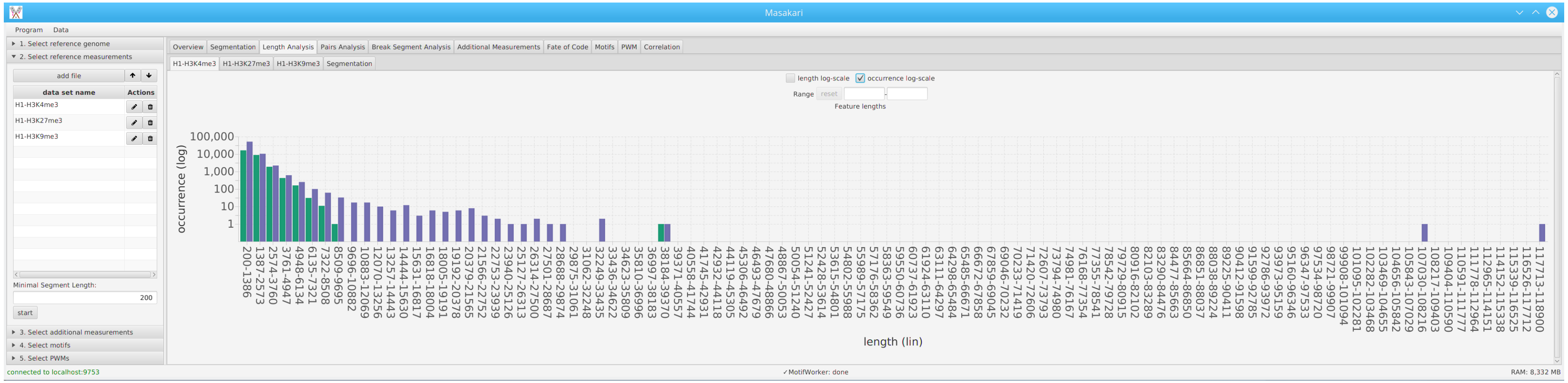

Figure 1.9: Length Analysis – Reference Measurement: The histogram supports comparing a reference measurement (here: H3K4me3, purple bars) to the segmentation (green bars) with respect to their length distributions. Binning is applied for the lengths. Moreover, for both length (x-axis) and occurrence (y-axis), using a logarithmic scale can be chosen ("length log-scale" and "occurrence log-scale", respectively). In this figure, length bins are linearly scaled and number of occurrence is logarithmically scaled. To get more details, a subrange of the lengths can be selected. Double clicking a bar selects the range of this bar. The "reset" button selects the complete range.

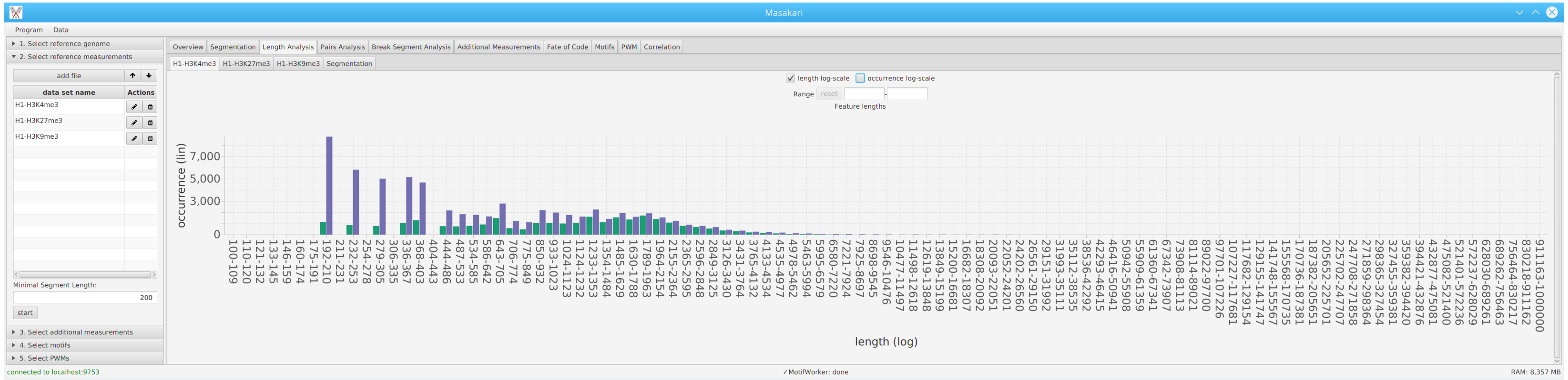

Figure 1.10: Length Analysis – Reference Measurement: The histogram supports comparing a reference measurement (here: H3K4me3, purple bars) to the segmentation (green bars) with respect to their length distributions. Binning is applied for the lengths. Moreover, for both length (x-axis) and occurrence (y-axis), using a logarithmic scale can be chosen (“length log-scale” and “occurrence log-scale”, respectively). In this figure, length bins are logarithmically scaled and number of occurrence is linearly scaled. To get more details, a subrange of the lengths can be selected. Double clicking a bar selects the range of this bar. The “reset” button selects the complete range.

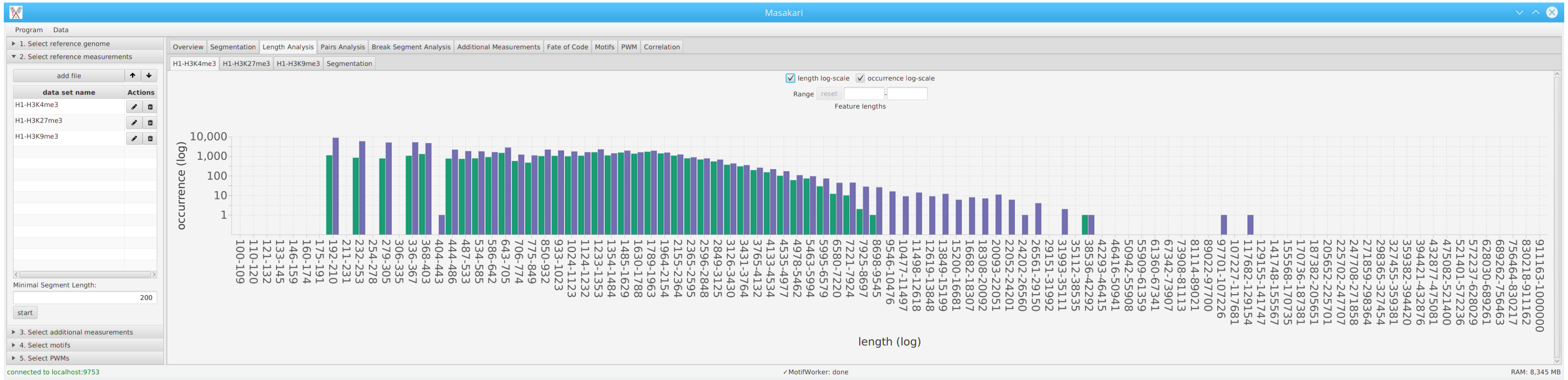

Figure 1.11: Length Analysis – Reference Measurement: The histogram supports comparing a reference measurement (here: H3K4me3, purple bars) to the segmentation (green bars) with respect to their length distributions. Binning is applied for the lengths. Moreover, for both length (x-axis) and occurrence (y-axis), using a logarithmic scale can be chosen (“length log-scale” and “occurrence log-scale”, respectively). In this figure, both length bins and number of occurrence are logarithmically scaled. To get more details, a subrange of the lengths can be selected. Double clicking a bar selects the range of this bar. The “reset” button selects the complete range.

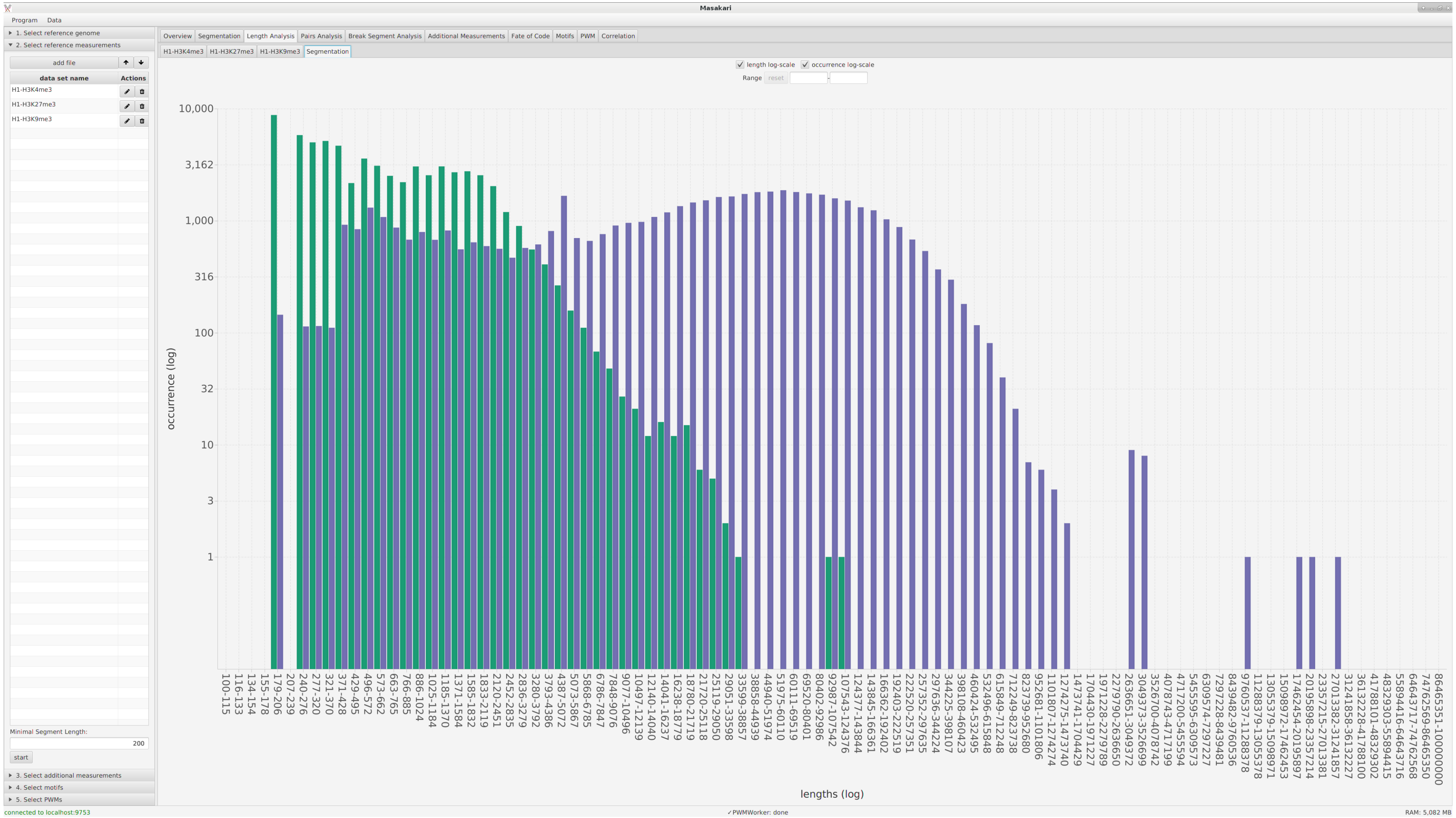

Figure 1.12: Length Analysis – Reference Measurement: The histogram supports comparing the modified segments (green bars) to the unmodified segments (purple bars) with respect to their length distributions. Binning is applied for the lengths. Moreover, for both length (x-axis) and occurrence (y-axis), using a logarithmic scale can be chosen ("length log-scale" and "occurrence log-scale", respectively). In this figure, both length bins and number of occurrence are logarithmically scaled. To get more details, a subrange of the lengths can be selected. Double clicking a bar selects the range of this bar. The "reset" button selects the complete range.

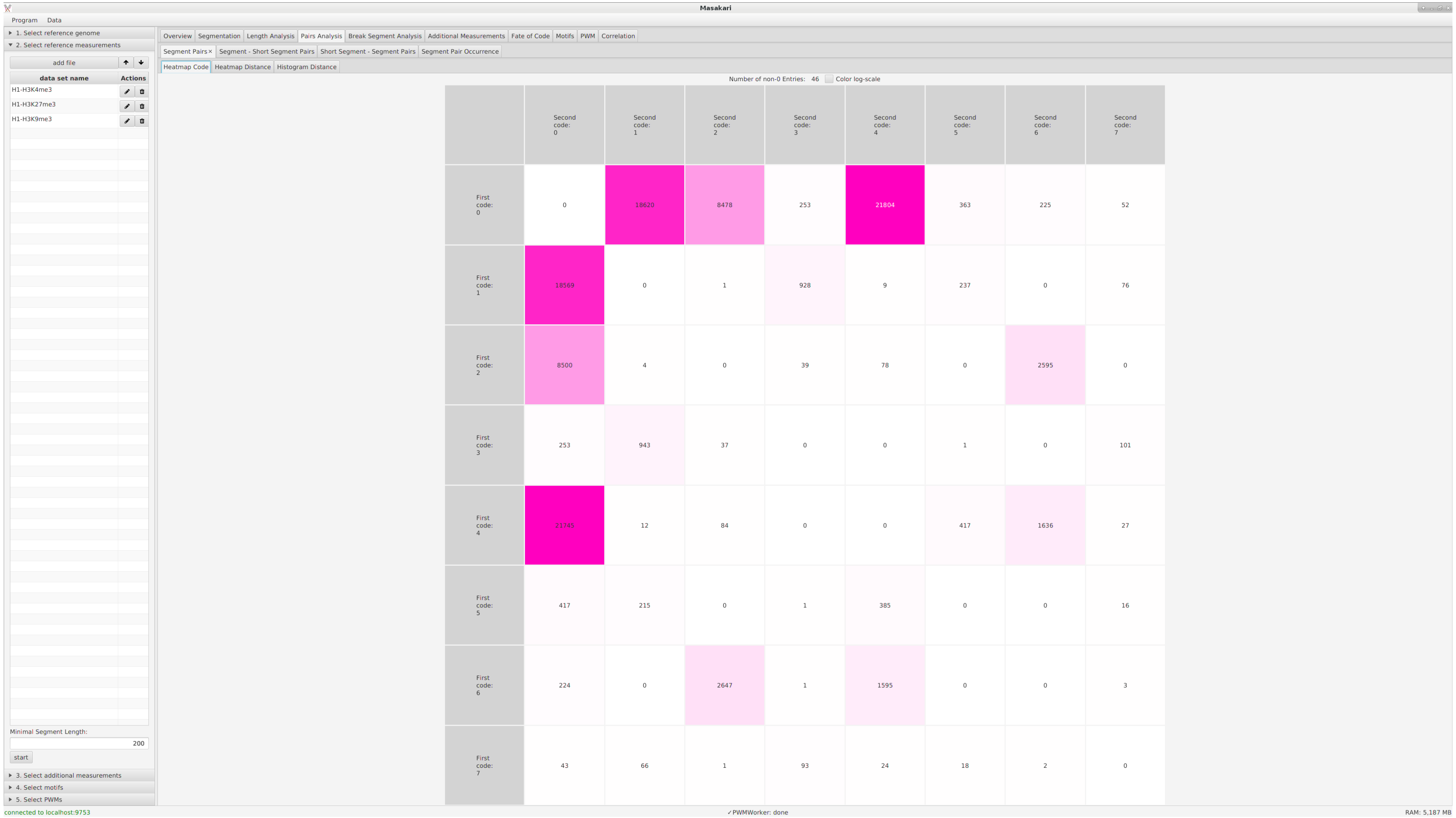

Figure 1.13: Pairs Analysis – Heatmap Code: A heatmap is used to show the relation between the code of a (short) segment and the code of the next (short) segment. These codes are denoted as “first code” and “second code”, respectively. The number of combinations found is given as “Number of non-0 entries”; in this case 47. As the maximal number of combinations is $8 \cdot 8 - 8 = 56$, 9 combinations do not occur in the data. The saturation of the cell shows, how many (short) segment pairs have a certain combination of codes in relation to the maximum. The color saturation can be scaled logarithmically (“Color log-scale”).

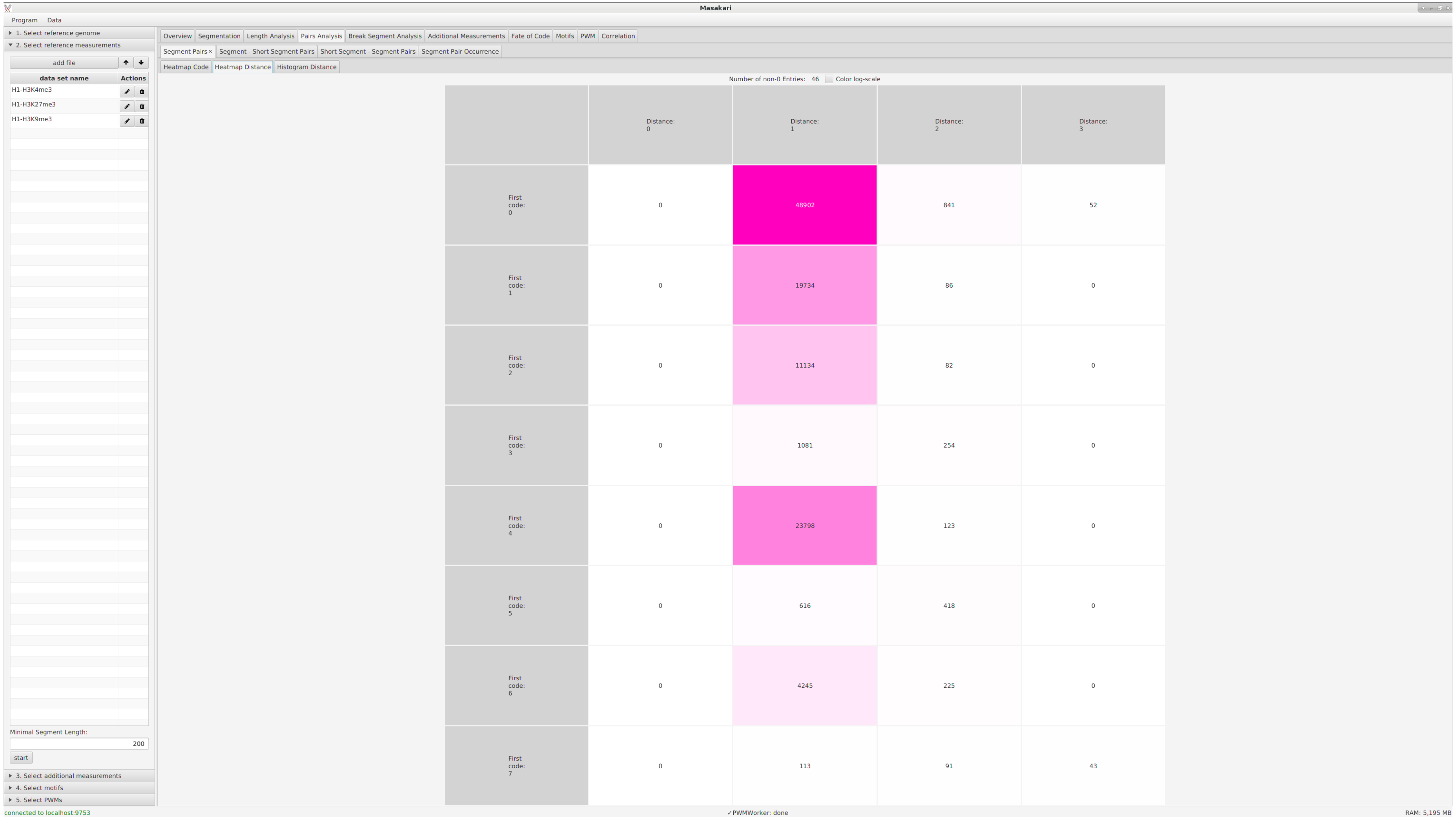

Figure 1.14: Pairs analysis – Heatmap Distance: A heatmap is used to show the relation between code of the first (short) segment and the distance to the code of the subsequent (short) segment. The number of combinations found is given as “Number of non-0 entries”; in this case 47. The saturation of the cell shows, how many second (short) segments have a code with the respective distance to the code of the first (short) segment. The color saturation can be scaled logarithmically (“Color log-scale”).

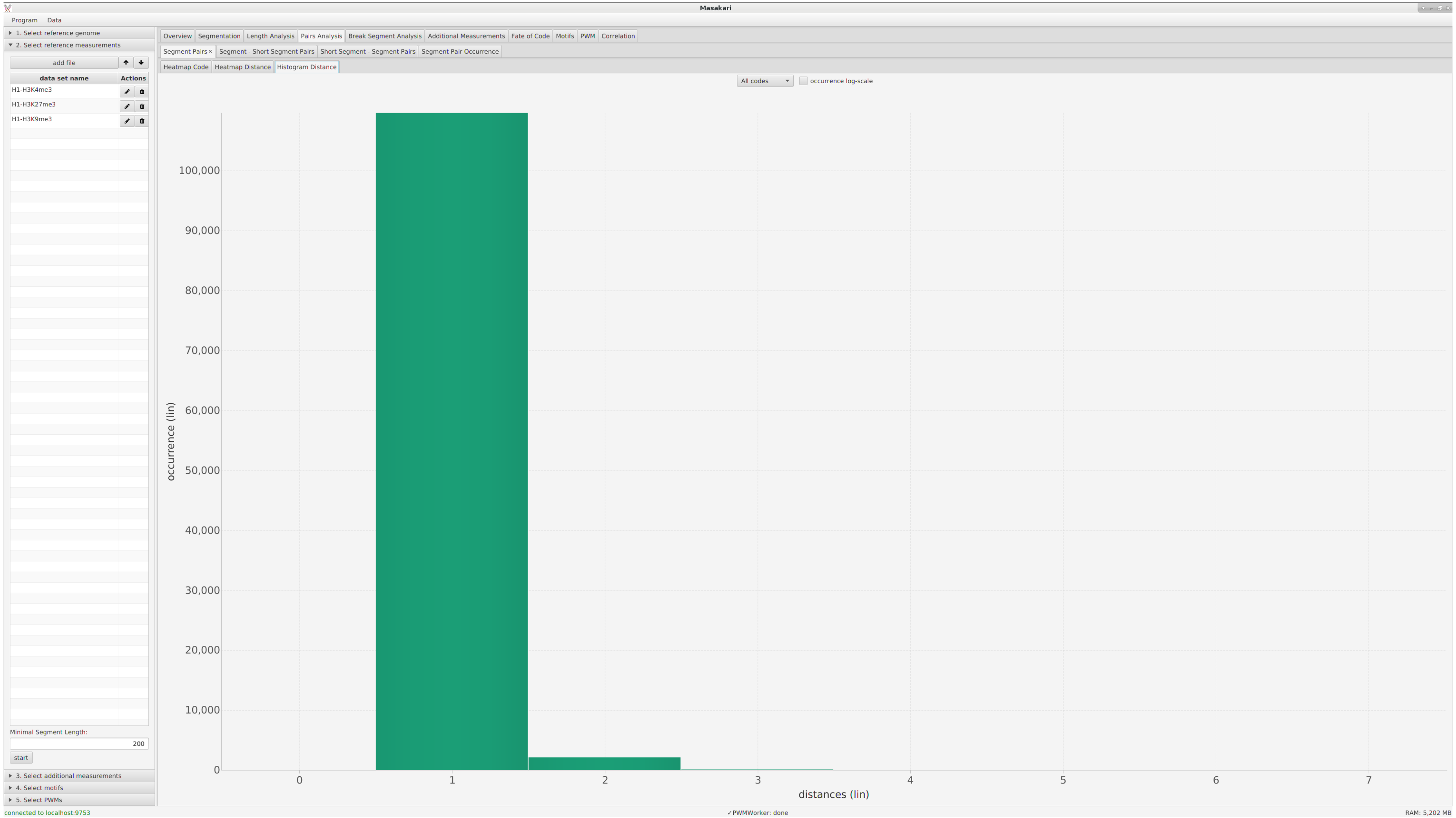

Figure 1.15: Pairs analysis – Histogram Distance: The histogram shows the distribution of second (short) segment distances for all codes of the first (short) segment. The number of these distances can be logarithmically scaled ("occurrence log-scale"). Moreover, the histogram can also be used to show the distribution of second (short) segment distances for individual codes of the first (short) segment. For this, the respective code can be chosen from the drop down box at the top. Here, most segments have a distance of 1, which means that they differ by at most one modification.

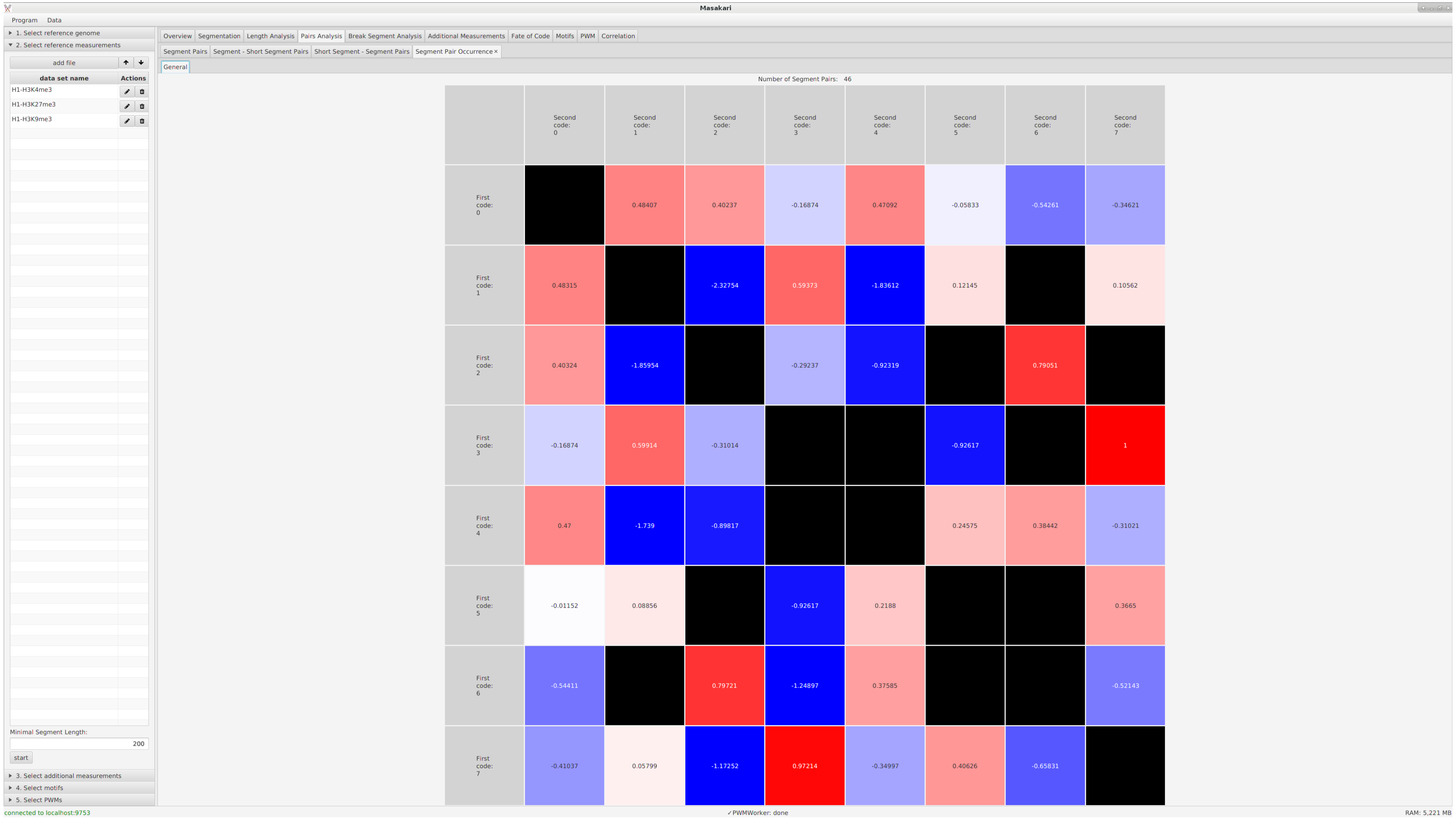

Figure 1.16: Pairs analysis – Segment Pair Occurrence: The heatmap shows the relative occurrence of each segment pair (log-transformed, scaled by maximum). Red signifies a higher occurrence than expected, blue a lower one. Saturation shows, how much higher or lower the occurrence is compared to its expected value.

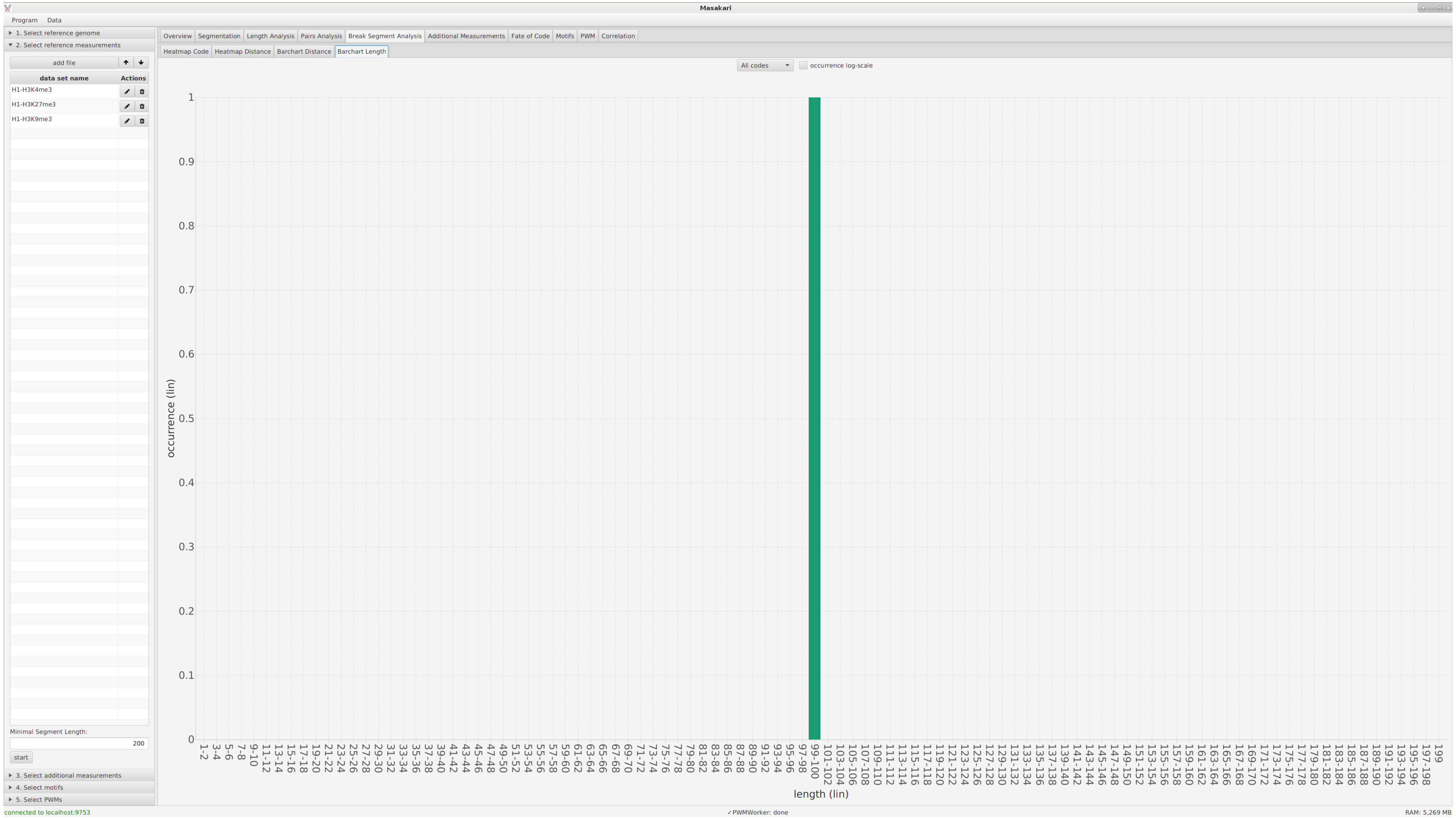

Figure 1.17: Segment – Short Segment – Segment Triplets Analysis – Histogram Length: The histogram shows the length distribution of all short segments interrupting two segments with the same code. The number of occurrence for each length can be scaled logarithmically (“occurrence log-scale”). Moreover, the length distribution can be shown for short segments with a specific code only. For this, the respective code can be chosen from the drop down box at the top. Here, only one such triplet exists, which implies a valuable segmentation regarding this property.

1.4 Additional Measurements

1.4.1 Method

Having a segmentation based on reference measurements, additional measurements are compared to the segments obtained. Thereby, the nucleotide ranges of each additional measurement are compared to the segment ranges (Figure 1.2, bottom). If the nucleotide range of a segment is completely part of a range of the additional measurement then the coverage of this segment is 100% = 1.0. If the nucleotide range of a segment does not overlap with any range of the additional measurement then the coverage of this segment is 0% = 0.0. In all other cases, the coverage of the segment by the additional measurement is determined by the number of nucleotides of the segment overlapping the additional measurement divided by the number of nucleotides of the segment. Thus, the resulting coverage is always in the interval $[0; 1]$.

After computing the coverage of the segments by the additional measurements, the segmentation data table is extended by the obtained coverage value (row) for each of the measurements. Thus, adding three additional measurements leads to three more rows.

1.4.2 Selection

As for the segmentation, the files containing the additional measurements can be selected (Figure 1.3). For each additional measurement, the file containing it is added by pressing the “add file” button. After choosing the file from the filechooser, it is added to a table.

After adding all files, the table holds all the measurements chosen. The first column of the table gives the measurement name which is initially set to the file name. The second column provides two action buttons. The first button allows to edit the measurement name. The second button allows to remove the measurement from the selection. Moreover, the order of the measurements can be changed by selecting a measurement and moving it up or down in the table.

Pressing the “start” button starts the computation of the coverage of each segment by the additional data.

1.4.3 Visualization

1.4.3.1 Overview

The overview provides the number of additional measurements (Figure 1.3).

1.4.3.2 Additional Measurements

Figure 1.18 shows the histogram provided for coverage analysis.

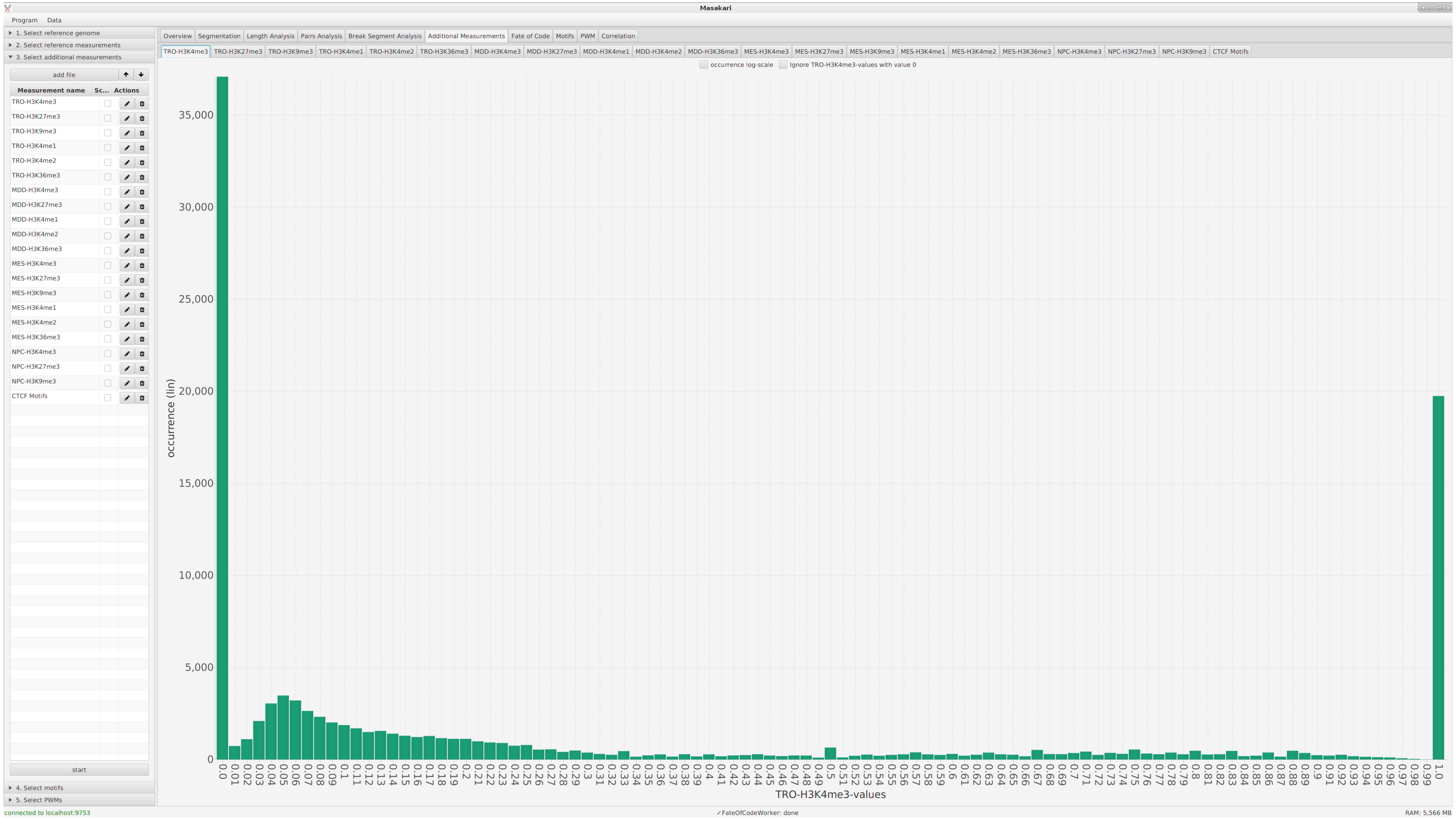

Figure 1.18: Additional Measurements – Coverage: For each additional measurement, a histogram describing the coverage of segments by the additional measurement is shown. For example, the H3K4me3 modification of a different cell type can be compared to the reference cell type. The different coverage values are binned using 101 bins and for each bin, the number of segments (occurrence) having this coverage value is shown in the histogram. The occurrence can be displayed using a logarithmic scale ("occurrence log-scale"). Moreover, the segments with 0 coverage can be ignored ("Ignore ... having value 0"). This is useful as for some additional data, the number of not-covered segments is dominant by an order of magnitude.

1.5 Fate-of-Code Analysis

1.5.1 Method

The fate-of-code analysis combines the segmentation of the reference measurements and the coverage of the segments by the additional data measurements. The precondition for this analysis is that a set of additional measurements matches the set of reference measurements. One example is comparing the same histone modifications in different cell types. If the reference measurements are given by the histone modifications H3K4me3, K3K27me3, and H3K9me3 for one cell type, then measuring the same modifications on another cell type leads to three additional measurement data sets (Figure 1.2, bottom). Keeping the same order, for each segment a code is computed from the set of additional measurements based on a threshold (here: 0.8). This code is then compared to the code of the segment (obtained from the reference measurements) and the result is a table of how many segments having one code in the reference data set have a certain code (same or other) in the additional data set.

No additional information for the resulting segmentation data table is computed.

1.5.2 Selection

In the setup (Figure 1.19), for each set of reference measurements the corresponding set of additional measurements is chosen from a drop down box. By default, one set of additional measurements can be chosen. If appropriate—several sets of measurements, e.g., for multiple cell types, were added in step 3—further sets of additional measurements can be added ("Add Additional Data" button).

Pressing the "Reset" button, removes all selections. On pressing "Submit" the fate-of-code computation is started and the "Result" part is opened.

1.5.3 Visualization

The visualization consists of two parts. The first part supports setting up the fate-of-code computation by providing the necessary interaction facilities (Figure 1.19). The second part supports analyzing the results by showing the table as a heatmap (Figure 1.20). If several sets of additional data measurements were selected, a table is shown for each of them without repeating the row headers.

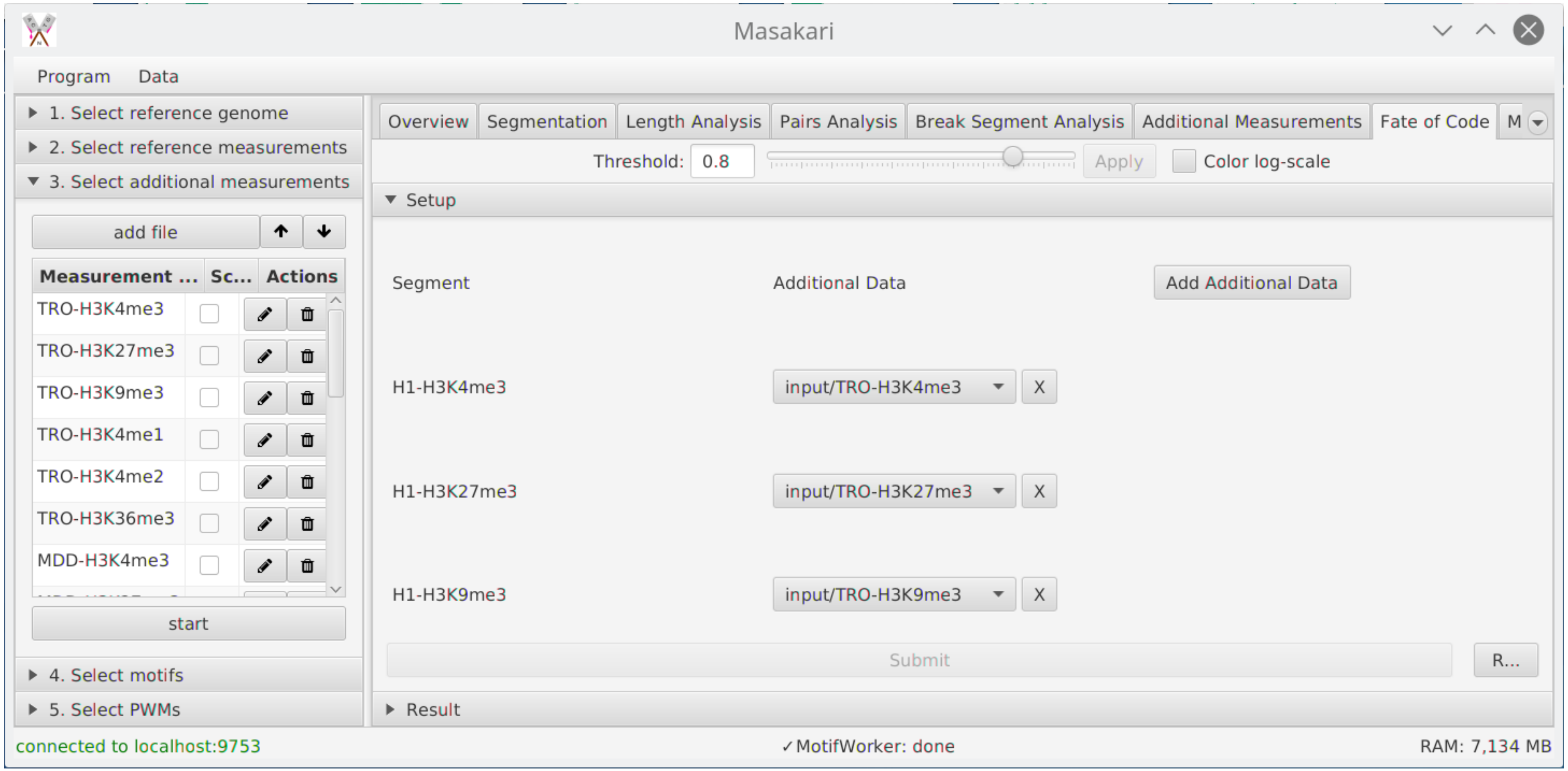

Figure 1.19: Fate of Code – Setup: In the setup view, the additional measurements can be associated with the reference measurements for each additional code to be computed. The threshold for assigning a "1" can be set. If appropriate—several sets of measurements, e.g., for multiple cell types, were added in step 3—further sets of additional measurements can be added ("Add Additional Data" button). Pressing the "Reset" button, removes all selections. On pressing "Submit" the fate-of-code computation is started and the "Result" part is opened.

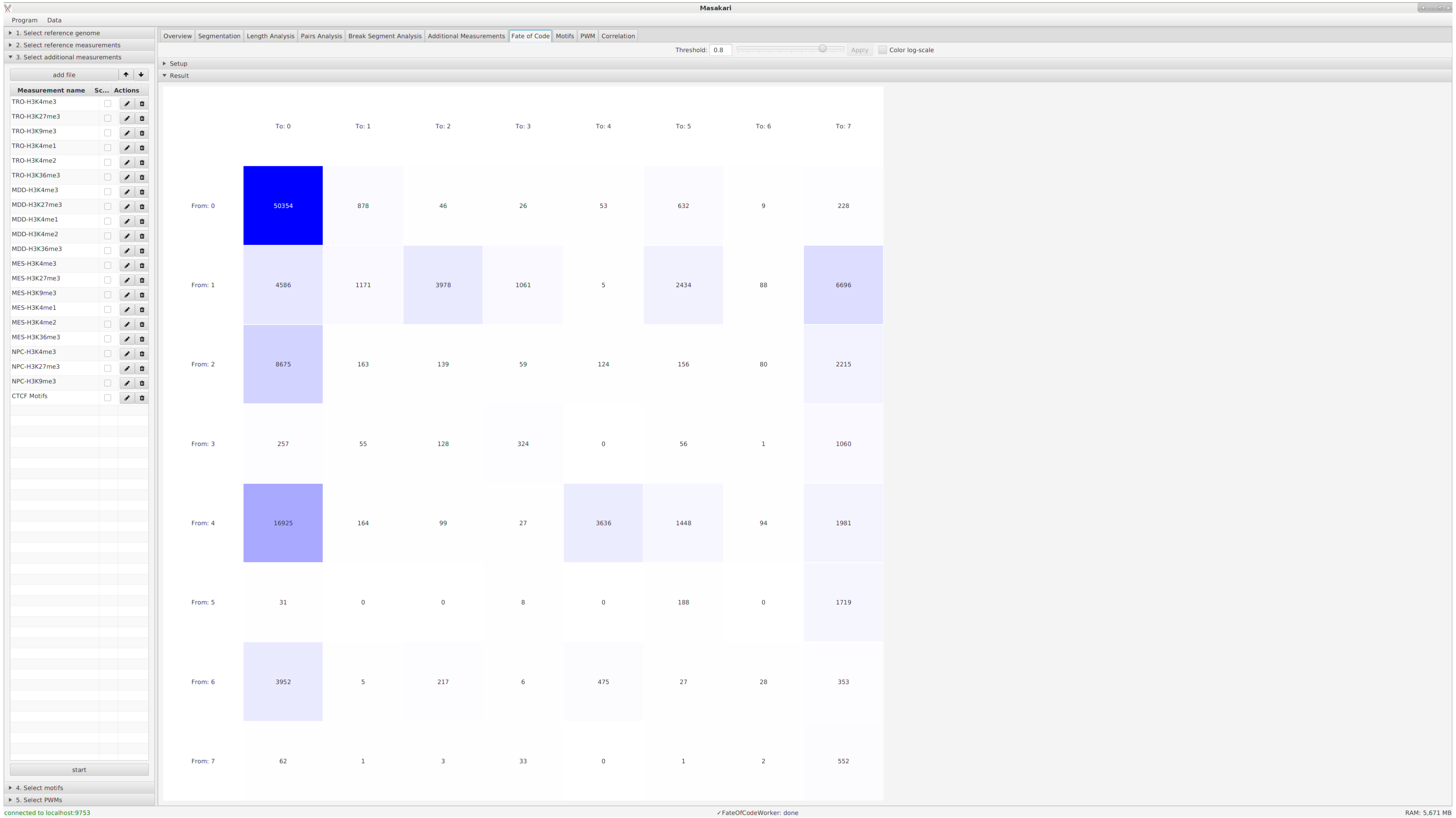

Figure 1.20: Fate of Code – Result: The results of the fate-of-code computation are shown using a heatmap. More saturated colors designate that more segments have a transition from the reference code (From) to the additional data code (To). The color can be scaled logarithmically ("Color log-scale"). Changing the threshold leads to a recomputation of the fate-of-code followed by an update of the heatmap.

1.6 Motif Coverage Analysis

1.6.1 Method

To compute the motif coverage, the search structure on the reference genome generated while loading it is used. For each segment, its nucleotide sequence is retrieved from the reference genome. Next, the motifs to search for are prepared. As the genomic sequence stored contains only one strand of the double-stranded DNA, the reverse complement of the motif is created. Further, for both the original motif and its reverse complement an uppercase and a lowercase version are created. Then, these four derived character sequences are used to search for motif occurrences on the nucleotide sequence of the segment. Either the number of occurrences (count) or its normalized value (dividing the count by the number of possible occurrences) is stored for each segment.

This operation is quite time consuming due to reading the nucleotide sequences of the segments from external storage (because of the size of the reference genome, it is not stored in internal memory). Therefore, the search is not performed one motif after another. Instead, the nucleotide sequence of each segment is loaded exactly once and then all selected motifs are searched for. Moreover, searching for motifs is done in parallel depending on the available resources (CPUs and threads) of the hardware used.

After computing the coverage of the segments by the motifs, the segmentation data table is extended by the obtained coverage value (row) for each of the motifs.

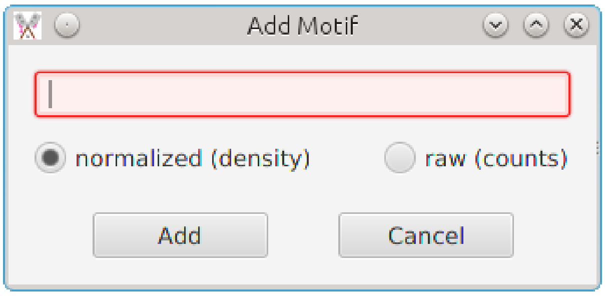

Figure 1.21: Add Motif dialog: It shows a text field for entering the motif. Two radio buttons can be used to select whether the density (normalized values) or the count (raw values) should be computed. Pressing the "Add" button adds the motif to the table and closes the dialog. Pressing the "Cancel" button ends the dialog without adding the motif.

1.6.2 Selection

The motifs are added one by one (Figure 1.22). The process is similar to selecting a reference or an additional measurement. After pressing the "add motif" button, a dialog is opened (Figure 1.21). It shows a text field for entering the motif. Two radio buttons can be used to selected whether the density (normalized values) or the count (raw values) should be computed. Pressing the "Add" button adds the motif to the table and closes the dialog. Pressing the "Cancel" button ends the dialog without adding the motif.

A table (Figure 1.22) shows for each selected motif its nucleotide sequence, whether or not to normalize it (checkbox), and an action button allowing to remove the motif. The order of the motifs can be changed using the move-up and move-down buttons. Pressing the "start" button starts the computation of the motif coverage.

1.6.3 Visualization

The results of the motif coverage computation are shown using histograms (Figures 1.22 and 1.23).

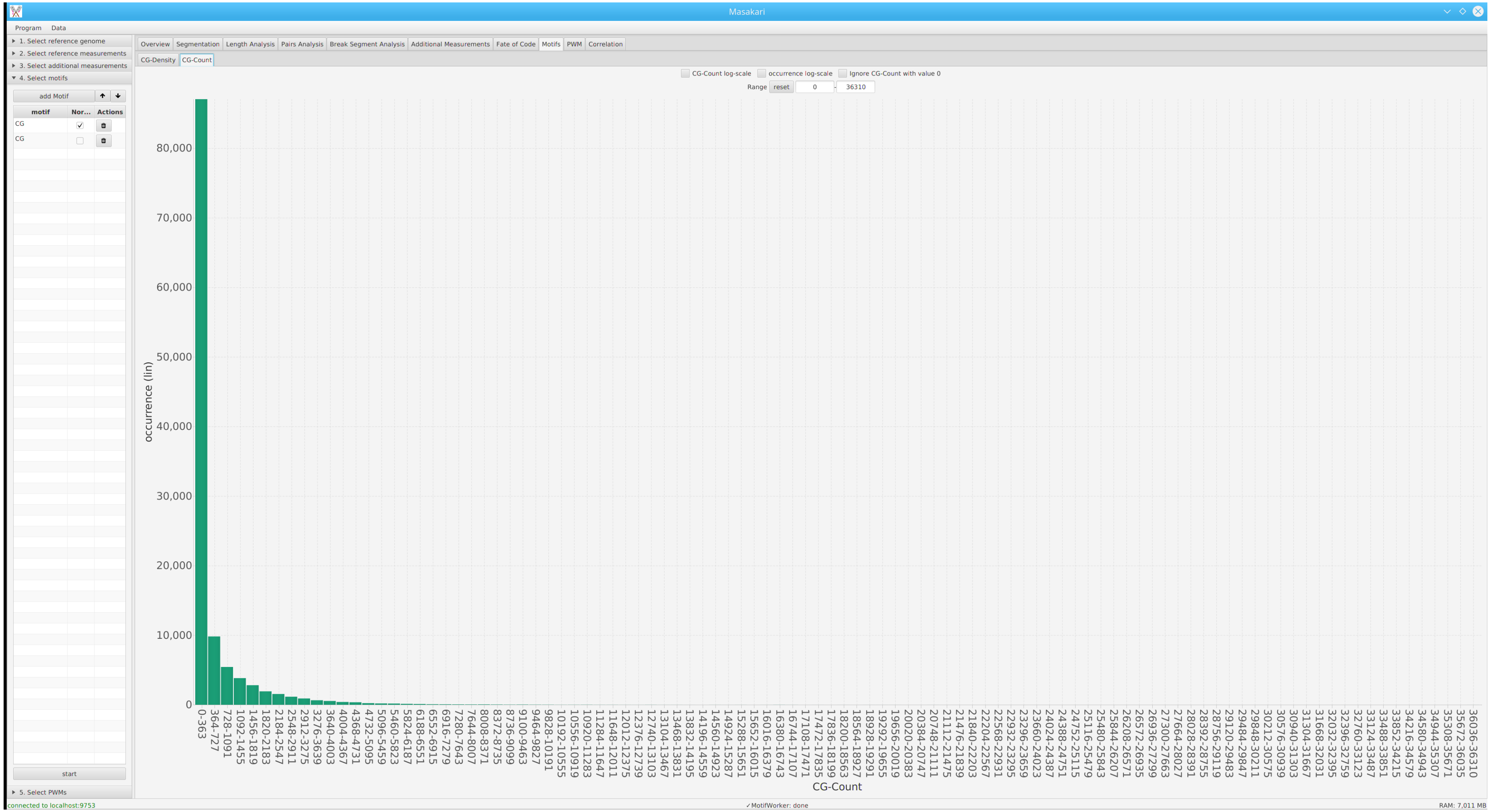

Figure 1.22: Motifs – CG-Count: This histogram shows the results of counting the CG occurrences in the segments. The counts are binned and the number of segments having counts in a specific bin is shown by a bar. Both the counts and the number of occurrences can be scaled logarithmically ("CG-Count log-scale" and "occurrence log-scale", respectively). As segments without any motif occurrence might be dominant, these can be removed from the result ("ignore CG-Count with value 0"). The range of counts can be chosen by entering it into the number fields or by selecting a bar (double-click). Pressing the "reset" button will use the original range again.

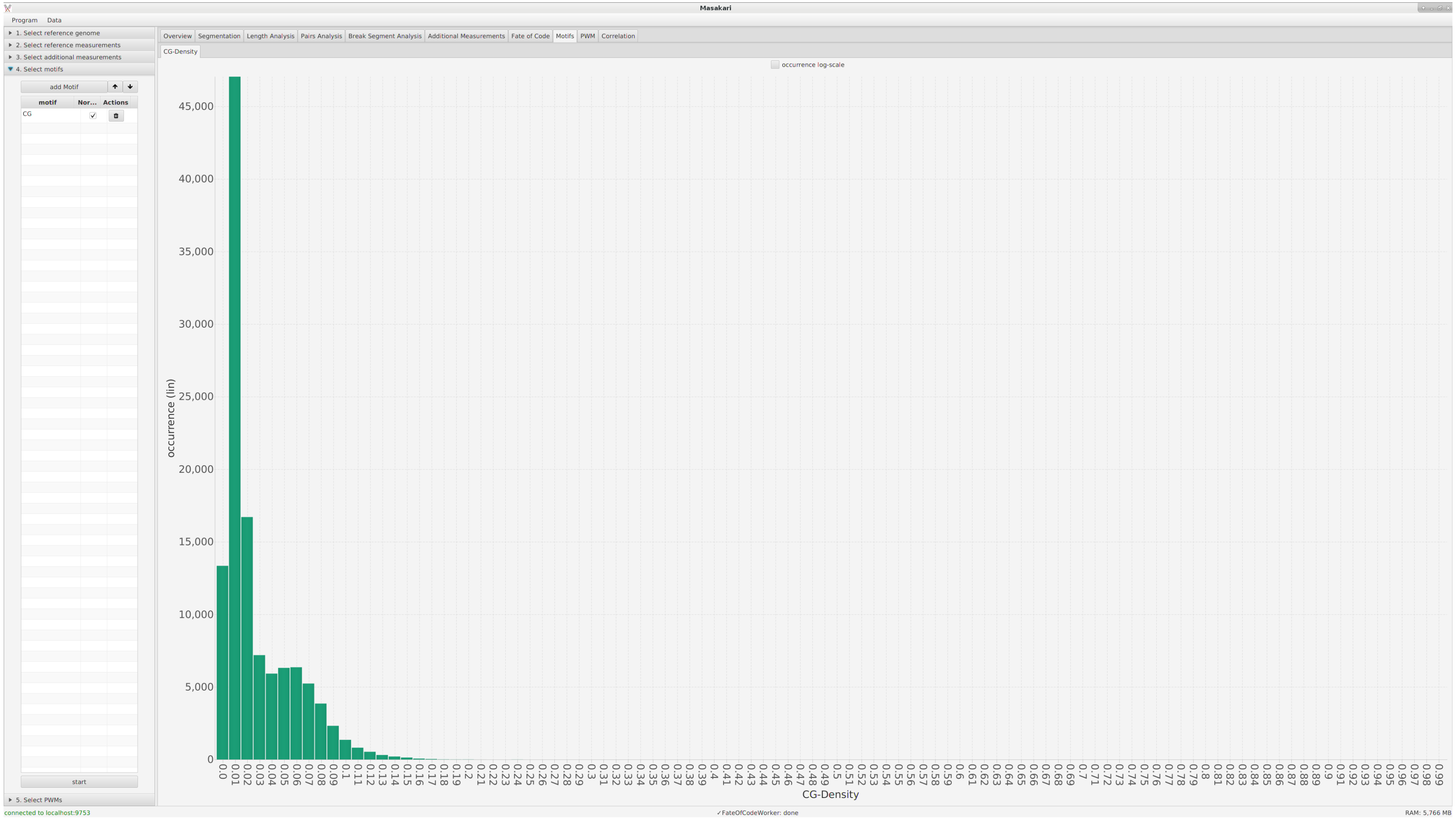

Figure 1.23: Motifs – CG-Density: This histogram shows the results of counting the CG occurrences and computing their density in the segments. The densities are binned and the number of segments having densities in a specific bin is shown by a bar. The number of occurrences can be scaled logarithmically ("occurrence log-scale").

1.7 Position Weight Matrix Coverage Analysis

1.7.1 Method

A position weight matrix (PWM) can be used to determine the probability that a certain nucleotide sequence matches a particular property, e.g., that it is a CTCF binding site.

As for motifs, the reverse complement of the PWM is constructed to cope with the not explicitly represented strand of the DNA. Then, the PWM probability is computed for each range of nucleotides of a segment. The result is a set of probabilities. The user can choose between computing the medium or the maximum probability of this set. Alternatively, the user can set a cut-off value (minimum probability) to get the count or the density of ranges having a probability above the cut-off probability. For the chosen alternative, the distribution over all segments is computed.

As for computing the motif coverage of a segment, this operation is quite time consuming due to reading the nucleotide sequences of the segments from external storage (because of the size of the reference genome, it is not stored in internal memory). Therefore, the search is not performed one PWM after another. Instead, the nucleotide sequence of each segment is loaded exactly once and then the probabilities for all selected PWMs are computed. Moreover, computing these probabilities is done in parallel depending on the available resources (CPUs and threads) of the hardware used.

After computing the coverage of the segments by the PWMs, the segmentation data table is extended by the obtained coverage value (row) for each of the PWMs.

1.7.2 Selection

A new position weight matrix is added by pressing the "add PWM" button (Figure 1.25). Then, a dialog is shown (Figure 1.24) that allows selecting a file by pressing the "Select Matrix" button. The file name and the path of the file selected are shown in the dialog. The upper set of radio buttons allows to choose the computation method: "Median", "Maximum", and "Cutoff". If "Cutoff" is selected then the lower set of radio buttons allows to choose between "normalized (density)" and "raw (count)" values, and the "Cutoff Value" can be entered. Pressing the "Add" button closes the dialog and adds the PWM to the table. Pressing the "Cancel" button ends the dialog without adding the PWM.

A table shows the file name of the PWM, the computation method, whether values are normalized or not (checkbox), the cutoff value, and an action button allowing to remove the PWM. The order of the PWMs can be changed using the move-up and move-down buttons. Pressing the "start" button starts the computation of the PWM coverage.

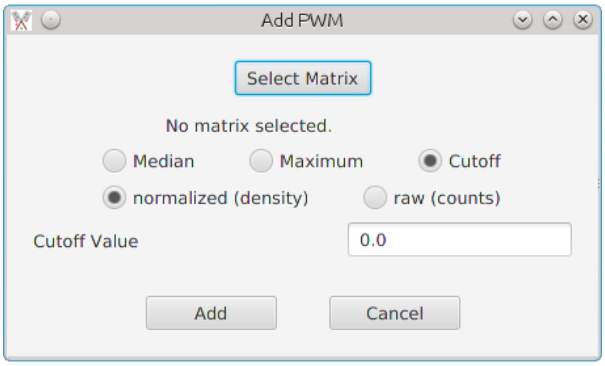

Figure 1.24: Add PWM dialog: It allows selecting a file by pressing the "Select Matrix" button. The name of the file selected is shown in the dialog. The upper set of radio buttons allows to choose the computation method: "Median", "Maximum", and "Cutoff". If "Cutoff" is selected then the lower set of radio buttons allows to choose between "normalized (density)" and "raw (count)" values, and the "Cutoff Value" can be entered. Pressing the "Add" button closes the dialog and adds the PWM to the table. Pressing the "Cancel" button ends the dialog without adding the PWM.

1.7.3 Visualization

Depending on the choice of the measurements chosen for each segment, a histogram is provided showing one of the following distributions:

- *Median*: distribution of the median probabilities of all segments (Figure 1.25)
- *Maximum*: distribution of the maximum probabilities of all segments (Figure 1.26)
- *Cut-off Count*: distribution of the number of probabilities above the cut-off value of all segments (Figure 1.27)
- *Cut-off Density*: distribution of the density of probabilities above the cut-off value of all segments (Figure 1.28)

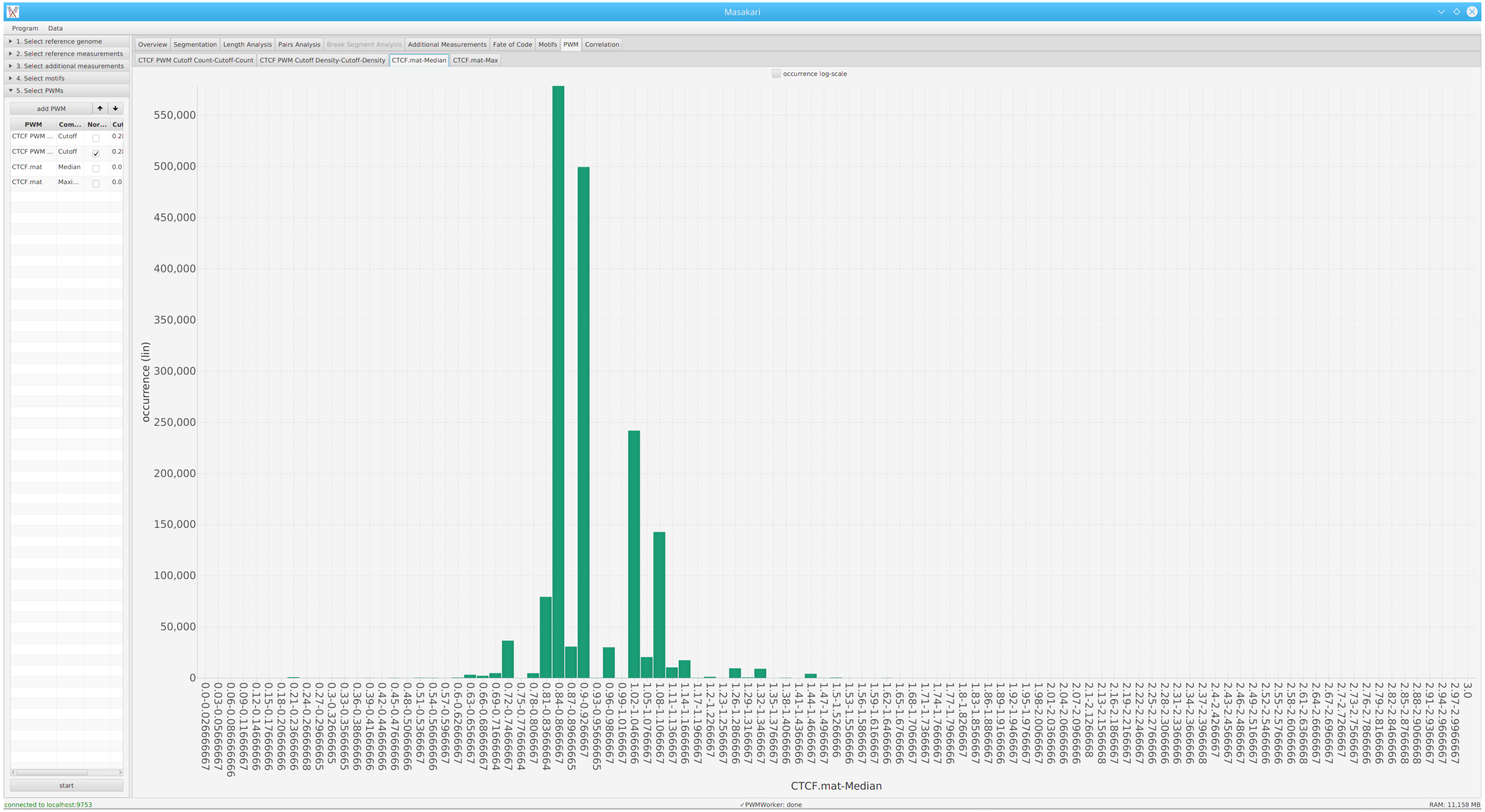

Figure 1.25: *PWM – Median*: The histogram shows the distribution of the median probabilities of all segments. As the number of occurrences can differ by orders of magnitude, it can be logarithmically scaled ("occurrence log-scale").

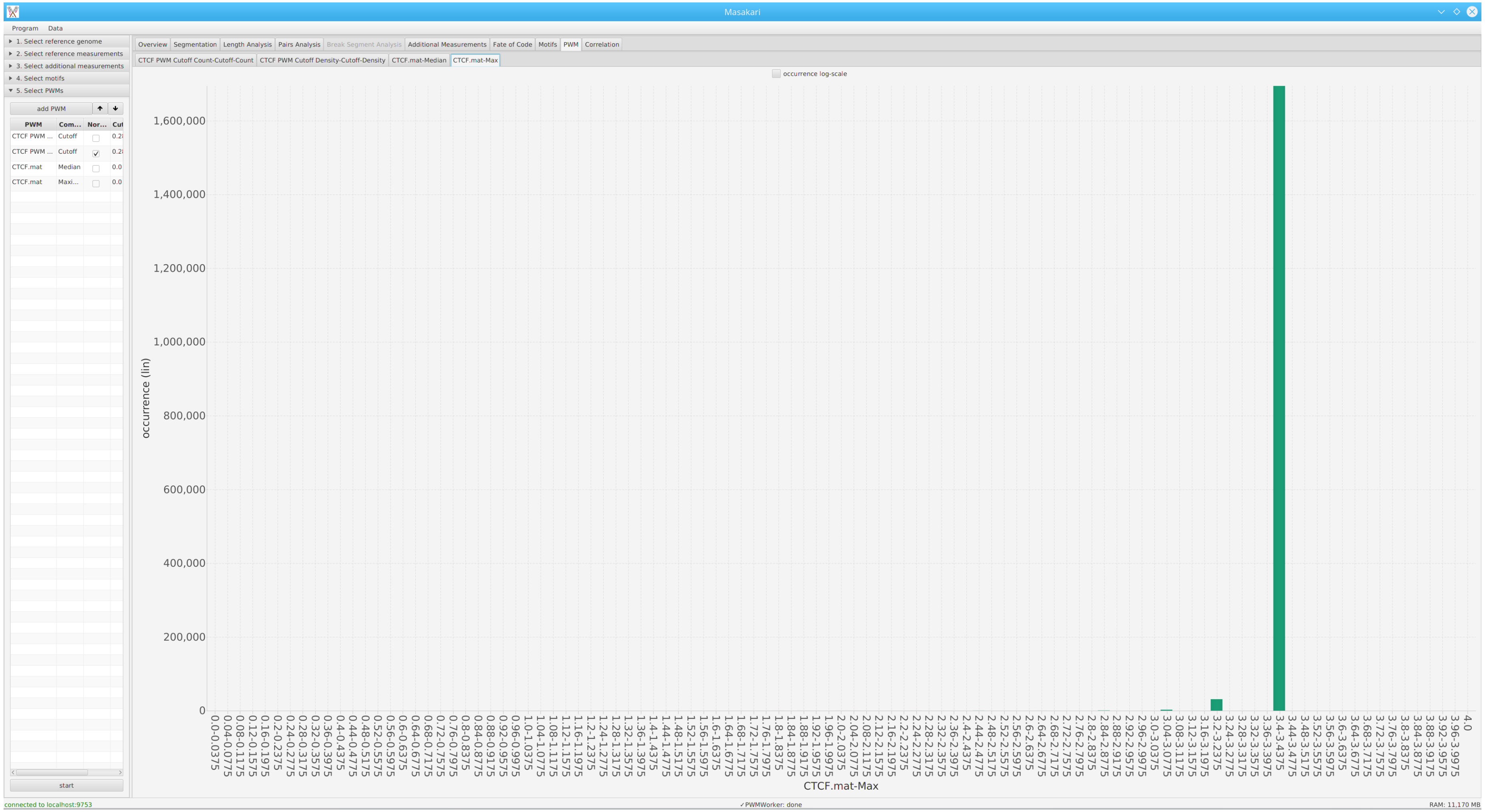

Figure 1.26: *PWM – Max*: The histogram shows the distribution of the maximum probabilities of all segments. As the number of occurrences can differ by orders of magnitude, it can be logarithmically scaled ("occurrence log-scale").

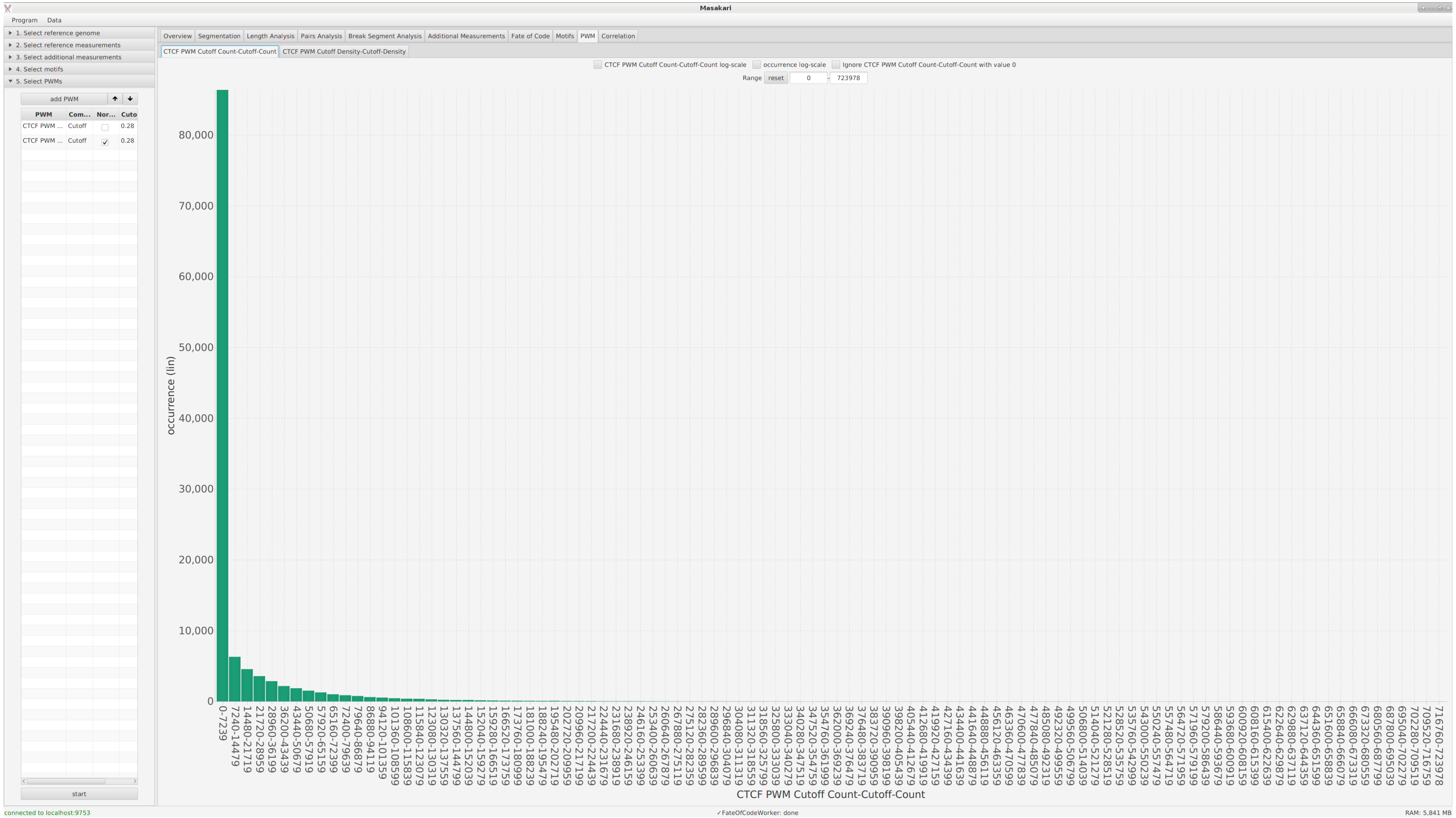

Figure 1.27: *PWM – Cutoff-Count*: The histogram shows the distribution of the number of probabilities above the cut-off value of all segments. As the number of occurrences can differ by orders of magnitude, it can be logarithmically scaled ("occurrence log-scale"). Moreover, the count can be logarithmically scaled ("Cutoff-Count log-scale"). As for motif coverage, the number of segments with count 0 can be dominant by orders of magnitude. Therefore, segments with count 0 can be ignored ("ignore Cutoff-Count with value 0"). The range of counts can be chosen by entering it into the number fields or by selecting a bar (double-click). Pressing the "reset" button will use the original range again.

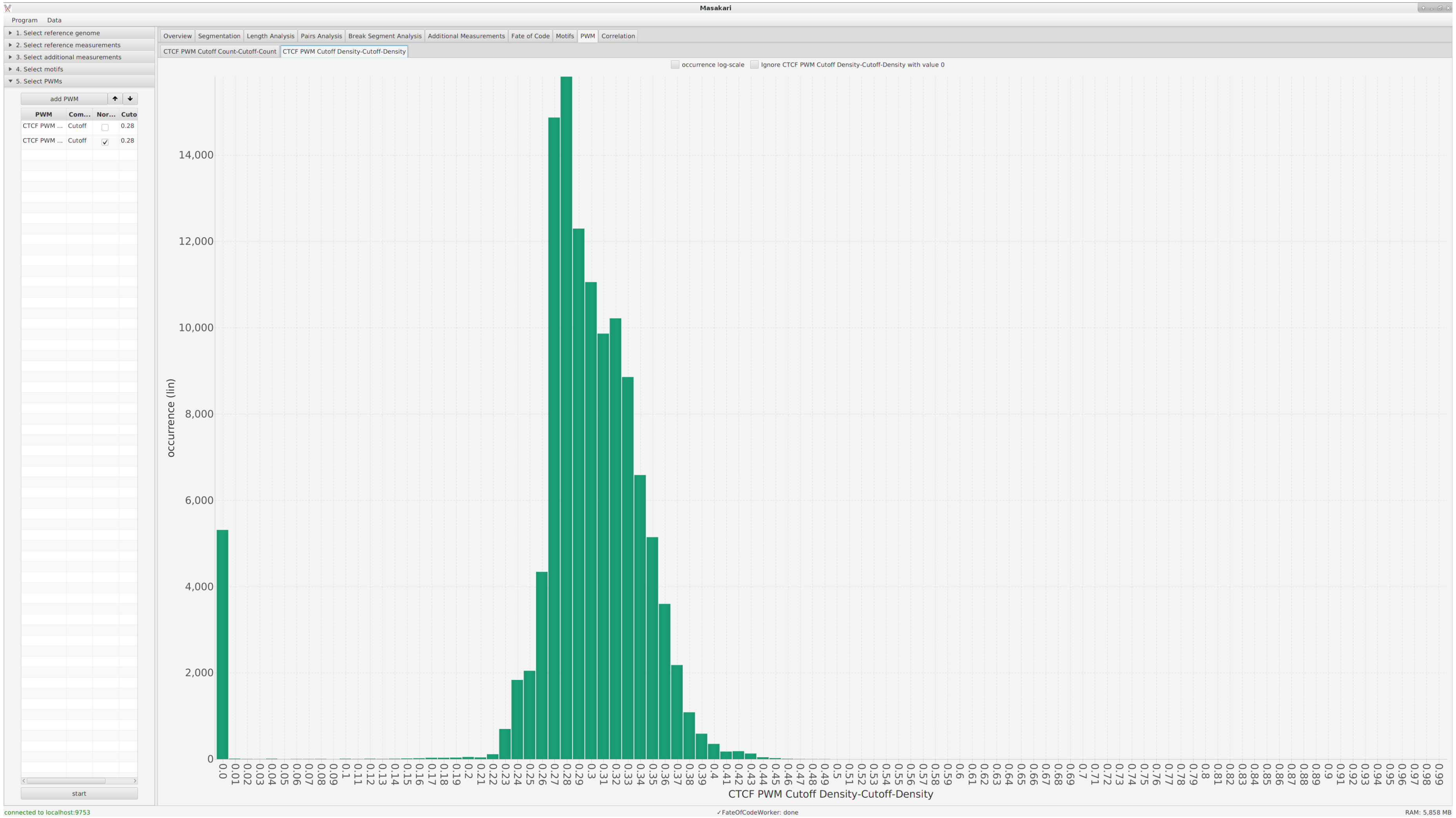

Figure 1.28: *PWM – Cutoff-Density*: The histogram shows the distribution of the number of probabilities above the cut-off value of all segments as density (number of probabilities divided by maximum number possible). As the number of occurrences can differ by orders of magnitude, it can be logarithmically scaled ("occurrence log-scale"). As the number of segments with count 0 can be dominant by orders of magnitude, segments with count 0 can be ignored ("ignore Cutoff-Count with value 0").

1.8 Correlation Analysis

1.8.1 Method

Spearman's rank correlation coefficient between code, reference measurements, additional measurements, motif coverage, and PWM coverage is computed using an optimized, parallelized version. As the data might contain many ties, the version taking equal values into account is used (Equation 1.4)

$$r_s = \frac{n^3 - n - \frac{1}{2}T_x - \frac{1}{2}T_y - 6\sum_i d_i^2}{\sqrt{(n^3 - n - T_x)(n^3 - n - T_y)}} \tag{1.4}$$

No additional information for the resulting segmentation data table is computed.

1.8.2 Selection

Pressing the "start" button (Figure 1.29), Spearman's rank correlation coefficient is computed between code, measurements, and coverages.

1.8.3 Visualization

The resulting correlation coefficients are stored in a table that is visualized as a heatmap (Figure 1.29).

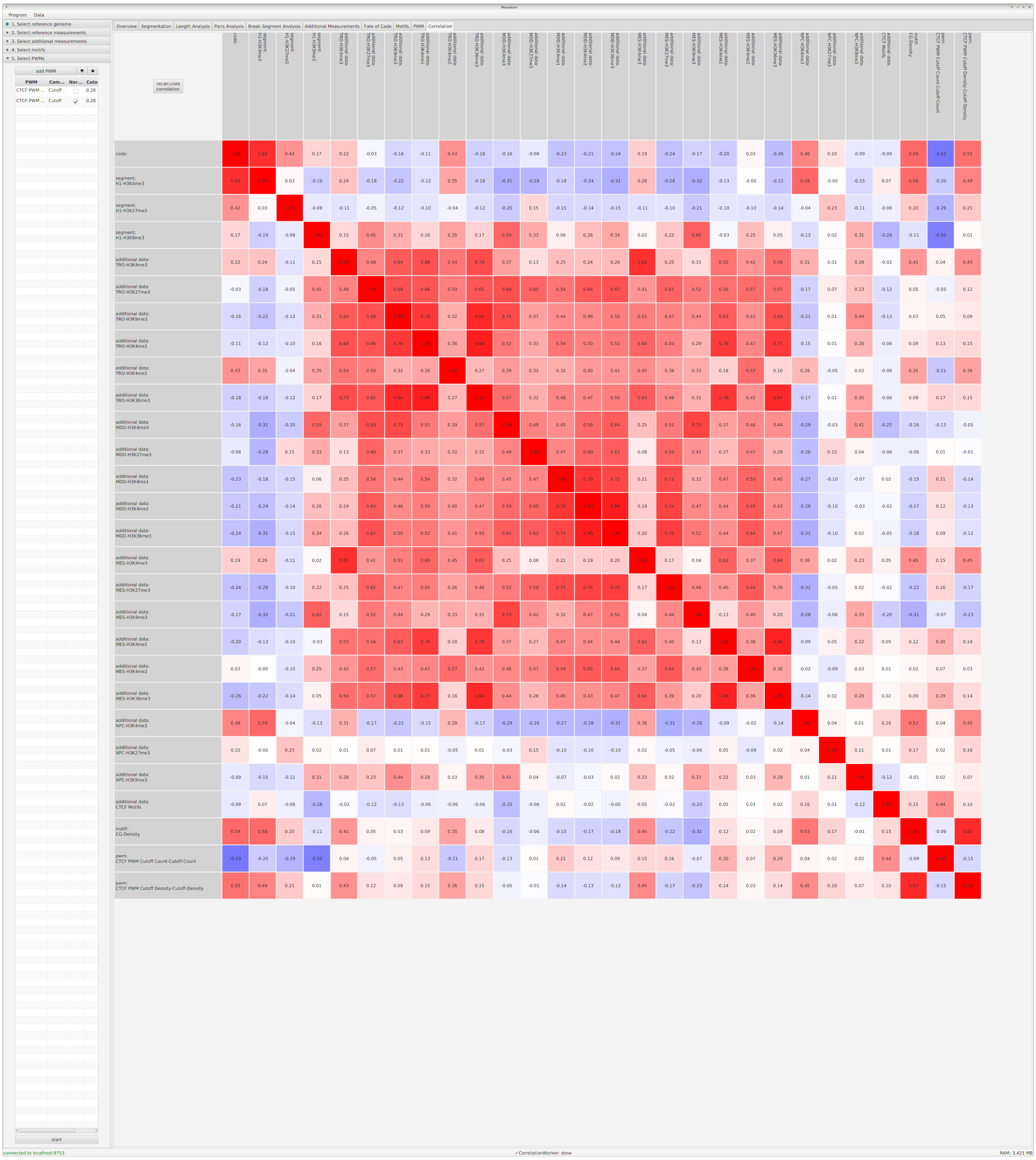

Figure 1.29: Correlation: A heatmap shows Spearman's rank correlation coefficients for code as well as reference measurements, additional measurements, motif, and PWM coverages. Highly saturated red means high positive correlation, highly saturated blue means high negative correlation, and white or desaturated colors indicate no or small correlation, respectively.

Chapter 2

Technical Details and Graphical User Interface

2.1 System

Masakari is completely written in JAVA 8 and uses JavaFX as API for the GUI. Therefore, it is possible to run Masakari on Windows, Linux, and Mac. The libraries used are Apache Commons IO [3], Apache Commons Logging [4], Apache Commons Math [5], Apache Commons Net [6], Apache Commons VFS [7], HTSJDK [1], JFreeChart [9], Gson [8], and JSch [10].

Choosing Java supports the generation of graphical user interfaces in a straight forward way. Moreover, its language concept based on software engineering principles supports maintainability.

To allow for providing segmentation using Masakari as-a-service, the program is split into two parts:

Masakari Server: performs all computations. It takes its parameters from the client and transfers the result to the client. The server is normally run on high-performance machines with sufficient resources, i.e., CPUs with several cores, a large amount of memory, and a high I/O bandwidth.

Masakari Client: the GUI that allows the user to select the data to use, to set and adjust the parameters, and to assess the results. It can be run on almost any current standard desktop computer.

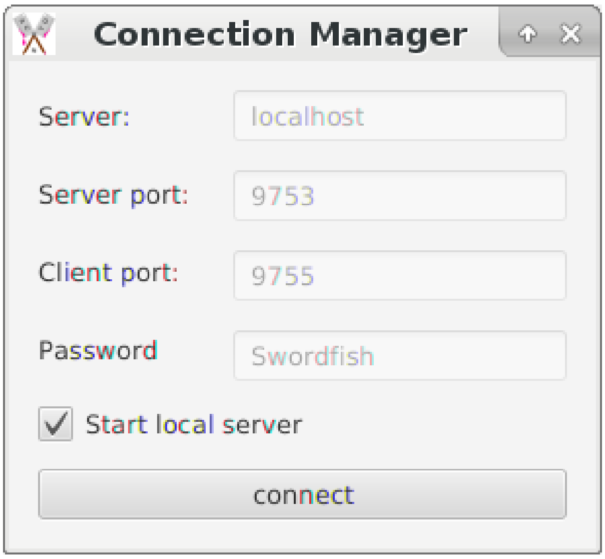

Figure 2.1: The server connection window of Masakari. The user can either start a local server (by ticking the checkbox as shown) or provide the connection details: server name, server port, client port, and password.

2.2 Masakari Server

The server can be started in server or in batch mode. In server mode, it accepts connections from clients. When the server is running a job, it is sending progress information to each client. Only one job can be run at a time, due to the large amount of resources needed. Therefore, the server is locked while performing the job, meaning, that it does not accept any additional jobs. Furthermore, it is possible to run the server in batch mode by providing a previously created job configuration file. In batch mode, the server will compute all results, store them in a file, and terminate afterwards.

2.3 Masakari Client

The client lets the user interact with the server for selecting the input data of the different steps of the process and for adjusting the parameters. A dashboard provides the possibility of a preliminary statistical analysis of the results.

2.3.1 Communication with Server

First, the Masakari Client is connected to a server using the connection dialog (Figure 2.1). If the calculation should be performed locally, since the data set is small or because the local computer has enough resources, the client can create it's own instance of the server ("Start local server" checkbox). Otherwise, the name of the server ("Host"), and two ports need to be provided: the "Server port" is the port on which the server is listening to connections and commands from potential clients, while the "Client port" is the port on which the client is listening to data received from the server. In principle, different clients on the same com-

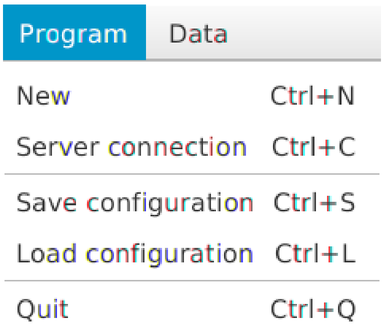

Figure 2.2: The Program Menu. The user can create a new, empty instance selecting 'New'. The server connection can be changed using 'Server connection'. The current configuration can be saved and a stored configuration can be loaded using 'Save configuration' and 'Load configuration', respectively. Finally, the program can be stopped using 'Quit'.

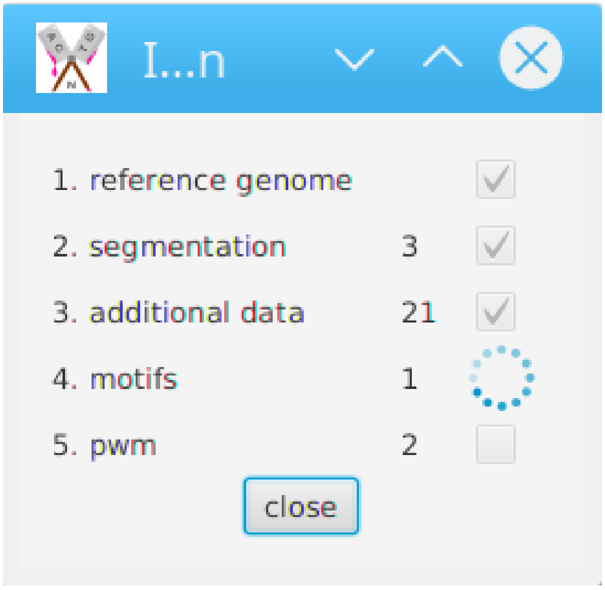

(a) This snapshot shows the time when the first three steps are finished shown by a ticked checkbox. The fourth step is currently executed shown by a set of rotating dots. The fifth step is waiting shown by the unticked checkbox.

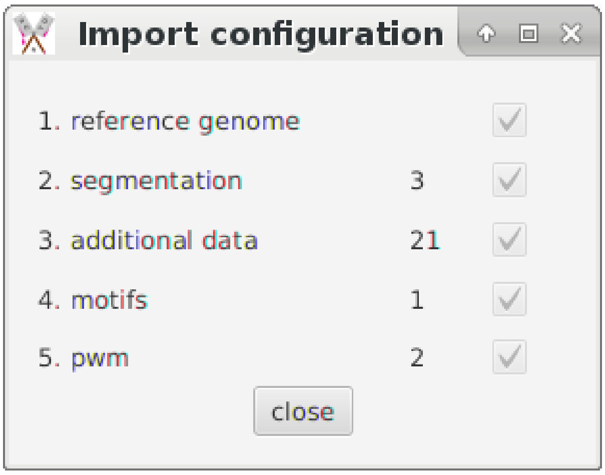

(b) This image shows the dialog when all computations are finished. In this case, all checkboxes are ticked.

Figure 2.3: The Import configuration dialog shows which steps of the process are performed (first two columns), how many data sets are involved for each step (third column), and the progress (last column). In the examples, all five steps are performed. The reference genome is always one. Here, three data sets are used for segmentation, and 21 additional data sets are provided. Overall, the coverage of one motif and of two position weight matrices (pwm) is computed. Figure 2.3a shows a snapshot while processing. Figure 2.3b shows the situation upon termination of the computations. The "close" button can be used any time to close the dialog and to examine the available results.

puter could use different "Client ports" connecting to different servers. Moreover, it is possible to secure the server with a password. After each successfully completed computation step, the server sends the results to each connected client and the client presents the data.

The connection dialog is opened automatically after starting the client. Further, a new connection can be opened any time using the Menu entry "Program → Server connection" (Figure 2.2).

2.3.2 Parameters and Starting Computation

The input data required and how to start the each computation are described in the "Methods" section (Section 1) together with the charts provided for the preliminary analysis.

2.3.3 Loading and saving the configuration

The currently active configuration can be saved by using the Menu Entry "Program → Save configuration" and loaded by using "Program → Load configuration" (Figure 2.2). When loading a configuration, the complete computation process is started immediately. This includes setting all parameters in the progress view. Figure 2.3a shows a snapshot of the computation progress while Figure 2.3b shows the dialog upon termination of all computations. The dialog can be closed any time by pressing the "close" button. Then, all available results can be examined. If the computation did not finish upon closing the dialog, it will continue after closing the dialog, and additional results can be examined as soon as they are available.

2.3.4 Export

After all computations are finished, the segmentation data table created can be exported by using the "Data → Export Table" menu entry (Figure 2.4). This allows the user to adjust the selections and to examine the results until she is satisfied.

The data saved has the following format. The first line contains the keyword "!Data". The second line contains the names of all columns created. These are at

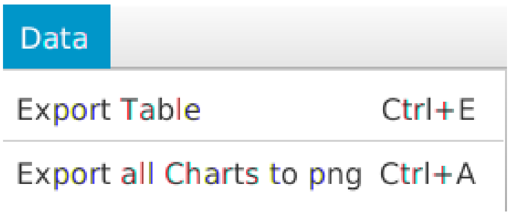

Figure 2.4: The Data menu provides export functionality. The segmentation data table as well as all charts created can be saved.

least the “shortId”, the “longId”, the “code”, and the “length” of each segment. If additional measurements, motifs, or PWMs were selected, then their descriptors are given here, too. All entries are separated by “;”. The third line contains the data types for each of the columns which are “String” for the shortId and the longId as well as “Integer” for code and length. All other fields are usually “Double”. Then follows one line per segment containing the respective column values.

Moreover, all figures created by Masakari can be exported as a bundle or as a single figure for later utilization as *.png* files. The user can export all figures by using the Menu Entry “Data → Export all Charts to png” (Figure 2.4). Single figures can be exported by clicking them with the right mouse button and choosing a filename.

Chapter 3

Results: Data, Data Preparation, and Additional Results and Figures

3.1 Cell Line, Cell Types, and Modifications

In the early stages of life, several tissues are developed which later differentiate further into organs and other tissues. This development is known as embryogenesis and is under tight control. Thus, strong epigenetic changes can be expected during this development.

H1 is a male human embryonic stem cell line. All other cells mentioned below are derived *in vitro* from this H1 cell line. Thus, H1 represents the origin of any other cell type in this study and is the natural starting point for the segmentation.

Trophoblast cells (TRO) are the first cell type developed from the fertilized egg emerging between the 5th and the 12th day after fertilization *in vivo*. They form the outer layer of the blastocyst and are required for the nutrition of the embryo.

Mesendodermal cells (MDD) are a later stage of the embryogenesis than trophoblast cells but do not derive from them. They develop further into mesodermal cells and endodermal cells.

Mesenchymal stem cells (MES) are adult stem cells derived mainly from mesodermal but also from endodermal and ectodermal cells. They have various functions in the development, regeneration, and repair of organs and tissues. However, their native identity and function is still not completely known [16].

Neuronal progenitor cells (NPC) represent an early stage of neuronal cells. *In vivo*, they derive from the ectodermal cells after the 4th week of pregnancy. They develop into the cells of the nervous system.

H3K4me1 and H3K4me2 are important for the cell development and H3K26me3 plays a role in splicing and thus may highlight differential expression of isoforms.

3.2 Data Preparation

The raw data was downloaded from the NIH Roadmap Project (see Table 3.1, 3.3, 3.5, and 3.7 for the accession numbers of the data sets). After extraction, we converted the sra files into fastq files and clipped the adapters. After cleaning, the reads were mapped with segemehl [12] with an accuracy of 80% against the human reference genome hg19 [14]. After the mapping, we used SAMtools to convert the results into one bam file for each data set and merged the data sets that consisted of more than one input fastq file and sorted them by chromosomes. We used the Picard Tools [2] to remove PCR duplicates. Afterwards, we created an index file for each bam file with SAMtools.

We used Sierra Platinum [15] to generate peak calls for each cell type based on all available replicates. A window size of 200nt, a window offset of 50nt, and a p-value cutoff of 10^{-5} were used. For the adjustment based on the qualities of the replicates please refer to the tables 3.2, 3.4, 3.6, 3.8, and 3.10. We chose to call peaks using Sierra Platinum over the peaks by the NIH Roadmap Epigenomics projects, as the former are more reliable than the latter [15].

Table 3.1: GSM IDs for H1

	H3K4me1	H4K4me2	H3K4me3	H3K27me3	H3K9me3	H3K36me3	Input
Replicate 1	–	–	GSM469971	GSM466734	GSM605325	–	GSM605333
Replicate 2	–	–	GSM605315	GSM605308	GSM605327	–	GSM605339
Replicate 3	–	–	–	–	GSM818057	–	GSM667642
Replicate 4	–	–	GSM409308	GSM434776	–	–	GSM605334
Replicate 5	–	–	GSM433170	GSM433167	GSM433174	–	GSM433179
Replicate 6	–	–	GSM537681	GSM537683	–	–	GSM537682
Replicate 7	–	–	GSM432392	–	GSM450266	–	GSM450270
Replicate 8	–	–	GSM410808	GSM428295	GSM428291	–	GSM428289

Table 3.2: Settings in Sierra Platinum based on visual inspection of the quality of the replicates. *ID*: number of replicate given in Table 3.1, *Weight*: weight used, *off*: replicate excluded, – : replicate not available.

ID	H3K4me3	H3K27me3	H3K9me3
1	0.1	1	0.05
2	0.1	–	0.05
3	–	–	1
4	1	0.05	–
5	0.1	0.1	0.1
6	off	–	–
7	1	–	off
8	–	–	1

Table 3.3: GSM IDs for Mesendoderm

	H3K4me1	H4K4me2	H3K4me3	H3K27me3	H3K9me3	H3K36me3	Input
Replicate 1	GSM752977	GSM752981	GSM752983	GSM752968	–	GSM752972	GSM753446
Replicate 2	GSM752978	GSM752982	GSM753440	GSM752969	–	GSM752973	GSM753447
Replicate 3	–	–	–	GSM864036	–	–	GSM864037
Replicate 4	–	–	–	GSM864801	–	–	GSM864802

Table 3.4: Settings in Sierra Platinum based on visual inspection of the quality of the replicates. *ID*: number of replicate given in Table 3.3, *Weight*: weight used, *off*: replicate excluded, – : replicate not available.

ID	H3K4me1	H3K4me2	H3K4me3	H3K27me3	H3K36me3
1	1	1	1	1	1
2	1	1	1	1	1
3	–	–	–	0.1	–
4	–	–	–	0.1	–

Table 3.5: GSM IDs for Mesenchymal

	H3K4me1	H4K4me2	H3K4me3	H3K27me3	H3K9me3	H3K36me3	Input
Replicate 1	GSM753437	GSM818041	GSM767348	GSM767344	GSM753445	GSM753434	GSM767354
Replicate 2	GSM767347	GSM818042	GSM767349	GSM767345	GSM767352	GSM767346	GSM767353

Table 3.6: Settings in Sierra Platinum based on visual inspection of the quality of the replicates. *ID*: number of replicate given in Table 3.5, *Weight*: weight used, *off*: replicate excluded, – : replicate not available.

ID	H3K4me1	H3K4me2	H3K4me3	H3K27me3	H3K9me3	H3K36me3
1	1	1	1	1	1	1
2	1	1	1	1	1	1

Table 3.7: GSM IDs for Trophoblast

	H3K4me1	H4K4me2	H3K4me3	H3K27me3	H3K9me3	H3K36me3	Input
Replicate 1	GSM753435	GSM753438	GSM753441	GSM753430	GSM818053	GSM753432	GSM753448
Replicate 2	GSM753436	GSM753439	GSM753442	GSM753431	GSM818054	GSM753433	GSM753449
Replicate 3	–	–	GSM906405	GSM896164	GSM906408	GSM906401	GSM864803

Table 3.8: Settings in Sierra Platinum based on visual inspection of the quality of the replicates. *ID*: number of replicate given in Table 3.7, *Weight*: weight used, *off*: replicate excluded, – : replicate not available.

ID	H3K4me1	H3K4me2	H3K4me3	H3K27me3	H3K9me3	H3K36me3
1	1	1	1	1	1	1
2	1	1	1	1	1	1
3	–	–	0.5	0.5	0.5	0.5

Table 3.9: GSM IDs for H1-NPC

	H3K4me1	H4K4me2	H3K4me3	H3K27me3	H3K9me3	H3K36me3	Input
Replicate 1	–	–	GSM1013151	GSM956010	–	–	GSM956029
Replicate 2	–	–	GSM818043	GSM896165	GSM1013158	–	GSM818063
Replicate 3	–	–	GSM767351	GSM818033	GSM818056	–	GSM767356
Replicate 4	–	–	GSM767350	GSM818032	GSM818055	–	GSM767355

Table 3.10: Settings in Sierra Platinum based on visual inspection of the quality of the replicates. *ID*: number of replicate given in Table 3.9, *Weight*: weight used, *off*: replicate excluded, – : replicate not available.

ID	H3K4me3	H3K27me3	H3K9me3
1	1	1	1
2	1	1	1
3	1	1	1
4	1	1	1

3.3 Short Segments Chains

We segmented the human genome version hg19 based on data from embryonic stem cells. For the embryonic stem cell line H1, the trimethylations of histone H3 at lysine K4, lysine K27, and lysine K9 were mapped and peak called. The main results are presented in the main manuscript.

Here, we show additional figures for the short segments chains. Figure 3.1a shows the distribution of the chains of the short segments according to the number of consecutive segments. Figure 3.1b depicts the length distribution of the short segment chains and Figure 3.2 the length distribution of the short segments. Given the results of all three figures, we judge that the segmentation is meaningful and, if at all, only weakly biased by the segments removed from segmentation.

Analyzing the modification changes of segment – short segment – segment triplets in the segmentation of the H1 data using Masakari, only one triplet with two segments carrying the same combination of modifications and being interrupted by a short segment is found (Figure 1.17). This points to an unproblematic segmentation.

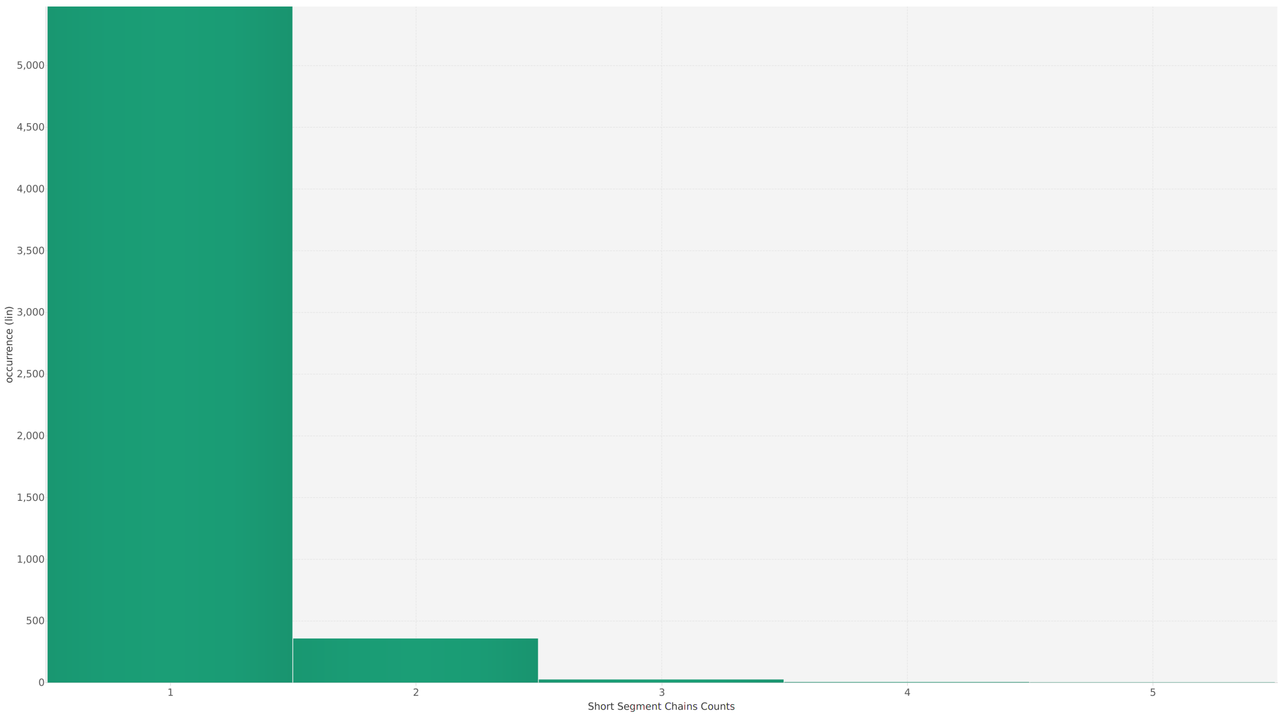

(a) *Number of consecutive short segments in a short segments chain*: The number of consecutive short segments is mapped onto the x-axis while its frequency is mapped onto the y-axis. Most chains consist of only one short segment and only about 600 chains are sequences of two or more short segments, i.e., contain different combinations of modifications. The maximal number of consecutive short segments is five.

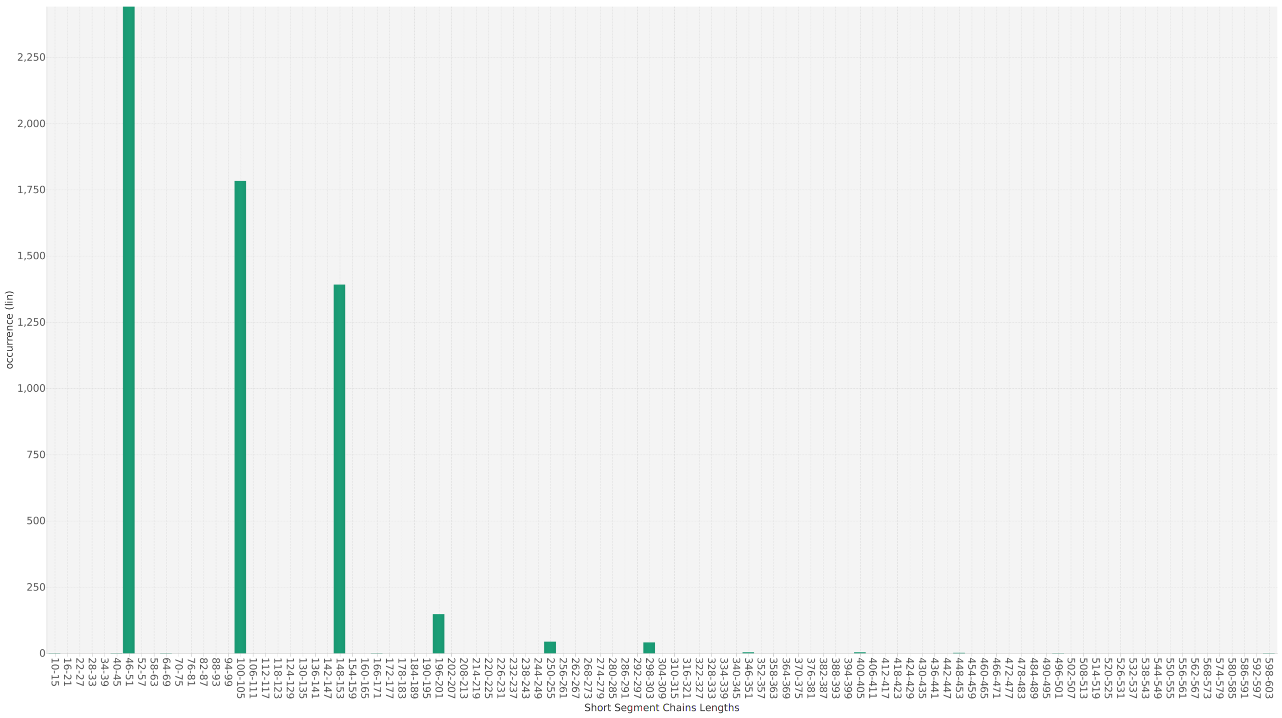

(b) *Length of short segments chains*: The length of the chains is given in nucleotides. It is binned and the bins are mapped onto the x-axis while the frequency of such a chain length is mapped onto the y-axis. Most chains are shorter than 200 nucleotides and no chain spans more than 600 nucleotides.

Figure 3.1: Chains of short segments for the segmentation results of the three modifications in H1 cells are analyzed. Top (a): number of consecutive short segments in a short segments chain and their frequencies. Bottom (b): lengths of short segments chains and their frequencies.

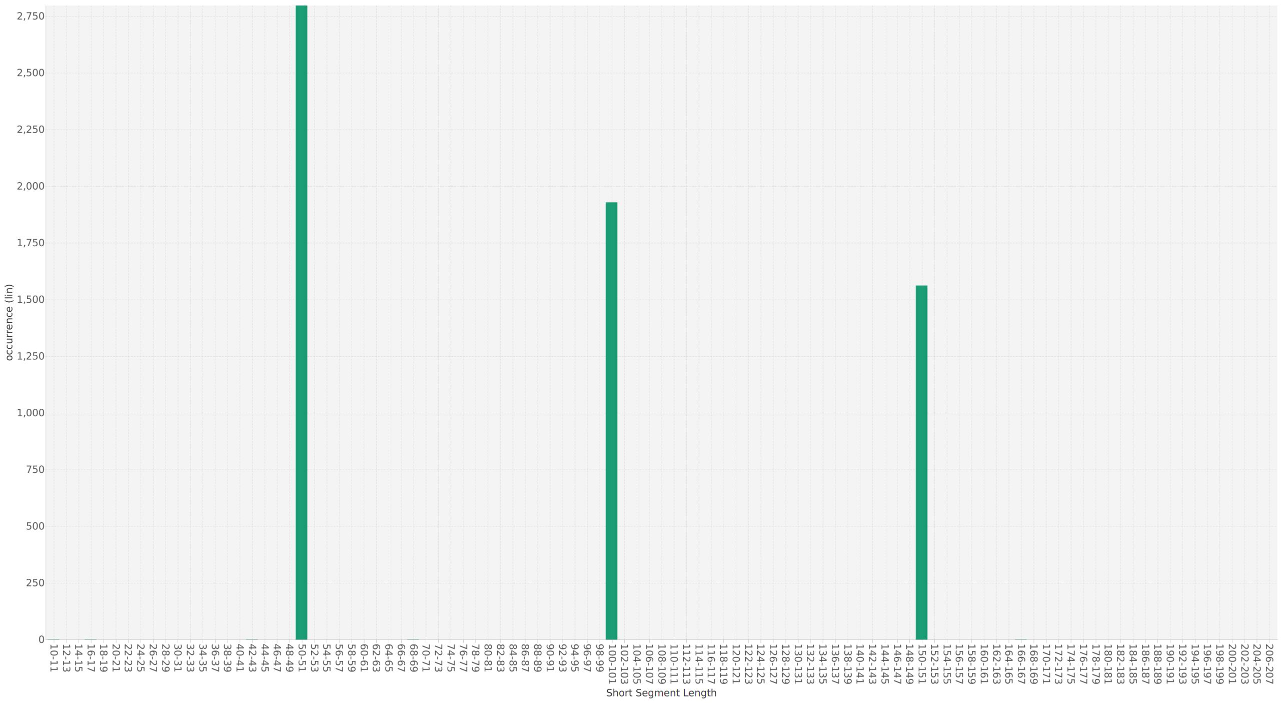

Figure 3.2: *Length of short segments*: The length of the short segments is mapped onto the x-axis while the frequency of such a segment length is mapped onto the y-axis. Again, the results of the segmentation of the three modifications in H1 cells is shown. As a consequence of the peak caller used, short segments are either 50, 100, or 150 nucleotides long.

3.4 Additional Data

In the main manuscript, we presented the distribution for the segment lengths and peak lengths. Here, we show the distribution for the additional data.

3.4.1 Modifications

Note, that all coverage values for the modifications range from 0.0 to 1.0. Values are binned uniformly and the frequency of each bin in the data is counted. The distributions of the different available modifications for the trophoblast cells, the mesendodermal cells, the mesenchymal stem cells, and the neuronal progenitor cells are shown in Figure 3.4, Figure 3.5, Figure 3.6, Figure 3.7, respectively.

The data for the mesendodermal cells and for the mesenchymal stem cells shows peaks at 0.0 and 1.0 as well as a peak around 6% coverage. The most frequent modification is H3K4me3 and all the other modifications seem to be less important. Please note, that there is no H3K9me3 data set for the mesendodermal cells. In mesenchymal stem cells, all modifications are reduced in frequency. Either other modifications play an important rôle in mesenchymal stem cells or the methylation level is decreased in general. In neuronal progenitor cells, the methylation level is low for all modifications. Nevertheless, one can still see a peak at 1.0 and a peak at 0.0.

3.4.2 CTCF

We furthermore investigated the presence of CTCF binding sites based on a data set from the Encode Project [11]. We downloaded the broad peaks from the GEO website. The binding sites we used were measured in cells of the H1 cell line and have the accession number GSM733672. They mostly show a low and medium coverage of the segments (see Figure 3.8).

However, binding sites were determined based on experimental data and human genome version hg19. Consequently, it covers only binding sites bound to CTCF in H1 cells in the experiment. Thus, we used the position weight matrix published by Kim et al. [13] to predict potential binding sites. Indeed several segments with many potential binding sites (Figure 1.28) or segments with a relatively high coverage with CTCF binding sites (Figure 1.27) can be found. If CTCF acts as chromatin domain insulator, then the observed domains and segments may be re-organized in later developmental stages. The correlation with the histone modification shows a positive and a negative correlation with histone marks in the different tissues (see Figure 3.3, rightmost column). Therefore, it is very likely that the predicted binding sites are either not bound or CTCF does not always act as insulator when bound to a binding site, as observed in literature.

3.4.3 Fate-Of-Code

For a meaningful analysis of the overlap of the additional data segments should be mainly either covered completely or not at all by peaks of the additional data sets (see Figures 3.4–3.7). For the additional data of trophoblast cells, strong peaks at 0 and 1 coverage can be observed. There are many H3K4me3 covered segments which seems to be particularly important for trophoblast cells. All modifications show an additional peak around 6% coverage. Thus, it might be worth to further analyze a segmentation based on the trophoblast cells rather than on the H1 cells.

Segments unmodified in H1 cells usually stay unmodified during embryogenesis, i.e., there is no large systematic displacement of modifications to previously unmodified genomic regions. However, there is still a large number of segments which are H3K9me3 modified in TROs, MESs, or NPCs but not in H1. Thus, the data suggests to further investigate the segments covered now with H3K9me3 since they may lead to inactivation of transcription. Interestingly, in trophoblast cells we observe a larger number of segments with H3K9me3 and H3K4me3 marks. In mesenchymal stem cells, many segments with new H3K4me3 marks (previously unmarked) can be found. This is a sign for a possible activation of mesenchymal stem cell pathways.

A universal trend is that segments carrying only one mark often loose this mark. Also bivalent marks in H1 cells are frequently lost completely. Only in mesenchymal stem cells and in neuronal progenitor cells, bivalent segments turn into H3K4me3 marked segments, and thus, imply active transcription of associated genes. Segments with only H3K4me3 marks often retain this mark in TRO, MES, and NPC. They might be close to or within promoters of housekeeping genes. In trophoblast cells, about 3,000 segments marked with only H3K4me3 in H1 acquire additionally H3K27 and H3K9 trimethylation. This combination of marks is associated with repressed transcription and thus may indicate genomic regions with genes specific for embryonic stem cells.

H3K9me3 in H1 also correlates with many other modifications in the other cell types. Strong correlation is observed between the modifications of the cell types TRO, MDD, and MES as well as H3K27me3 and H3K9me3 in NPC. Thus, an in-depth analysis of the similarities between these cell types will be fruitful.

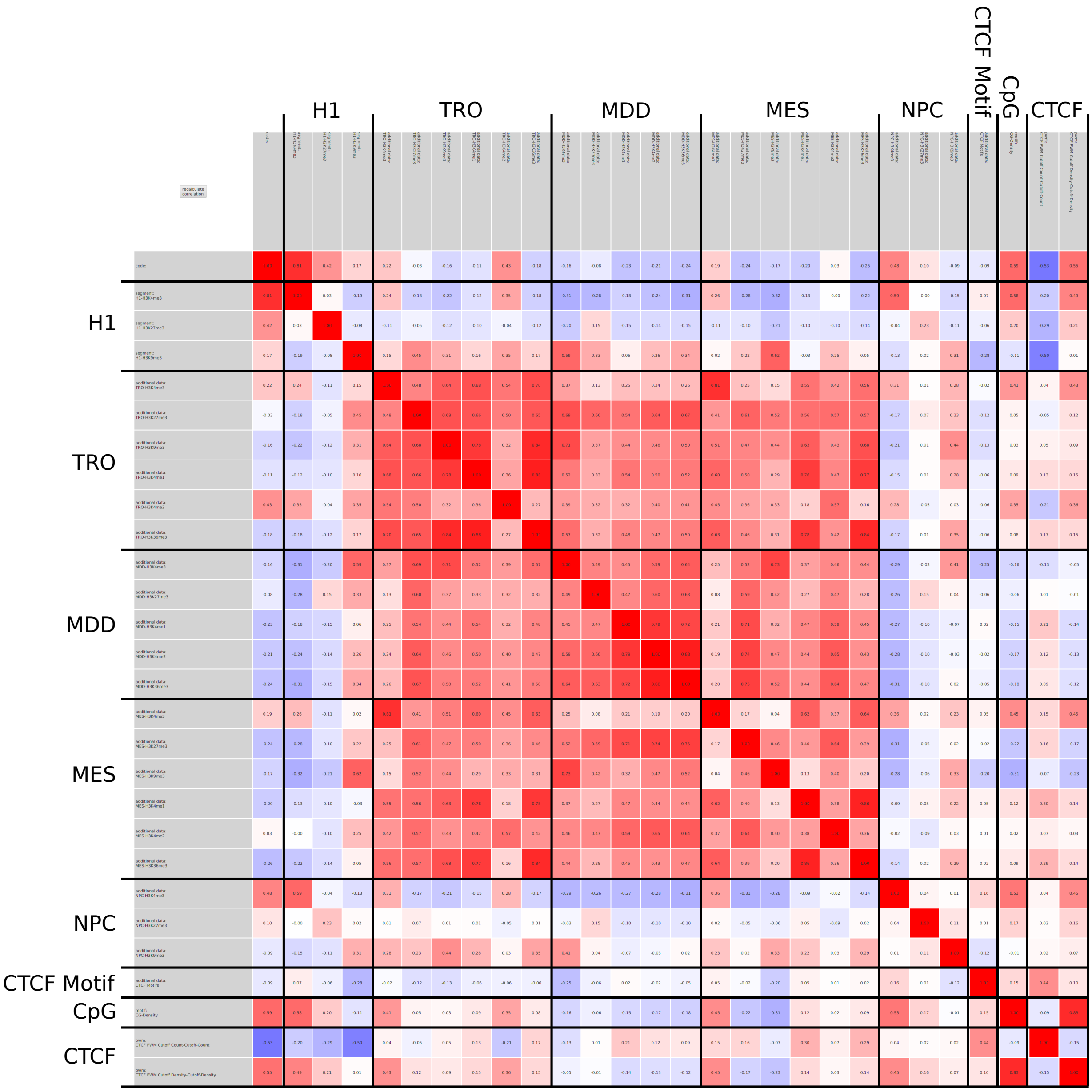

Figure 3.3: Correlation Analysis: Correlation between each pair of data sets as well as the code (first row and column) and the CpG-density (last row and column). Horizontal and vertical lines indicate blocks of modifications of the same cell types. Blocks of cell types are labeled before and above the blocks. Red: positive correlation, blue: negative correlation. High saturation: strong correlation; low saturation: weak correlation.

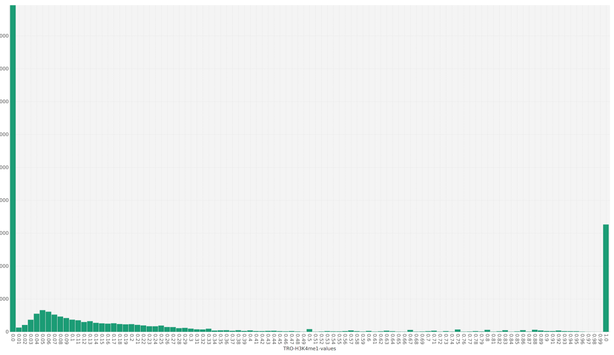

(a) Distribution of H3K4me1 in trophoblast cells

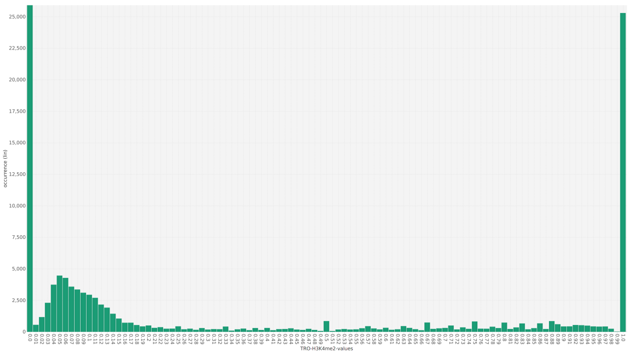

(b) Distribution of H3K4me2 in trophoblast cells

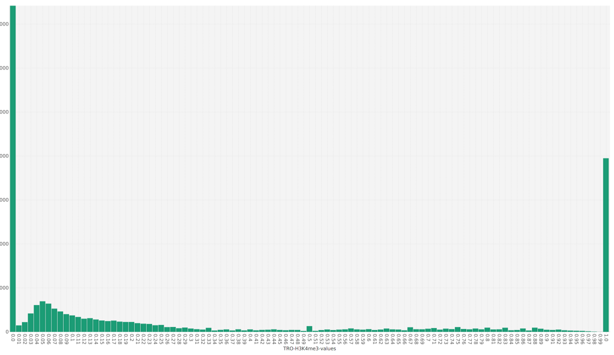

(c) Distribution of H3K4me3 in trophoblast cells

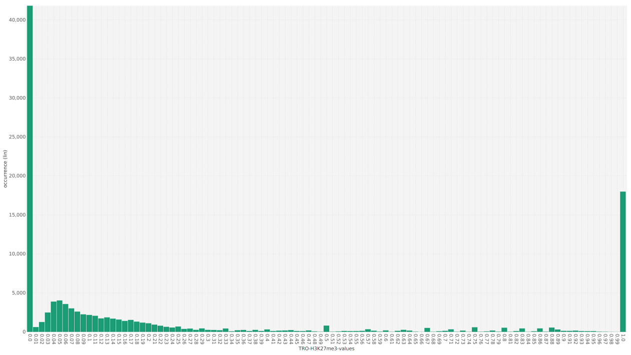

(d) Distribution of H3K27me3 in trophoblast cells

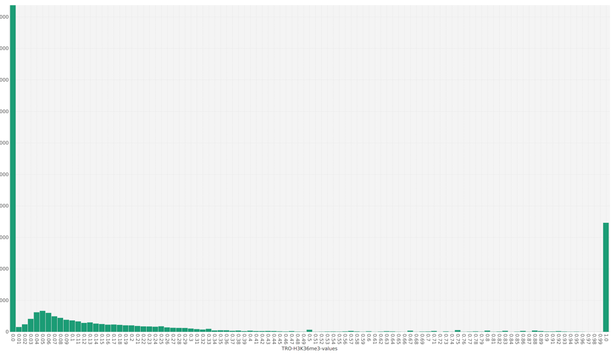

(e) Distribution of H3K36me3 in trophoblast cells

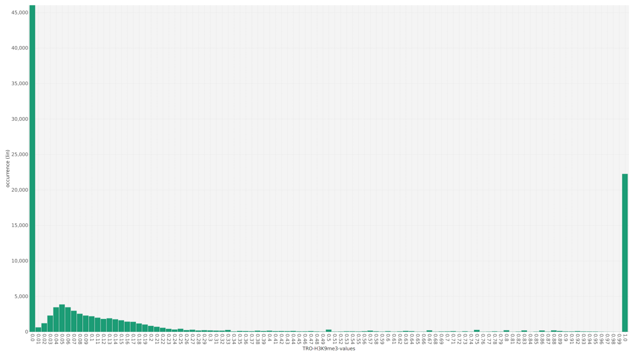

(f) Distribution of H3K9me3 in trophoblast cells

Figure 3.4: Distribution of the different modifications in trophoblast cells

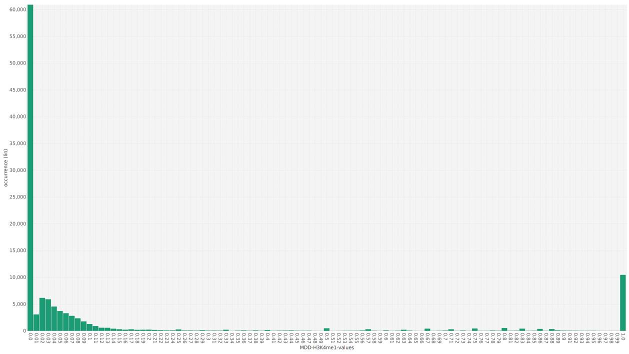

(a) Distribution of H3K4me1 in mesendodermal cells

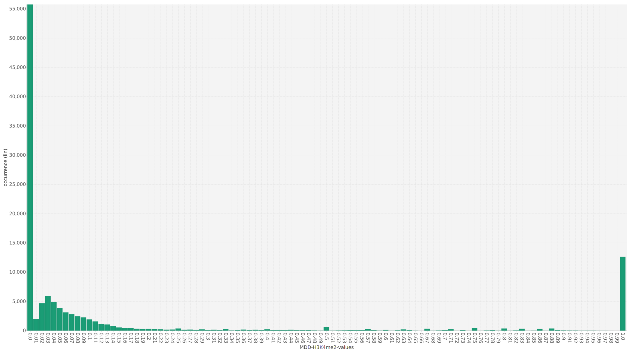

(b) Distribution of H3K4me2 in mesendodermal cells

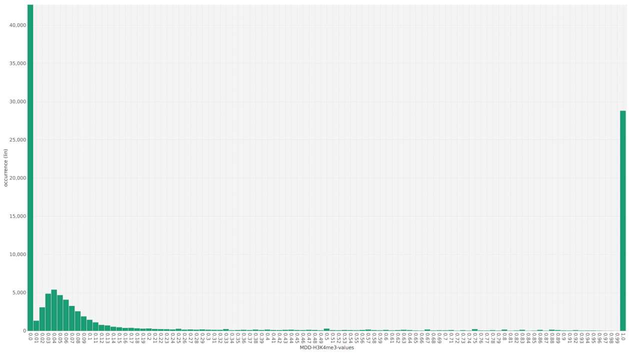

(c) Distribution of H3K4me3 in mesendodermal cells

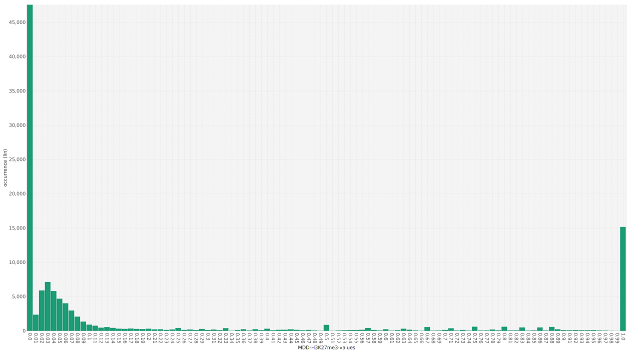

(d) Distribution of H3K27me3 in mesendodermal cells

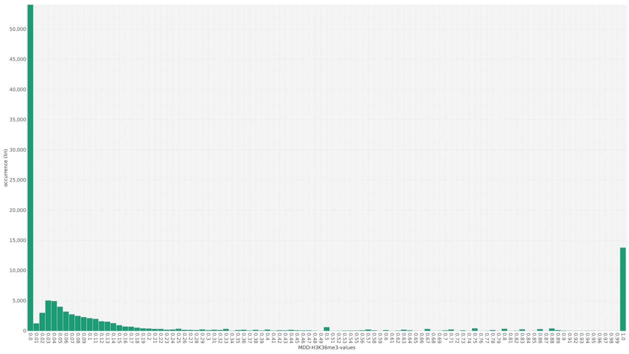

(e) Distribution of H3K36me3 in mesendodermal cells

Figure 3.5: Distribution of the different modifications in mesendodermal cells

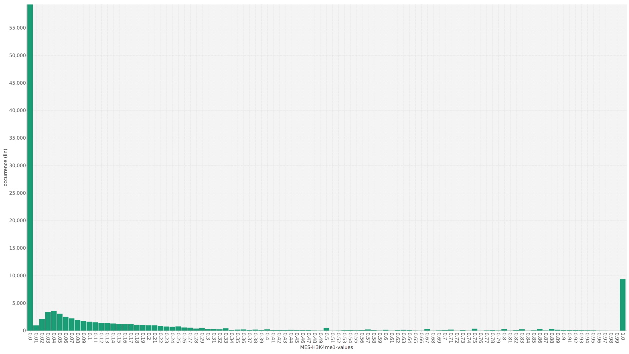

(a) Distribution of H3K4me1 in mesenchymal stem cells

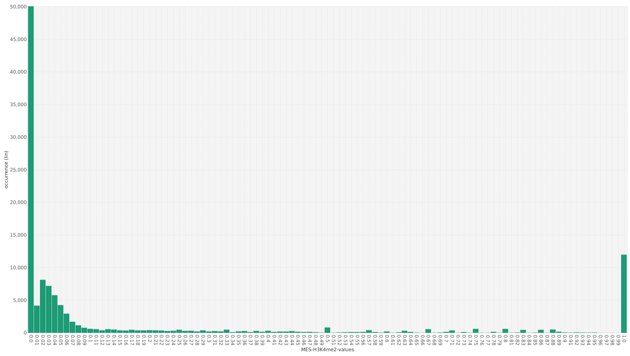

(b) Distribution of H3K4me2 in mesenchymal stem cells

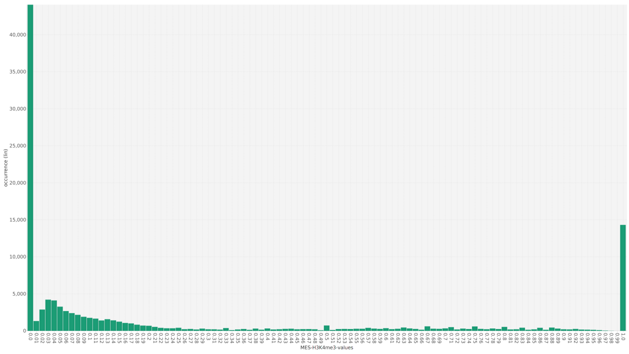

(c) Distribution of H3K4me3 in mesenchymal stem cells

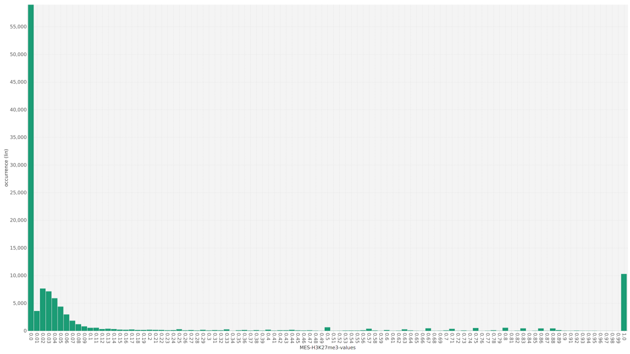

(d) Distribution of H3K27me3 in mesenchymal stem cells

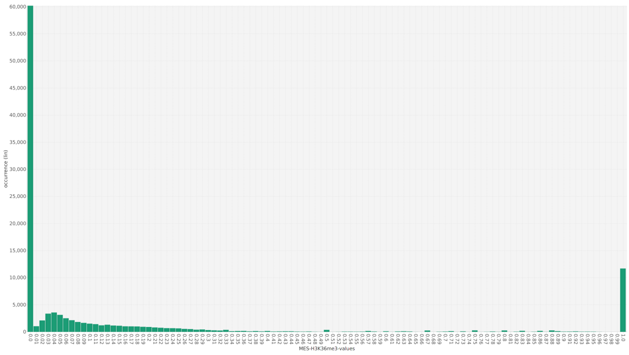

(e) Distribution of H3K36me3 in mesenchymal stem cells

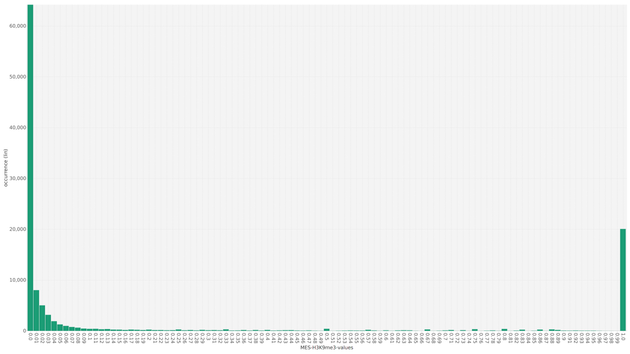

(f) Distribution of H3K9me3 in mesenchymal stem cells

Figure 3.6: Distribution of the different modifications in mesenchymal stem cells

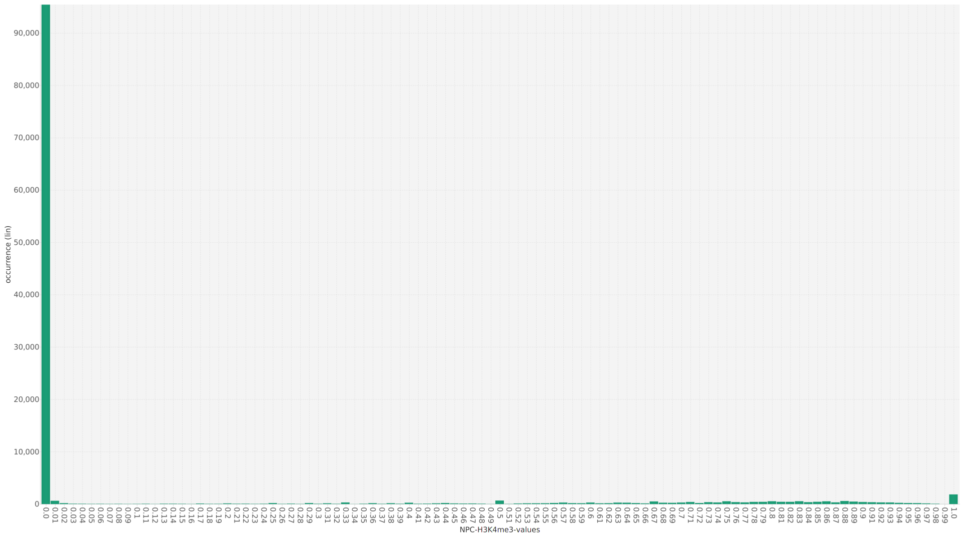

(a) Distribution of H3K4me3 in neuronal progenitor cells

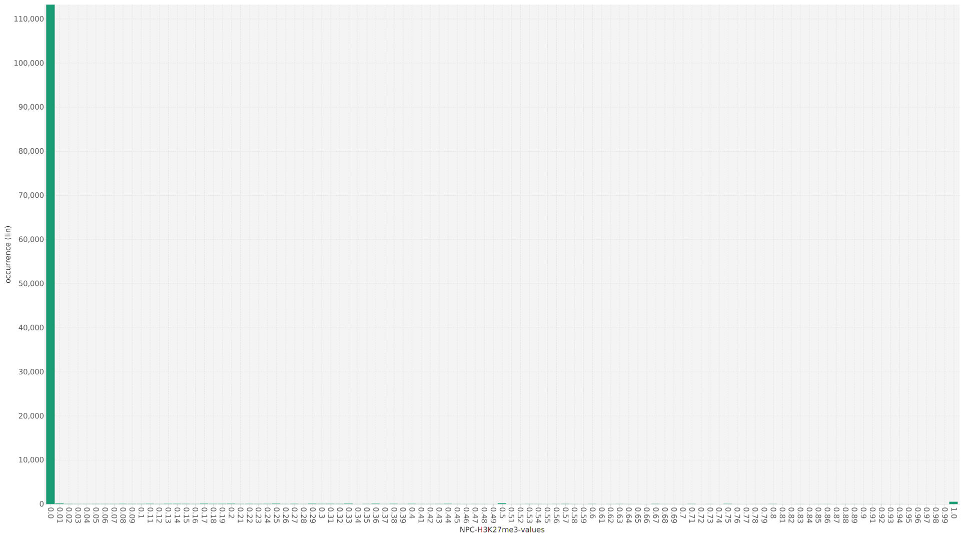

(b) Distribution of H3K27me3 in neuronal progenitor cells

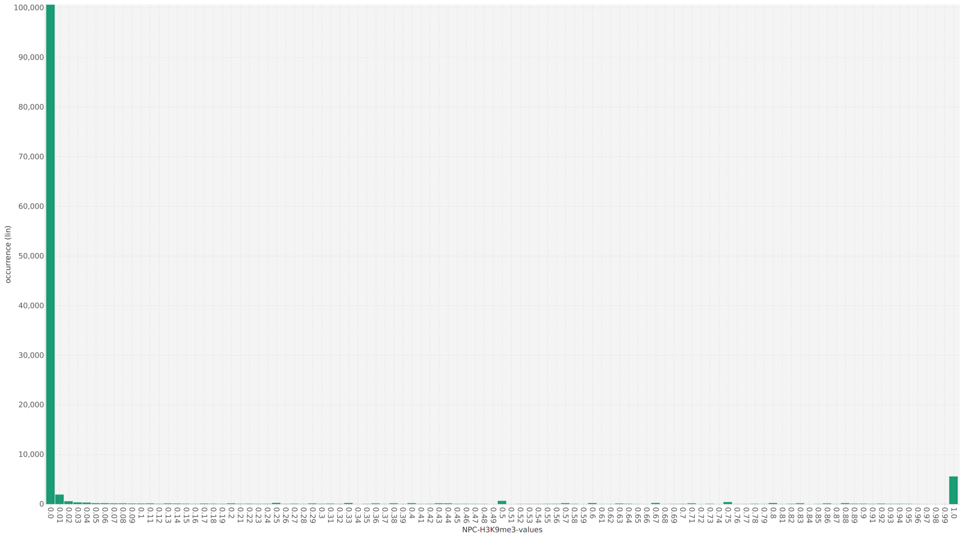

(c) Distribution of H3K9me3 in neuronal progenitor cells

Figure 3.7: Distribution of different modifications in neuronal progenitor cells

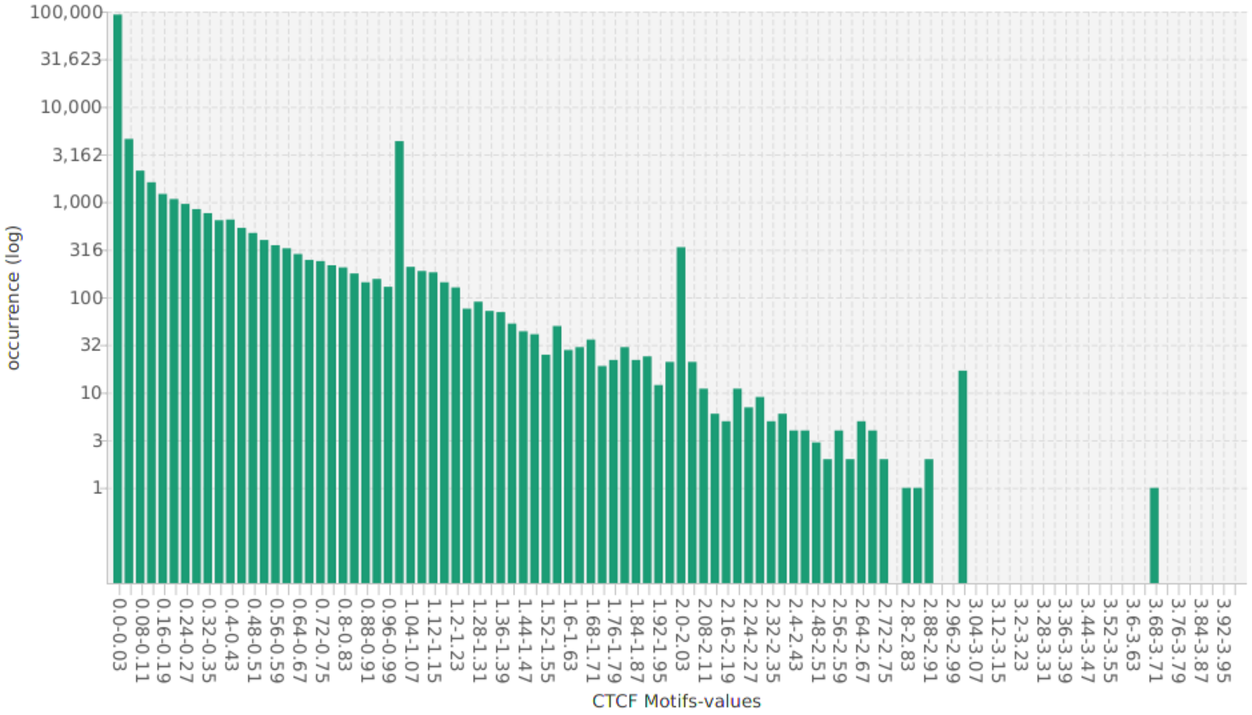

Figure 3.8: Distribution of the coverage with CTCF Motifs.

Bibliography

[1] A Java API for high-throughput sequencing data (HTS) formats. http://samtools.github.io/htsjdk/.

[2] A set of tools (in Java) for working with next generation sequencing data in the BAM (http://samtools.sourceforge.net) format. http://broadinstitute.github.io/picard/.

[3] Apache Commons IO. https://commons.apache.org/proper/commons-io/.

[4] Apache Commons Logging. https://commons.apache.org/proper/commons-logging/.

[5] Apache Commons Math. https://commons.apache.org/proper/commons-math/.

[6] Apache Commons Net. https://commons.apache.org/proper/commons-net/.

[7] Apache Commons VFS. https://commons.apache.org/proper/commons-vfs/.

[8] Google Gson. https://github.com/google/gson/.

[9] JFreeChart. http://www.jfree.org/jfreechart/.

[10] JSch - Java Secure Channel. http://www.jcraft.com/jsch/.

[11] ENCODE Project Consortium and others. An integrated encyclopedia of DNA elements in the human genome. *Nature*, 489(7414):57, 2012.

[12] Steve Hoffmann, Christian Otto, Stefan Kurtz, Cynthia M. Sharma, Philipp Khaitovich, Jörg Vogel, Peter F. Stadler, and Jörg Hackermüller. Fast mapping of short sequences with mismatches, insertions and deletions using index structures. *PLoS Computational Biology*, 5(9):e1000502, Sep 2009.

[13] Tae Hoon Kim, Ziedulla K. Abdullaev, Andrew D. Smith, Keith A. Ching, Dmitri I. Loukinov, Roland D. Green, Michael Q. Zhang, Victor V. Lobanenkov, and Bing Ren. Analysis of the vertebrate insulator protein ctcf-binding sites in the human genome. *Cell*, 128(6):1231 – 1245, 2007.

[14] Eric S. Lander, Lauren M. Linton, Bruce Birren, and et al. Initial sequencing and analysis of the human genome. *Nature*, 409(6822):860–921, Feb 2001.

[15] Lydia Müller, Daniel Gerighausen, Mariam Farman, and Dirk Zeckzer. Sierra platinum: a fast and robust peak-caller for replicated chip-seq experiments with visual quality-control and -steering. *BMC Bioinformatics*, 17(1):1–13, 2016.

[16] Iain R. Murray and Bruno Péault. Q&a: Mesenchymal stem cells — where do they come from and is it important? *BMC Biology*, 13(1):99, 2015.
